# Supplementary material for: DJ-1 suppresses ferroptosis through preserving the activity of S-adenosyl homocysteine hydrolase
Source: Nat Commun. 2020 Mar 6;11:1251. doi: 10.1038/s41467-020-15109-y (PMC7060199; doi:10.1038/s41467-020-15109-y)
Supplement: Supplementary file 1 — Supplementary Information [file 41467_2020_15109_MOESM1_ESM.pdf]

DJ-1 Suppresses Ferroptosis Through Preserving The Activity of S-adenosyl Homocysteine Hydrolase

Cao et al.

Supplementary Figure 1

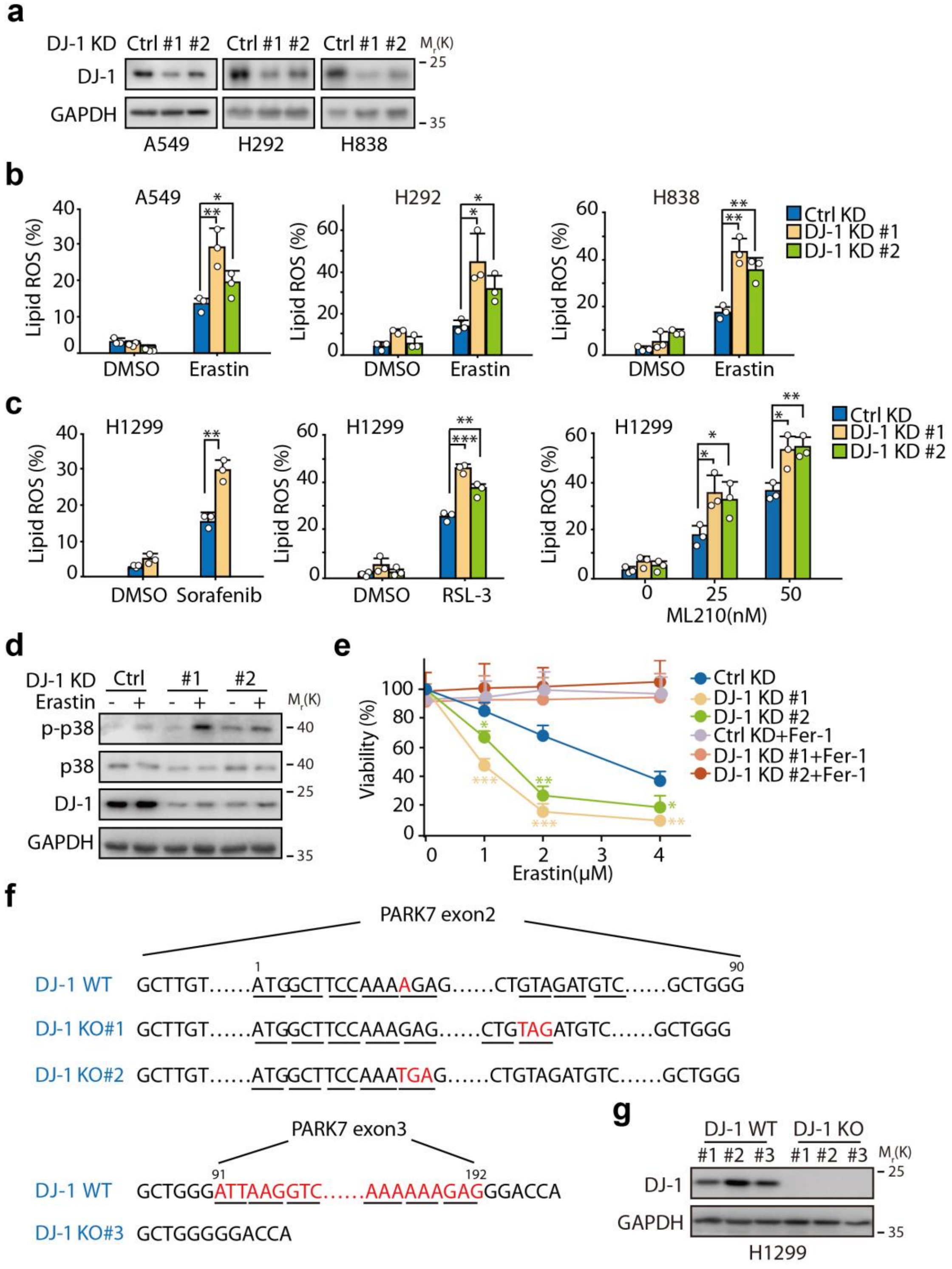

Supplementary Figure 1. a Western blot analysis of DJ-1 expression in indicated DJ-1 knockdown A549, H292 and H838 cells. Independent experiments are repeated three times and representative data are shown. **b** Indicated DJ-1 knockdown A549 (the

left view), H292 (the middle view), and H838 (the right view) cells were treated with Erastin (4 μM, 5 μM, 2.5 μM) for 24 h or 36 h, respectively, and lipid ROS production was assayed by flow cytometry using C11-BODIPY. **c** Indicated DJ-1 knockdown H1299 cells were treated with Sorafenib (5 μM) (the left view), RSL3 (50 nM) (the middle view), ML210 (25 μM, 50 μM) (the right view) for 12h, and lipid ROS production was assayed. **d** Indicated DJ-1 knockdown H1299 cells were treated with Erastin (2 μM) for 12 h and subjected to western blot analysis of the indicated proteins in whole cell extracts. The experiments were performed for triplicate and representative data are shown. **e** Cell viability was assayed in indicated DJ-1 knockdown H1299 cells treated for 36 h with Erastin (1-4 μM) with or without Fer-1 (1 μM). **f** The sequencing result of DJ-1 in H1299 KO cells mediated by CRISPR/Cas9. **g** Western blot analysis of DJ-1 expression in indicated DJ-1 WT and KO H1299 cells mediated by CRISPR/Cas9. Independent experiments are repeated three times and representative data are shown. Data shown represent mean ± SD from three independent experiments. Comparisons were made using the two-tailed, unpaired Student's t test. *: $p < 0.05$; **: $p < 0.01$; ***: $p < 0.001$.

Supplementary Figure 2

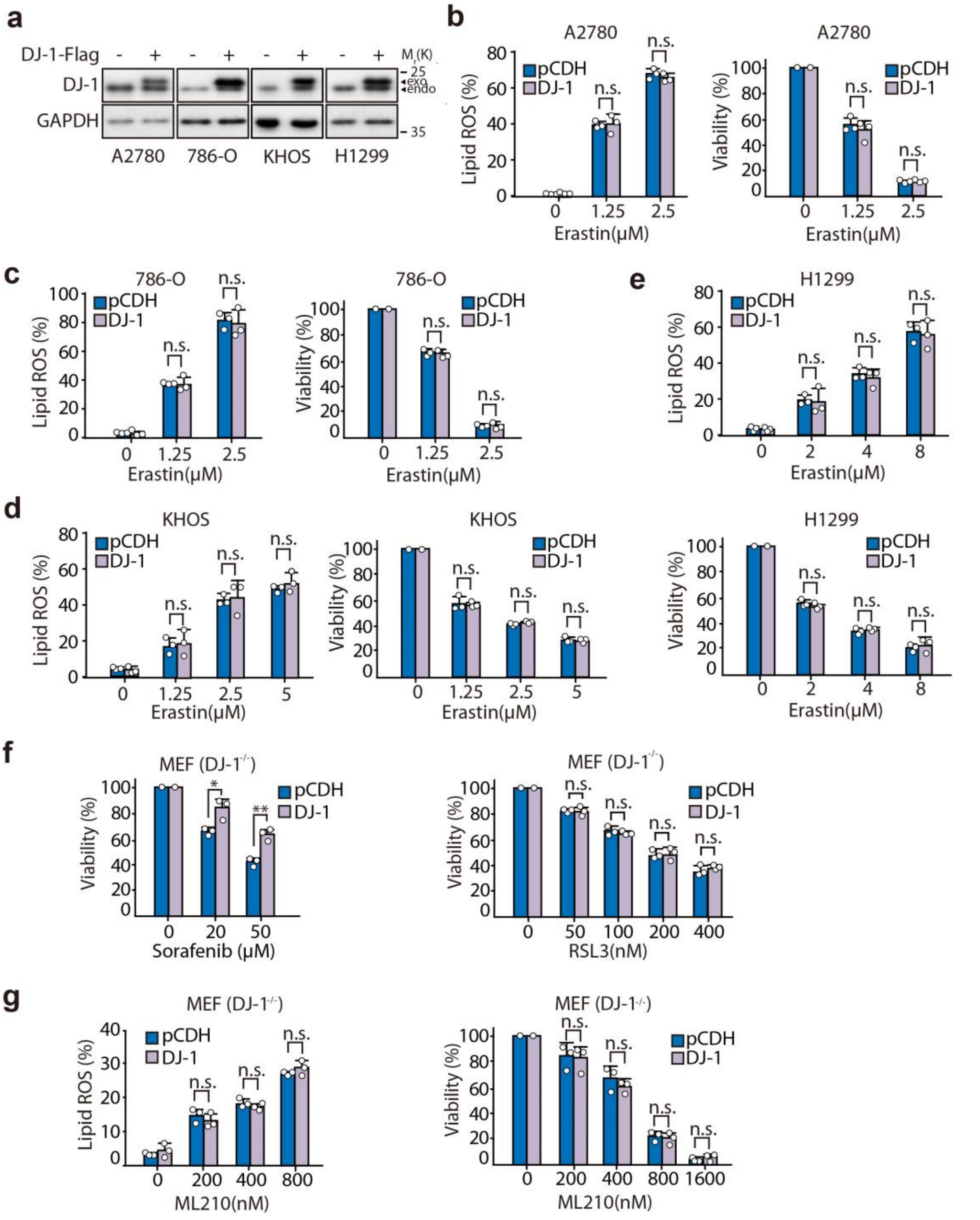

Supplementary Figure 2. a Western blot analysis of DJ-1 expression in indicated DJ-1 overexpression A2780, 786-O, KHOS and H1299 cells. Independent experiments are repeated three times and representative data are shown. **b-e** Indicated A2780, 786-O, KHOS and H1299 cells were treated with Erastin: 1.25 μM, and 2.5 μM in A2780 cells (**b**), 1.25 μM, and 2.5 μM in 786-O cells (**c**), 1.25-5 μM in KHOS cells (**d**)

and 2-8 μM in H1299 cells (**e**). Lipid ROS production was assayed after 12 h by flow cytometry using C11-BODIPY and cell viability was assayed after 36 h with CCK8. **f** Indicated DJ-1 KO MEFs with DJ-1 re-overexpression were treated with Sorafenib (20μM, 50 μM) (the left view) and RSL-3 (50-400 nM) (the right view) for 24 h, and cell viability was determined. **g** Indicated DJ-1 KO MEFs with DJ-1 re-overexpression were treated with ML210 (200-800 nM or 200-1600 nM). Lipid ROS production (the left view) was assayed after 12 h by flow cytometry using C11-BODIPY and cell viability (the right view) was assayed after 36 h with CCK8. Data shown represent mean ± SD from three independent experiments. Comparisons were made using the two-tailed, unpaired Student's t test. *: $p < 0.05$; **: $p < 0.01$; n.s.: no statistic difference.

Supplementary Figure 3

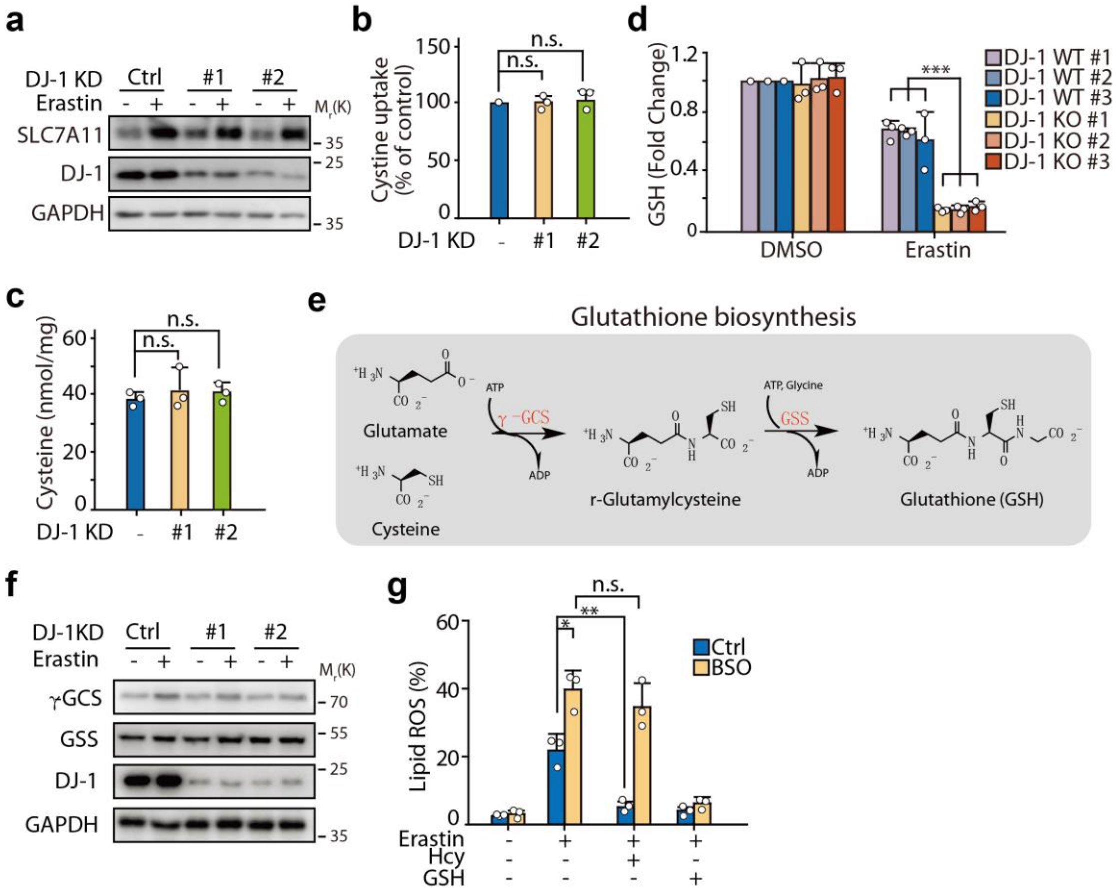

Supplmentary Figure 3. a Indicated DJ-1 knockdown H1299 cells were treated with Erastin (2 μM) for 12 h and subjected to western blot analysis of the indicated proteins in whole cell extracts. Independent experiments are repeated three times and representative data are shown. **b** Na^+-independent uptake of cystine in indicated H1299 cells are shown. **c** The levels of endocellular cysteine in indicated H1299 cells are shown. **d** Indicated DJ-1 KO H1299 cells were treated with Erastin (2 μM) for 6 h and intracellular GSH levels were examined. **e** The schematic representation of glutathione biosynthesis. Gamma-glutamylcysteine synthetase (γ-GCS) and glutathione synthetase (GSS) are two important rate-limiting enzymes of glutathione synthesis. **f** Indicated DJ-1 knockdown H1299 cells were treated with Erastin (2 μM) for 12 h and subjected to western blot analysis of the indicated proteins in whole cell

extracts. Independent experiments are repeated three times and representative data are shown. **g** H1299 cells were treated Erastin (2 μM) with BSO (pretreated 4 h, 100 μM) with or without GSH (0.5 mM) and Hcy (0.5 mM) for another 12 h, and lipid ROS production was assayed by flow cytometry using C11-BODIPY. Data shown represent mean ± SD from three independent experiments. Comparisons were made using the two-tailed, unpaired Student's t test. *: $p < 0.05$; **: $p < 0.01$; ***: $p < 0.001$; n.s.: no statistic difference.

Supplementary Figure 4

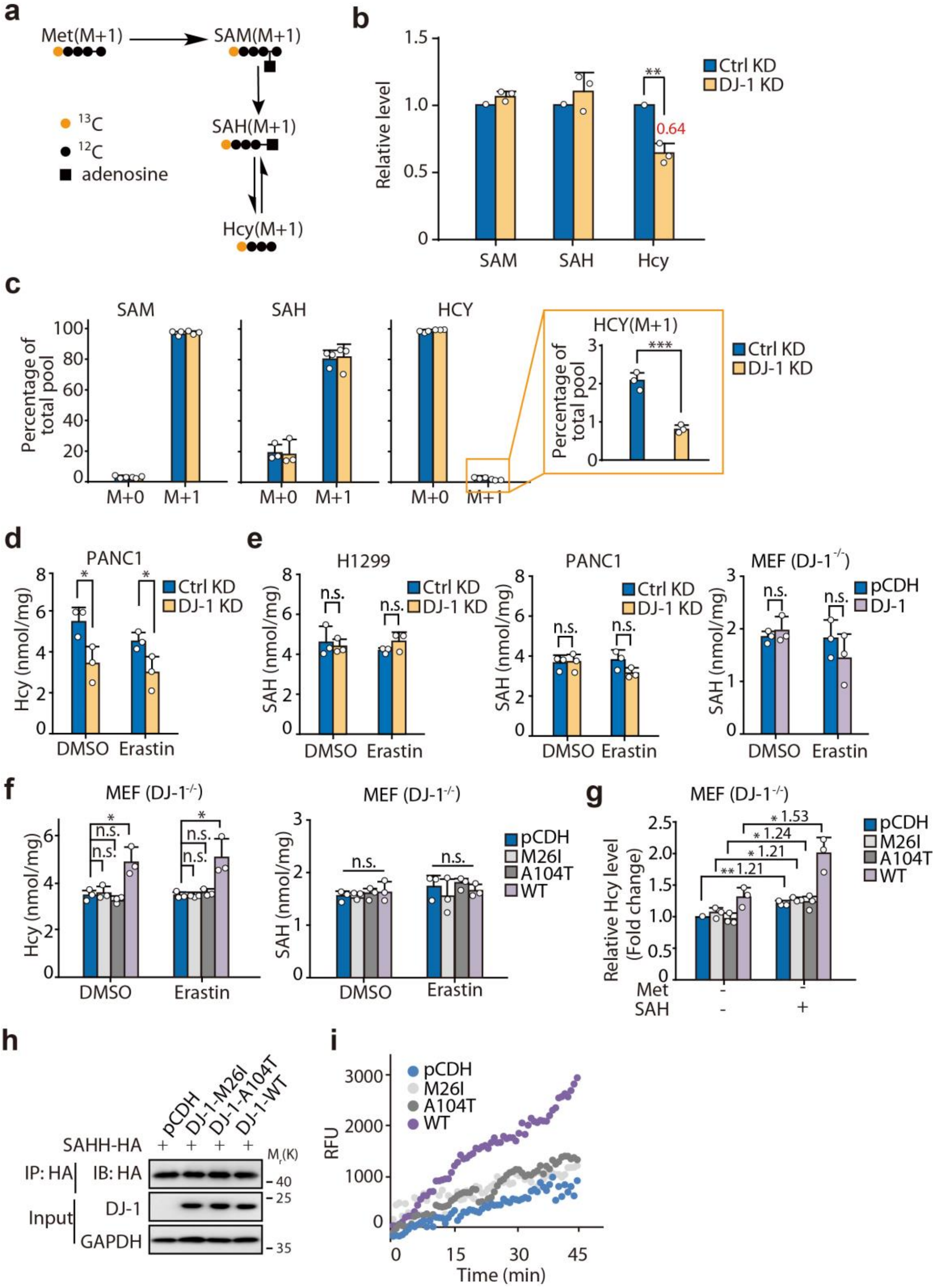

Supplementary Figure 4. a Outline of carbon flux in metabolic pathways leading from labeled ^{13}C-Met (yellow) to Hcy via transsulfuration. **b** Relative metabolite level (all isotopologues) determined by mass spectrometry. ^{13}C- Met was added only during the

4 h treatment phase. **c** Isotopologues of SAM, SAH, Hcy detected in the experiment outlined in (**b**). **d** PANC1 cells were infected with shRNAs for 72 h (#1 sequence of DJ-1 knockdown was used here), and the levels of endocellular Hcy were assayed after treated with Erastin (2 μM) for 12 h by ELISA assays. **e** ELISA assays for the levels of endocellular SAH after treated with Erastin for 12 h. The SAH levels in indicated H1299 cells (Erastin 2 μM, the left view), PANC1 cells (Erastin 2 μM, the middle view) and MEFs (Erastin 400 nM, the right view) are shown. **f, g** Indicated DJ-1 KO MEFs were transfected with different DJ-1 mutations. **f** The Hcy levels (the left view) and the SAH (the right view) levels in indicated MEFs treated with Erastin (400 nM) for 12 h are shown. **g** Indicated cells were deprived from Met for 24 h, followed by adding the extra SAH to the cells for 4 h, and Hcy levels we detected by ELISA. The relative Hcy levels are shown. **h, i** Indicated HEK293T cells with different DJ-1 mutations overexpression were further transfected with SAHH-HA plasmids. **h** Cell lysates were immunoprecipitated with anti-HA antibody, followed by immunoblotting with anti-HA antibody. Independent experiments are repeated three times and representative data are shown. **i** The activity of ectopic SAHH from indicated cells by immunoprecipitation was assayed as mentioned in methods. Independent experiments were repeated three times and representative data are shown. Wildtype DJ-1 was used here for positive control. Data shown represent mean ± SD from three independent experiments. Comparisons were made using the two-tailed, unpaired Student's t test. *: $p < 0.05$; **: $p < 0.01$; ***: $p < 0.001$; n.s.: no statistic difference.

Supplementary Figure 5

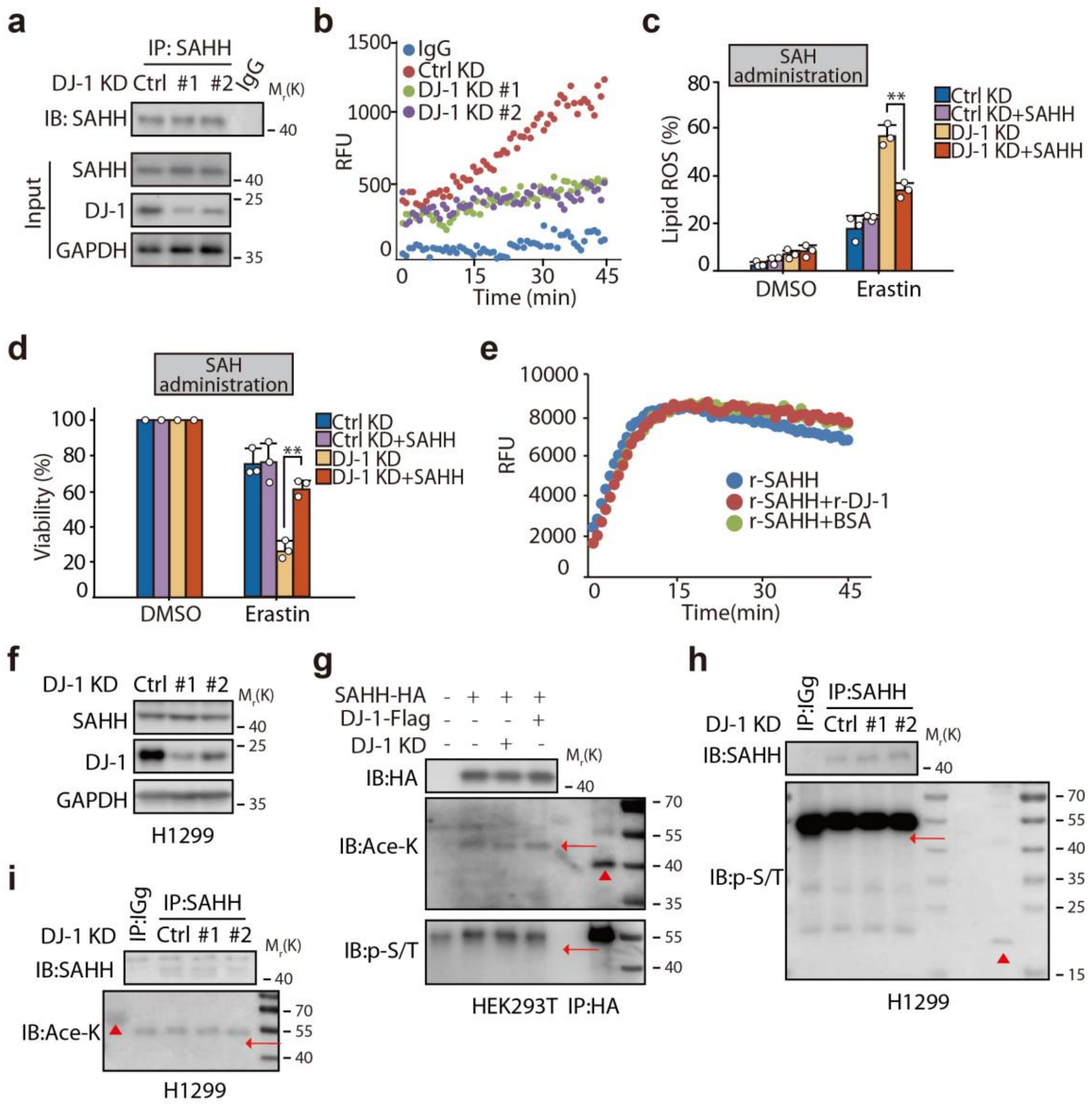

Supplementary Figure 5. a Cell lysates of indicated DJ-1 knockdown H1299 cells were immunoprecipitated with anti-SAHH antibody, followed by immunoblotting with anti-SAHH antibody. Independent experiments are repeated three times and representative data are shown. **b** The intracellular enzymatic activity of SAHH are shown. Independent experiments are repeated three times and representative data are shown. **c, d** Indicated DJ-1 knockdown H1299 cells were further transfected with SAHH-HA plasmids and treated with Erastin (2 μM) and SAH (0.5 mM) for 12 or 36 h, and lipid ROS production (**c**) and cell viability (**d**) were assayed. **e** The recombinant

SAHH was pre-incubated with recombinant DJ-1 or BSA for 1h and subsequently the activity was assayed as mentioned in methods. Independent experiments were repeated three times and representative data are shown. **f-i** DJ-1 did not influence the expression and post-translational modification of SAHH. Independent experiments are repeated three times and representative data are shown. **f** The protein level of SAHH in indicated DJ-1 knockdown H1299 cells was analyzed by western blot. The acetylation modification in exogenous SAHH (**g**) and in endogenous SAHH (**i**) were assayed in indicated cells; and the phosphorylation of SAHH was detected both in exogenous (**g**) and endogenous (**h**) SAHH. Red triangle representatives the positive signal which was used for quality control; red arrow indicates the position of the target signal. Data shown represent mean ± SD from three independent experiments. Comparisons were made using the two-tailed, unpaired Student's t test. **: $p < 0.01$.

Supplementary Figure 6

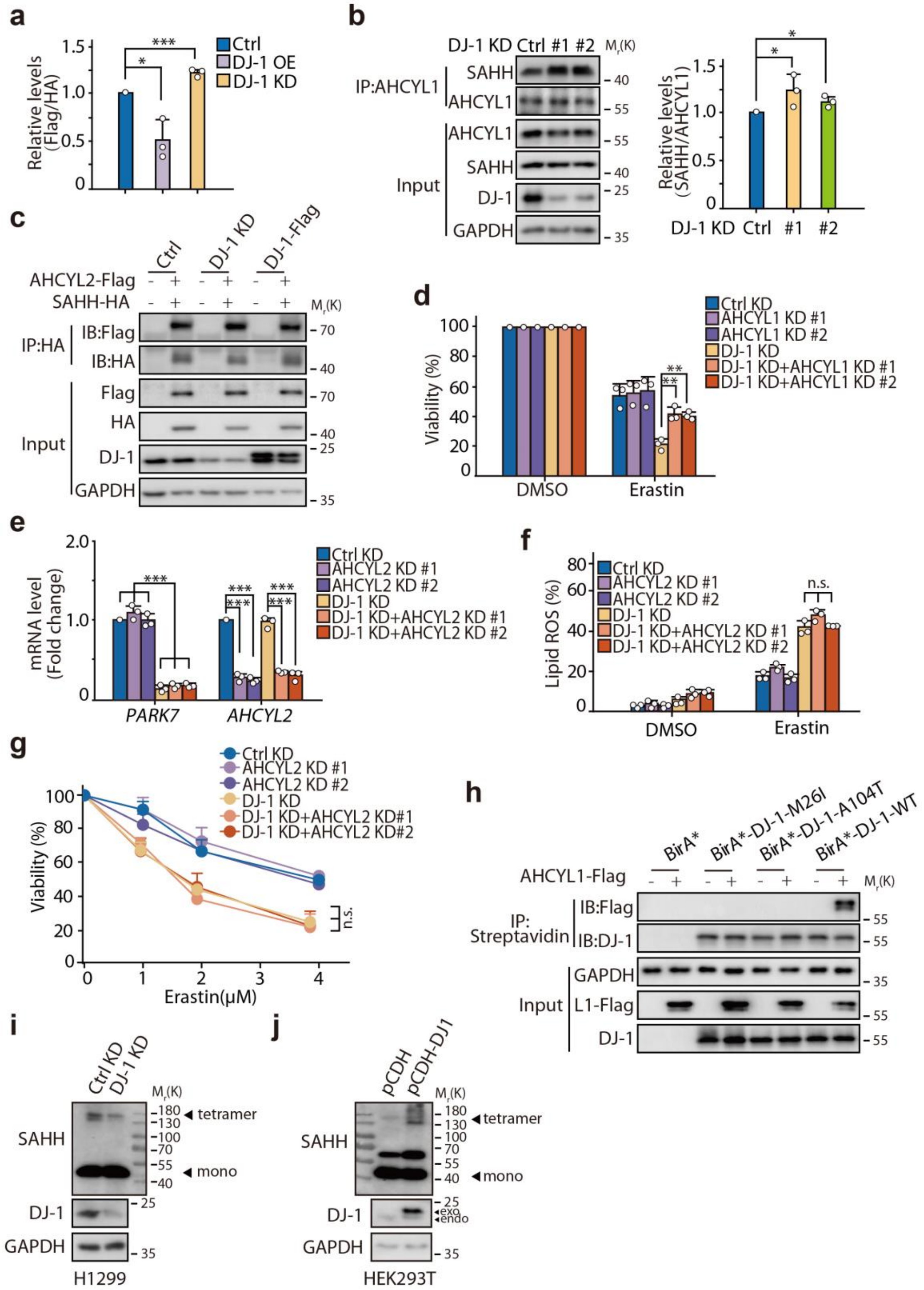

Supplementary Figure 6. a Quantitative analysis of the interaction between AHCYL1-SAHH under different DJ-1 level was shown. **b** The interaction between AHCYL1 and SAHH in indicated DJ-1 knockdown H1299 cells was analyzed, and the quantitative

analysis was shown (the right view). **c** The interaction between AHCYL2 and SAHH under different DJ-1 level was analyzed. Indicated HEK293T cells stably expressing AHCYL2 and SAHH were harvested for immunoprecipitation and subjected to immunoblotting with anti-Flag and anti-HA antibodies (#1 sequence of DJ-1 knockdown was used here). Independent experiments are repeated three times and representative data are shown. **d** Indicated DJ-1 knockdown (#1 sequence) and AHCYL1 knockdown H1299 cells were treated with Erastin (2 μM) for 36 h, and cell viability was assayed. **e** The mRNA expression of *PARK7* and *AHCYL2* in indicated H1299 cells was assayed by qRT-PCR (#1 sequence of DJ-1 knockdown was used here). The relative gene expression is normalized to β-actin and the error bar indicates the s.d. value from triplicates. **f** Indicated DJ-1 knockdown (#1 sequence) and AHCYL2 knockdown H1299 cells were treated with Erastin (2 μM) for 12 h, and lipid ROS production was assayed. **g** Cell viability was assayed in indicated DJ-1 knockdown H1299 cells treated for 36 h with Erastin (1-4 μM). **h** The interaction between AHCYL1 and mutant DJ-1 in BirA* system was analyzed. Wild type DJ-1 was used here for positive control. Independent experiments are repeated three times and representative data are shown. **i** DSS cross-linking assays of SAHH in indicated DJ-1 knockdown H1299 cells (#1 sequence of DJ-1 knockdown was used here). Independent experiments are repeated three times and representative data are shown. **j** DSS cross-linking assays of SAHH in HEK293T cells with DJ-1 overexpression. Independent experiments are repeated three times and representative data are shown. Data shown represent mean ± SD from three independent experiments. Comparisons were made

using the two-tailed, unpaired Student’s t test. *: $p < 0.05$; **: $p < 0.01$; ***: $p < 0.001$; n.s.: no statistic difference.

Supplementary Figure 7

a

shRNA	PE (mg/kg)	Tumor (g)	Inhibit (%)	T/C
Ctrl	-	1.19 ± 0.18	/	/
Ctrl	30	1.07 ± 0.29	9.62	0.84
DJ-1 KD	-	1.22 ± 0.30	-2.71	1.18
DJ-1 KD	30	0.65 ± 0.17	45.49	0.36

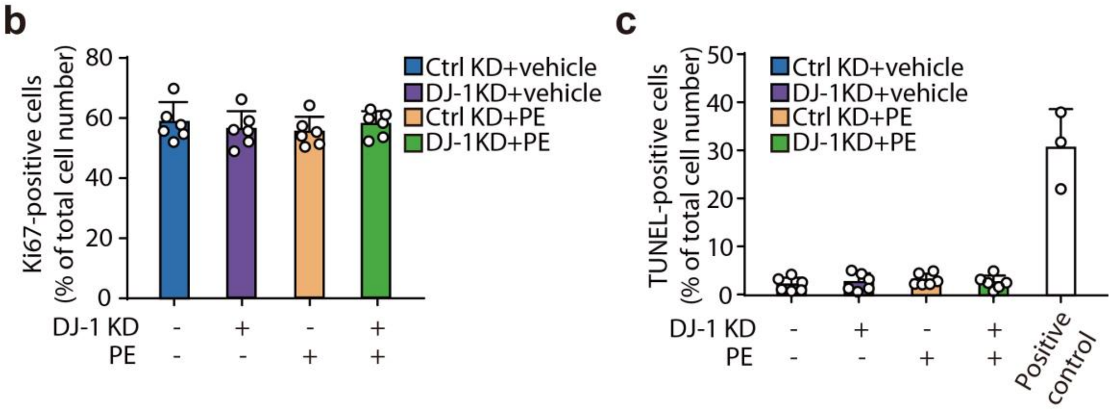

Supplementary Figure 7. a The mice were injected subcutaneously with indicated H1299 cells and treated with Piperazine Erastin (PE) (30 mg/kg, once every other day) through tail vein injection for 16 days. At the end of the experiment, the tumors were weighed, and the inhibition rate and T/C value (RTV of treatment/RTV of control) were calculated. n=6 biologically independent samples per group in experimental group. Data shown represent mean ± SEM. **b** Expression of Ki67 was detected in tumor tissues, and the Ki67-positive cells were counted. n=6 biologically independent samples per group in experimental group. Data shown represent mean ± SD. **c** TUNEL assay was performed, and the TUNEL-positive cells were counted. n=6 biologically independent samples per group in experimental group, n=3 biologically independent

samples in positive control group. Data shown represent mean ± SD.

Supplementary Table 1 . The unique proteins identified in the biotin-treated BirA*-DJ-1 cells by mass spectrometry					
prot_acc	prot_desc	prot_score	emPAI	prot_mass	no._spectra
sp|P36873|PP1G_HUMAN	Serine/threonine-protein phosphatase PP1-gamma catalytic subunit OS=Homo sapiens OX=9606 GN=PPP1CC PE=1 SV=1	411	0.38	37701	4
sp|Q13838|DX39B_HUMAN	Spliceosome RNA helicase DDX39B OS=Homo sapiens OX=9606 GN=DDX39B PE=1 SV=1	289	0.64	49416	8
sp|O43865|AHCYL_HUMAN	S-adenosylhomocysteine hydrolase-like protein OS=Homo sapiens OX=9606 GN=AHCYL PE=1 SV=1	189	0.09	67705	2

sp|P60033|CD81_HUMAN	CD81 antigen OS=Homo sapiens OX=9606 GN=CD81 PE=1 SV=1	188	0.25	26476	2
sp|Q96QD8|S38A2_HUMAN	Sodium-coupled neutral amino acid transporter 2 OS=Homo sapiens OX=9606 GN=SLC38A2 PE=1 SV=2	165	0.11	56332	2
sp|O14529|CUX2_HUMAN	Homeobox protein cut-like 2 OS=Homo sapiens OX=9606 GN=CUX2 PE=1 SV=4	159	0.02	162091	1
sp|Q9BYG3|MK67I_HUMAN	MKI67 FHA domain-interacting nucleolar phosphoprotein OS=Homo sapiens OX=9606 GN=NIFK PE=1 SV=1	155	0.09	34372	1

sp|P53367|ARFP1_HUMAN	Arfaptin-1 OS=Homo sapiens OX=9606 GN=ARFIP1 PE=1 SV=2	151	0.24	41770	3
sp|Q9UQL6|HDAC5_HUMAN	Histone deacetylase 5 OS=Homo sapiens OX=9606 GN=HDAC5 PE=1 SV=2	150	0.03	122700	1
sp|P50542|PEX5_HUMAN	Peroxisomal targeting signal 1 receptor OS=Homo sapiens OX=9606 GN=PEX5 PE=1 SV=3	143	0.04	71163	1
sp|Q96A65|EXOC4_HUMAN	Exocyst complex component 4 OS=Homo sapiens OX=9606 GN=EXOC4 PE=1 SV=1	130	0.06	111170	2

sp|Q86U38|NOP9_HUMAN	Nucleolar protein 9 OS=Homo sapiens OX=9606 GN=NOP9 PE=1 SV=1	122	0.04	70136	1
sp|Q9BW27|NUP85_HUMAN	Nuclear pore complex protein Nup85 OS=Homo sapiens OX=9606 GN=NUP85 PE=1 SV=1	121	0.13	75826	3
sp|O43592|XPOT_HUMAN	Exportin-T OS=Homo sapiens OX=9606 GN=XPOT PE=1 SV=2	115	0.06	111148	2
sp|O94832|MYO1D_HUMAN	Unconventional myosin-Id OS=Homo sapiens OX=9606 GN=MYO1D PE=1 SV=2	111	0.05	116927	2

sp|Q5SNT2|TM201_HUMAN	Transmembrane protein 201 OS=Homo sapiens OX=9606 GN=TMEM201 PE=1 SV=1	111	0.04	73444	1
sp|Q8NA72|POC5_HUMAN	Centrosomal protein POC5 OS=Homo sapiens OX=9606 GN=POC5 PE=1 SV=2	105	0.05	63711	1
sp|Q07021|C1QBP_HUMAN	Complement component 1 Q subcomponent-binding protein, mitochondrial OS=Homo sapiens OX=9606 GN=C1QBP PE=1 SV=1	97	0.1	31742	1
sp|Q96E39|RMX	RNA binding motif protein, X-linked-	96	0.07	42173	1

L1_HUMAN	like-1 OS=Homo sapiens OX=9606 GN=RBMXL1 PE=1 SV=1				
sp|P0DJJ0|SRG2C_HUMAN	SLIT-ROBO Rho GTPase-activating protein 2C OS=Homo sapiens OX=9606 GN=SRGAP2C PE=1 SV=1	95	0.06	54077	1
sp|P31350|RIR2_HUMAN	Ribonucleoside-diphosphate reductase subunit M2 OS=Homo sapiens OX=9606 GN=RRM2 PE=1 SV=1	93	0.07	45134	1
sp|Q9H8H3|MET7A_HUMAN	Methyltransferase-like protein 7A OS=Homo sapiens OX=9606	92	0.11	28814	1

	GN=METTL7A PE=1 SV=1				
sp|O95759|TBCD8_HUMAN	TBC1 domain family member 8 OS=Homo sapiens OX=9606 GN=TBC1D8 PE=1 SV=3	86	0.02	132177	1
sp|P0DOX7|IGK_HUMAN	Immunoglobulin kappa light chain OS=Homo sapiens OX=9606 PE=1 SV=1	82	0.13	23650	1
sp|Q9UKA4|AKA11_HUMAN	A-kinase anchor protein 11 OS=Homo sapiens OX=9606 GN=AKAP11 PE=1 SV=1	81	0.01	212946	1
sp|P78406|RAE1	mRNA export factor OS=Homo sapiens	80	0.08	41569	1

L_HUMAN	OX=9606 GN=RAE1 PE=1 SV=1				
sp|Q9H3S7|PTN23_HUMAN	Tyrosine-protein phosphatase non-receptor type 23 OS=Homo sapiens OX=9606 GN=PTPN23 PE=1 SV=1	79	0.02	179830	1
sp|Q14596|NBR1_HUMAN	Next to BRCA1 gene 1 protein OS=Homo sapiens OX=9606 GN=NBR1 PE=1 SV=3	79	0.03	108486	1
sp|P10768|ESTD_HUMAN	S-formylglutathione hydrolase OS=Homo sapiens OX=9606 GN=ESD PE=1 SV=2	79	0.1	31956	1
sp|P09972|ALDO	Fructose-bisphosphate aldolase C	76	0.08	39830	1

C_HUMAN	OS=Homo sapiens OX=9606 GN=ALDOC PE=1 SV=2				
sp|Q9H4A3|WNK1_HUMAN	Serine/threonine-protein kinase WNK1 OS=Homo sapiens OX=9606 GN=WNK1 PE=1 SV=2	76	0.01	251552	1
sp|P60059|SC61G_HUMAN	Protein transport protein Sec61 subunit gamma OS=Homo sapiens OX=9606 GN=SEC61G PE=1 SV=1	75	0.43	7793	1
sp|Q9NRG9|AAAS_HUMAN	Aladin OS=Homo sapiens OX=9606 GN=AAAS PE=1 SV=1	72	0.16	60392	3

sp|Q14166|TTL12_HUMAN	Tubulin--tyrosine ligase-like protein 12 OS=Homo sapiens OX=9606 GN=TTLL12 PE=1 SV=2	71	0.04	75154	1
sp|Q12996|CSTF3_HUMAN	Cleavage stimulation factor subunit 3 OS=Homo sapiens OX=9606 GN=CSTF3 PE=1 SV=1	67	0.04	83325	1
sp|P07199|CENPB_HUMAN	Major centromere autoantigen B OS=Homo sapiens OX=9606 GN=CENPB PE=1 SV=2	64	0.05	65531	1
sp|Q5HYK7|SH319_HUMAN	SH3 domain-containing protein 19 OS=Homo sapiens OX=9606	62	0.04	87042	1

	GN=SH3D19 PE=1 SV=2				
sp|Q8N3F8|MILK1_HUMAN	MICAL-like protein 1 OS=Homo sapiens OX=9606 GN=MICALL1 PE=1 SV=2	61	0.03	94352	1
sp|P25098|ARBK1_HUMAN	Beta-adrenergic receptor kinase 1 OS=Homo sapiens OX=9606 GN=GRK2 PE=1 SV=2	52	0.04	80321	1
sp|O00400|ACATN_HUMAN	Acetyl-coenzyme A transporter 1 OS=Homo sapiens OX=9606 GN=SLC33A1 PE=1 SV=1	52	0.1	61383	2
sp|P00387|NB5R	NADH-cytochrome b5 reductase 3 OS=Homo sapiens	51	0.19	34441	2

3_HUMAN	OX=9606 GN=CYB5R3 PE=1 SV=3				
sp|Q92879|CELF1_HUMAN	CUGBP Elav-like family member 1 OS=Homo sapiens OX=9606 GN=CELF1 PE=1 SV=2	48	0.06	52429	1
sp|P09429|HMGB1_HUMAN	High mobility group protein B1 OS=Homo sapiens OX=9606 GN=HMGB1 PE=1 SV=3	46	0.27	25049	2
sp|Q9UBB4|ATX10_HUMAN	Ataxin-10 OS=Homo sapiens OX=9606 GN=ATXN10 PE=1 SV=1	46	0.06	54196	1
sp|O00767|ACO	Acyl-CoA desaturase OS=Homo sapiens	44	0.08	41667	1

D_HUMAN	OX=9606 GN=SCD PE=1 SV=2				
sp|P84098|RL19_HUMAN	60S ribosomal protein L19 OS=Homo sapiens OX=9606 GN=RPL19 PE=1 SV=1	44	0.14	23565	1
sp|Q68E01|INT3_HUMAN	Integrator complex subunit 3 OS=Homo sapiens OX=9606 GN=INTS3 PE=1 SV=1	43	0.03	119533	1
sp|Q92890|UFD1_HUMAN	Ubiquitin recognition factor in ER-associated degradation protein 1 OS=Homo sapiens OX=9606 GN=UFD1 PE=1 SV=3	42	0.19	34763	2
sp|P29375|KDM	Lysine-specific demethylase 5A	41	0.03	194823	2

5A_HUMAN	OS=Homo sapiens OX=9606 GN=KDM5A PE=1 SV=3				
sp|P60228|EIF3E_HUMAN	Eukaryotic translation initiation factor 3 subunit E OS=Homo sapiens OX=9606 GN=EIF3E PE=1 SV=1	39	0.06	52587	1
sp|O00116|ADAS_HUMAN	Alkyldihydroxyacetonephosphate synthase, peroxisomal OS=Homo sapiens OX=9606 GN=AGPS PE=1 SV=1	38	0.09	73664	2
sp|A1L390|PKH	Pleckstrin homology domain-containing family G member 3	38	0.02	135242	1

G3_HUMAN	OS=Homo sapiens OX=9606 GN=PLEKHG3 PE=1 SV=1				
sp|O95140|MFN2_HUMAN	Mitofusin-2 OS=Homo sapiens OX=9606 GN=MFN2 PE=1 SV=3	37	0.07	87088	2
sp|Q92783|STAM1_HUMAN	Signal transducing adapter molecule 1 OS=Homo sapiens OX=9606 GN=STAM PE=1 SV=3	37	0.05	59541	1
sp|Q9H6H4|REEP4_HUMAN	Receptor expression-enhancing protein 4 OS=Homo sapiens OX=9606 GN=REEP4 PE=1 SV=1	36	0.11	29547	1

sp|A1L0T0|ILVBL_HUMAN	Acetolactate synthase-like protein OS=Homo sapiens OX=9606 GN=ILVBL PE=1 SV=2	36	0.05	68452	1
sp|Q13895|BYST_HUMAN	Bystin OS=Homo sapiens OX=9606 GN=BYSL PE=1 SV=3	36	0.06	49798	1
sp|Q13561|DCTN2_HUMAN	Dynactin subunit 2 OS=Homo sapiens OX=9606 GN=DCTN2 PE=1 SV=4	36	0.07	44318	1
sp|Q6P1N0|C2D1A_HUMAN	Coiled-coil and C2 domain-containing protein 1A OS=Homo sapiens OX=9606 GN=CC2D1A PE=1 SV=1	35	0.03	104397	1

sp|O15144|ARPC2_HUMAN	Actin-related protein 2/3 complex subunit 2 OS=Homo sapiens OX=9606 GN=ARPC2 PE=1 SV=1	35	0.09	34426	1
sp|P00395|COX1_HUMAN	Cytochrome c oxidase subunit 1 OS=Homo sapiens OX=9606 GN=MT-CO1 PE=1 SV=1	34	0.05	57060	1
sp|Q8IWV7|UBR1_HUMAN	E3 ubiquitin-protein ligase UBR1 OS=Homo sapiens OX=9606 GN=UBR1 PE=1 SV=1	34	0.02	203445	1
sp|P84103|SRSF3_HUMAN	Serine/arginine-rich splicing factor 3 OS=Homo sapiens OX=9606 GN=SRSF3 PE=1 SV=1	34	0.36	19546	2

sp|O95372|LYPA2_HUMAN	Acyl-protein thioesterase 2 OS=Homo sapiens OX=9606 GN=LYPLA2 PE=1 SV=1	34	0.13	25063	1
sp|Q86WB0|NIPA_HUMAN	Nuclear-interacting partner of ALK OS=Homo sapiens OX=9606 GN=ZC3HC1 PE=1 SV=1	33	0.06	56366	1
sp|Q9UKV5|AMFR_HUMAN	E3 ubiquitin-protein ligase AMFR OS=Homo sapiens OX=9606 GN=AMFR PE=1 SV=2	33	0.09	73747	2
sp|Q32P41|TRM5_HUMAN	tRNA (guanine(37)-N1)-methyltransferase OS=Homo sapiens	33	0.05	58551	1

	OX=9606 GN=TRMT5 PE=1 SV=2				
sp|P30837|AL1B1_HUMAN	Aldehyde dehydrogenase X, mitochondrial OS=Homo sapiens OX=9606 GN=ALDH1B1 PE=1 SV=3	33	0.05	57626	1
sp|Q8TC12|RDH11_HUMAN	Retinol dehydrogenase 11 OS=Homo sapiens OX=9606 GN=RDH11 PE=1 SV=2	32	0.09	35763	1
sp|Q9UNF1|MAGD2_HUMAN	Melanoma-associated antigen D2 OS=Homo sapiens OX=9606 GN=MAGED2 PE=1 SV=2	32	0.1	65085	2

sp|Q9UM47|NOTC3_HUMAN	Neurogenic locus notch homolog protein 3 OS=Homo sapiens OX=9606 GN=NOTCH3 PE=1 SV=2	32	0.01	256640	1
sp|O15056|SYNJ2_HUMAN	Synaptojanin-2 OS=Homo sapiens OX=9606 GN=SYNJ2 PE=1 SV=3	32	0.02	166575	1
sp|Q13144|EI2BE_HUMAN	Translation initiation factor eIF-2B subunit epsilon OS=Homo sapiens OX=9606 GN=EIF2B5 PE=1 SV=3	32	0.04	81071	1
sp|O00750|P3C2B_HUMAN	Phosphatidylinositol 4-phosphate 3-kinase C2 domain-containing subunit beta OS=Homo	32	0.02	186817	1

	sapiens OX=9606 GN=PIK3C2B PE=1 SV=2				
sp|Q13610|PWP1_HUMAN	Periodic tryptophan protein 1 homolog OS=Homo sapiens OX=9606 GN=PWP1 PE=1 SV=1	32	0.06	56363	1
sp|Q15417|CNN3_HUMAN	Calponin-3 OS=Homo sapiens OX=9606 GN=CNN3 PE=1 SV=1	31	0.09	36562	1
sp|Q96CS3|FAF2_HUMAN	FAS-associated factor 2 OS=Homo sapiens OX=9606 GN=FAF2 PE=1 SV=2	31	0.06	52933	1
sp|Q7Z434|MAV	Mitochondrial antiviral-signaling protein OS=Homo	30	0.05	57063	1

S_HUMAN	sapiens OX=9606 GN=MAVS PE=1 SV=2				
sp|Q15910|EZH2_HUMAN	Histone-lysine N-methyltransferase EZH2 OS=Homo sapiens OX=9606 GN=EZH2 PE=1 SV=2	29	0.04	87247	1
sp|O94966|UBP19_HUMAN	Ubiquitin carboxyl-terminal hydrolase 19 OS=Homo sapiens OX=9606 GN=USP19 PE=1 SV=2	29	0.02	147441	1
sp|Q8N8A6|DDX51_HUMAN	ATP-dependent RNA helicase DDX51 OS=Homo sapiens OX=9606 GN=DDX51 PE=1 SV=3	29	0.04	72983	1

sp|Q92973|TNPO1_HUMAN	Transportin-1 OS=Homo sapiens OX=9606 GN=TNPO1 PE=1 SV=2	28	0.03	103771	1
sp|Q9NUU7|DD19A_HUMAN	ATP-dependent RNA helicase DDX19A OS=Homo sapiens OX=9606 GN=DDX19A PE=1 SV=1	28	0.06	54397	1
sp|Q9H1H9|KI13A_HUMAN	Kinesin-like protein KIF13A OS=Homo sapiens OX=9606 GN=KIF13A PE=1 SV=2	27	0.02	203836	1
sp|Q9HAW4|CLSPN_HUMAN	Claspin OS=Homo sapiens OX=9606 GN=CLSPN PE=1 SV=3	27	0.02	151629	1
sp|P49589|SYC	Cysteine--tRNA ligase, cytoplasmic	27	0.04	86103	1

C_HUMAN	OS=Homo sapiens OX=9606 GN=CARS PE=1 SV=3				
sp|Q5T5P2|SKT_HUMAN	Sickle tail protein homolog OS=Homo sapiens OX=9606 GN=KIAA1217 PE=1 SV=2	27	0.01	214610	1
sp|P35613|BASI_HUMAN	Basigin OS=Homo sapiens OX=9606 GN=BSG PE=1 SV=2	26	0.07	42573	1
sp|Q9NRW7|VPS45_HUMAN	Vacuolar protein sorting-associated protein 45 OS=Homo sapiens OX=9606 GN=VPS45 PE=1 SV=1	26	0.05	65435	1
sp|Q5TAX3|TUT4	Terminal uridylyltransferase 4	26	0.03	188014	2

_HUMAN	OS=Homo sapiens OX=9606 GN=TUT4 PE=1 SV=3				
sp|O15360|FANCA_HUMAN	Fanconi anemia group A protein OS=Homo sapiens OX=9606 GN=FANCA PE=1 SV=2	26	0.02	164838	1
sp|Q9P2D3|HTR5B_HUMAN	HEAT repeat-containing protein 5B OS=Homo sapiens OX=9606 GN=HEATR5B PE=1 SV=2	25	0.01	226839	1
sp|Q9NQW6|ANLN_HUMAN	Anillin OS=Homo sapiens OX=9606 GN=ANLN PE=1 SV=2	25	0.02	125490	1
sp|O95235|KI20A	Kinesin-like protein KIF20A OS=Homo sapiens OX=9606	25	0.03	101242	1

_HUMAN	GN=KIF20A PE=1 SV=1				
sp|Q9UI12|VATH_HUMAN	V-type proton ATPase subunit H OS=Homo sapiens OX=9606 GN=ATP6V1H PE=1 SV=1	25	0.06	56417	1
sp|Q86UK7|ZN598_HUMAN	E3 ubiquitin-protein ligase ZNF598 OS=Homo sapiens OX=9606 GN=ZNF598 PE=1 SV=1	25	0.03	100400	1
sp|Q5JVF3|PCID2_HUMAN	PCI domain-containing protein 2 OS=Homo sapiens OX=9606 GN=PCID2 PE=1 SV=2	24	0.07	46627	1
sp|Q16644|MAP	MAP kinase-activated protein	24	0.07	43473	1

K3_HUMAN	kinase 3 OS=Homo sapiens OX=9606 GN=MAPKAPK3 PE=1 SV=1				
sp|P23921|RIR1_HUMAN	Ribonucleoside-diphosphate reductase large subunit OS=Homo sapiens OX=9606 GN=RRM1 PE=1 SV=1	23	0.03	90925	1
sp|Q96SN8|CK5P2_HUMAN	CDK5 regulatory subunit-associated protein 2 OS=Homo sapiens OX=9606 GN=CDK5RAP2 PE=1 SV=5	23	0.01	216444	1
sp|P53350|PLK1_HUMAN	Serine/threonine-protein kinase PLK1 OS=Homo sapiens OX=9606 GN=PLK1 PE=1 SV=1	23	0.05	68953	1

sp|Q9UBD5|ORC3_HUMAN	Origin recognition complex subunit 3 OS=Homo sapiens OX=9606 GN=ORC3 PE=1 SV=1	22	0.04	83285	1
sp|Q96N66|MBOA7_HUMAN	Lysophospholipid acyltransferase 7 OS=Homo sapiens OX=9606 GN=MBOAT7 PE=1 SV=2	22	0.06	53415	1
sp|Q8N228|SCML4_HUMAN	Sex comb on midleg-like protein 4 OS=Homo sapiens OX=9606 GN=SCML4 PE=1 SV=2	22	0.07	45507	1
sp|Q9UPV0|CE164_HUMAN	Centrosomal protein of 164 kDa OS=Homo sapiens OX=9606	20	0.02	164727	1

	GN=CEP164 PE=1 SV=3				
sp|O43505|B4GA1_HUMAN	Beta-1,4-glucuronyltransferase 1 OS=Homo sapiens OX=9606 GN=B4GAT1 PE=1 SV=1	20	0.07	47545	1
sp|Q8NBM4|UBAC2_HUMAN	Ubiquitin-associated domain-containing protein 2 OS=Homo sapiens OX=9606 GN=UBAC2 PE=1 SV=1	19	0.08	39337	1
sp|Q86W42|THOC6_HUMAN	THO complex subunit 6 homolog OS=Homo sapiens OX=9606 GN=THOC6 PE=1 SV=1	19	0.08	38081	1
sp|O95251|KAT7	Histone acetyltransferase	19	0.04	71282	1

_HUMAN	KAT7 OS=Homo sapiens OX=9606 GN=KAT7 PE=1 SV=1				
sp|Q9Y3A4|RRP7A_HUMAN	Ribosomal RNA-processing protein 7 homolog A OS=Homo sapiens OX=9606 GN=RRP7A PE=1 SV=2	18	0.1	32485	1
sp|Q8IWR0|Z3H7A_HUMAN	Zinc finger CCCH domain-containing protein 7A OS=Homo sapiens OX=9606 GN=ZC3H7A PE=1 SV=1	17	0.03	112348	1
sp|P63241|IF5A1_HUMAN	Eukaryotic translation initiation factor 5A-1 OS=Homo sapiens	16	0.19	17049	1

	OX=9606 GN=EIF5A PE=1 SV=2				
sp|Q9UGP4|LIMD1_HUMAN	LIM domain-containing protein 1 OS=Homo sapiens OX=9606 GN=LIMD1 PE=1 SV=1	16	0.04	73855	1
sp|P31689|DNJA1_HUMAN	DnaJ homolog subfamily A member 1 OS=Homo sapiens OX=9606 GN=DNAJA1 PE=1 SV=2	16	0.07	45581	1
sp|P56182|RRP1_HUMAN	Ribosomal RNA processing protein 1 homolog A OS=Homo sapiens OX=9606 GN=RRP1 PE=1 SV=1	16	0.06	53035	1

sp|Q9H1I8|ASCC2_HUMAN	Activating signal cointegrator 1 complex subunit 2 OS=Homo sapiens OX=9606 GN=ASCC2 PE=1 SV=3	15	0.04	87048	1
sp|O15173|PGRC2_HUMAN	Membrane-associated progesterone receptor component 2 OS=Homo sapiens OX=9606 GN=PGRMC2 PE=1 SV=1	15	0.13	23861	1
sp|Q8NHH9|ATLA2_HUMAN	Atlastin-2 OS=Homo sapiens OX=9606 GN=ATL2 PE=1 SV=2	15	0.05	66814	1
sp|Q96F86|EDC3	Enhancer of mRNA-decapping protein 3 OS=Homo sapiens	14	0.06	56784	1

_HUMAN	OX=9606 GN=EDC3 PE=1 SV=1				
sp|P04196|HRG_HUMAN	Histidine-rich glycoprotein OS=Homo sapiens OX=9606 GN=HRG PE=1 SV=1	14	0.05	60510	1
sp|Q8WZA2|RPGF4_HUMAN	Rap guanine nucleotide exchange factor 4 OS=Homo sapiens OX=9606 GN=RAPGEF4 PE=1 SV=1	14	0.03	116474	1
sp|O95382|M3K6_HUMAN	Mitogen-activated protein kinase kinase kinase 6 OS=Homo sapiens OX=9606 GN=MAP3K6 PE=1 SV=3	13	0.02	144217	1
sp|Q96M95|CCD4	Coiled-coil domain-containing protein 42	13	0.08	38052	1

2_HUMAN	OS=Homo sapiens OX=9606 GN=CCDC42 PE=1 SV=2				

Supplementary Table 2. The prime used to construct the plasmids

Plasmid	Forward Prime	Reverse Prime
shDJ-1#1	CCGGACTCTGAGAATCGTGTGGAAACTCGAGTTTCCACACGATTCTCAGAGTTTTTG	AATTCAAAAAACTCTGAGAATCGTGTGGAAACTCGAGTTTCCACACGATTCTCAGAGT
shDJ-1#2	CCGGTTCTACCAGGAGGTAATCTGGCTCGAGCCAGATTACCTCCTGGTAGAATTTTTG	AATTCAAAAATTCTACCAGGAGGTAATCTGGCTCGAGCCAGATTACCTCCTGGTAGAA
shNRF2	CCGGCCGGCATTTCACTAAACACAACTCGAGTTGTGTTTAGTGAAATGCCGGTTTTTG	AATTCAAAAACCGGCATTTCACTAAACACAACTCGAGTTGTGTTTAGTGAAATGCCGG
shSAHH#1	CCGGATGCCATTGTGTGTAACATTGCTCGAGCAATGTTACACACAATGGCATTTTTTTG	AATTCAAAAAATGCCATTGTGTGTAACATTGCTCGAGCAATGTTACACACAATGGCATT

shSAHH#2	CCGGGTCAGGAGGGCAACATCTTTGCTCGAGCAAAGATGTTGCCCTCCTGACTTTTTG	AATTCAAAAAGTCAGGAGGGCAACATCTTTGCTCGAGCAAAGATGTTGCCCTCCTGAC
shAHCYL1#1	CCGGGCACTGATAGAACTCTATAATCTCGAGATTATAGAGTTCTATCAGTGCTTTTTG	AATTCAAAAAGCACTGATAGAACTCTATAATCTCGAGATTATAGAGTTCTATCAGTGC
shAHCYL1#2	CCGGCAGCAGCAATTTCTGTGTGAACTCGAGTTCACACAGAAATTGCTGCTGTTTTTG	AATTCAAAAACAGCAGCAATTTCTGTGTGAACTCGAGTTCACACAGAAATTGCTGCTG
shAHCYL2#1	CCGGGCTCTAGCAGAAAGTGGATTTCTCGAGAAATCCACTTTCTGCTAGAGCTTTTTG	AATTCAAAAAGCTCTAGCAGAAAGTGGATTTCTCGAGAAATCCACTTTCTGCTAGAGC
shAHCYL2#2	CCGGGCAGAGTTTGGACGAAGAGAACTCGAGTTCTCTTCGTCCAAACTCTGCTTTTTG	AATTCAAAAAGCAGAGTTTGGACGAAGAGAACTCGAGTTCTCTTCGTCCAAACTCTGC
pCDH-SAHH-HA	CGGAATTCACCATGTCTGACAAACTGCCCTA	CGGCGGCCGCTCAGGCGTAGTCGGGGACGTCGTAGGGGTAGTAGCGGTAGTGATCCG
pCDH-DJ-1-flag	CGGAATTCACCATGGATTACAAGGATGACGACGATAAGATGGCTTCCAAAAGAGCTC	CGGGATCCCTAGTCTTTAAGAACAAGTG

pCDH-DJ1 M26I	GGTCATCCCTGTAGATGTCATCAGGCGAGCTGGGATTAAGG	CCTTAATCCCAGCTCGCCTGATGACATCTACAGGGATGACC
pCDH-DJ1-E64D	CCTTGAAGATGCAAAAAAAGACGGACCATATGATGTGGTGG	CCACCACATCATATGGTCCGTCTTTTTTTGCATCTTCAAGG
pCDH-DJ1-R98Q	ACTGAAGGAGCAGGAAAACCAGAAGGGCCTGATAGCCGCCA	TGGCGGCTATCAGGCCCTTCTGGTTTTCCTGCTCCTTCAGT
pCDH-DJ1-A104T	CCGGAAGGGCCTGATAGCCACCATCTGTGCAGGTCCTACTG	CAGTAGGACCTGCACAGATGGTGGCTATCAGGCCCTTCCGG
pCDH-DJ1-D149A	TGAGAATCGTGTGGAAAAAGCCGGCCTGATTCTTACAAGCC	GGCTTGTAAGAATCAGGCCGGCTTTTTCCACACGATTCTCA
pCDH-DJ1-G150S	GAATCGTGTGGAAAAAGACTCCCTGATTCTTACAAGCCGGG	CCCGGCTTGTAAGAATCAGGGAGTCTTTTTCCACACGATTC
pCDH-DJ1-E163K	GGGGCCTGGGACCAGCTTCAAGTTTGCGCTTGCAATTGTTG	CAACAATTGCAAGCGCAAACTTGAAGCTGGTCCCAGGCCCC

pCDH-DJ1-L166P	GACCAGCTTCGAGTTTGCGCCTGCAATTGTTGAAGCCCTGA	TCAGGGCTTCAACAATTGCAGGCGCAAACTCGAAGCTGGTC
pCDH-DJ1-A171S	TGCGCTTGCAATTGTTGAATCCCTGAATGGCAAGGAGGTGG	CCACCTCCTTGCCATTCAGGGATTCAACAATTGCAAGCGCA
pCDH-AHCYL1-Flag	CGGGATCCACCATGTCGATGCCTGACGCGAT	CGGCGGCCGCTTAGTATCTGTAATAATTAG
pCDH-AHCYL2-Flag	CGGCTAGCACCATGTCGGTGCAGGTTGTGTC	CGGGATCCTCAATACCTGTAGTAATTAG

Supplementary Table 3. The sequences of the primers for the quantitative RT-PCR

Gene	Forward Prime	Reverse Prime
β-ACTIN	CACCATTGGCAATGAGCGGTTC	AGGTCTTTGCGGATGTCCACGT
PARK7	GTAGCCGTGATGTGGTCATTT	CTGTGCGCCCAGATTACCT
PTGS2	CTGGCGCTCAGCCATACAG	CGCACTTATACTGGTCAAATCCC

NRF2	CACATCCAGTCAGAAACCAGTGG	GGAATGTCTGCGCCAAAAGCTG
HMOX1	CCAGGCAGAGAATGCTGAGTTC	AAGACTGGGCTCTCCTTGTTGC
NQO1	GAAGAGCACTGATCGTACTGGC	GGATACTGAAAGTTCGCAGGG
FTH1	TCCTACGTTTACCTGTCCATGT	GTTTGTGCAGTTCCAGTAGTGA
FTL	TACGAGCGTCTCCTGAAGATGC	GGTTCAGCTTTTTCTCCAGGGC
GCLM	TGAACTGGGGCCGCCTGCGG	CAATGAACAGTTTTGCAGAA
CBS	GATTATCGAGCCGACATCCG	GTCCTCACAATCTCAGCCC
BHMT	GACCTTCACCTTCTATGCGAG	AGCTTCATTGACTTCCTGCC
MAT1A	CCCTGAAGGAGCAAGTCATC	AATCTTACGGCCAGTGACAC
MAT2A	ATGAACGGACAGCTCAACGG	CCAGCAAGAAGGATCATTCCAG
AHCY	GCACTTTGAGCAGATGAAGG	GCCCATTCTTCAACCGATACC
MS	ATCTCATCTGGAATAAAGACCCTG	TTCACAAGGGCATACTCAAGG
CTH	GGCCTGGTGTCTGTTAATTGT	GCCATTCCGTTTTTGAAATGCT
AHCYL2	TTCAACAAACGTCCCACCAAA	CCTGGGCGATGTCTCATCA
