# Supplementary material for: DJ-1 suppresses ferroptosis through preserving the activity of S-adenosyl homocysteine hydrolase
Source: Nat Commun. 2020 Mar 6;11:1251. doi: 10.1038/s41467-020-15109-y (PMC7060199; doi:10.1038/s41467-020-15109-y)
Supplement: Supplementary file 3 — Reporting Summary [file 41467_2020_15109_MOESM3_ESM.pdf]

nature research

Corresponding author(s): Meidan Ying

Last updated by author(s): Jan 30, 2020

Reporting Summary

Nature Research wishes to improve the reproducibility of the work that we publish. This form provides structure for consistency and transparency in reporting. For further information on Nature Research policies, see Authors & Referees and the Editorial Policy Checklist .

Statistics

For all statistical analyses, confirm that the following items are present in the figure legend, table legend, main text, or Methods section.

n/a	Confirmed	
[ ]	[x]	The exact sample size (*n*) for each experimental group/condition, given as a discrete number and unit of measurement
[ ]	[x]	A statement on whether measurements were taken from distinct samples or whether the same sample was measured repeatedly
[ ]	[x]	The statistical test(s) used AND whether they are one- or two-sided<br>*Only common tests should be described solely by name; describe more complex techniques in the Methods section.*
[x]	[ ]	A description of all covariates tested
[x]	[ ]	A description of any assumptions or corrections, such as tests of normality and adjustment for multiple comparisons
[ ]	[x]	A full description of the statistical parameters including central tendency (e.g. means) or other basic estimates (e.g. regression coefficient) AND variation (e.g. standard deviation) or associated estimates of uncertainty (e.g. confidence intervals)
[ ]	[x]	For null hypothesis testing, the test statistic (e.g. *F*, *t*, *r*) with confidence intervals, effect sizes, degrees of freedom and *P* value noted<br>*Give P values as exact values whenever suitable.*
[x]	[ ]	For Bayesian analysis, information on the choice of priors and Markov chain Monte Carlo settings
[x]	[ ]	For hierarchical and complex designs, identification of the appropriate level for tests and full reporting of outcomes
[x]	[ ]	Estimates of effect sizes (e.g. Cohen's *d*, Pearson's *r*), indicating how they were calculated

Our web collection on statistics for biologists contains articles on many of the points above.

Software and code

Policy information about availability of computer code

Data collection	1. Western Blot signal was recorded using a AI600 Imager (GE Healthcare Life Sciences).<br>2. Nano-LC-MS/MS data were acquired in Xcalibur 2.2 operation software (Thermo Fisher Scientific).<br>3. Flow cytometry data were collected using FACSuite flow cytometer (BD Biosciences).<br>4. EM Imaging data was collected using a transmission electron microscope software (Philips Electronic Instruments, Mahwah, USA).<br>5. Real-time PCR data was monitored using QuantStudio 6 Design and Analysis Software Version 2.3 (ThermoFisher).<br>6. Immunofluorescence Imaging data was collected using a Leica microscope software Leica Application Suite Version 4.3.0.<br>7. Fluorescent signal of SAHH activity assay was collected using Molecular Devices SpectraMax M5 plate reader software SoftMax Pro 6.3.<br>8. Metabolite analysis and quantitation were performed by the software Xcalibur 3.0.63 (Thermo Fisher Scientific).<br>9. The ELISA data analysis was collected using Molecular Devices SpectraMax M5 plate reader software SoftMax Pro 6.3.
Data analysis	1. The flow cytometry data were analyzed using FlowJo Version 7.6 software.<br>2. All statistic analysis was conducted by Prism 5.0C (Graphpad Software) software (described in methods section)

For manuscripts utilizing custom algorithms or software that are central to the research but not yet described in published literature, software must be made available to editors/reviewers. We strongly encourage code deposition in a community repository (e.g. GitHub). See the Nature Research guidelines for submitting code & software for further information.

Data

Policy information about availability of data

All manuscripts must include a data availability statement. This statement should provide the following information, where applicable:

- Accession codes, unique identifiers, or web links for publicly available datasets
- A list of figures that have associated raw data
- A description of any restrictions on data availability

1.The potential interaction proteins of DJ-1 based on BioID assay can be found in supplementary table (table S1)

2. All the plasmids used in this manuscript including the ones in BioID assay will be available from the corresponding author upon reasonable request.
3. All the raw data of the WB was provided as supplementary dataset.
4. All original data for charts were provided as supplementary dataset.

Field-specific reporting

Please select the one below that is the best fit for your research. If you are not sure, read the appropriate sections before making your selection.

[x] Life sciences [ ] Behavioural & social sciences [ ] Ecological, evolutionary & environmental sciences

For a reference copy of the document with all sections, see nature.com/documents/nr-reporting-summary-flat.pdf

Life sciences study design

All studies must disclose on these points even when the disclosure is negative.

Sample size	No statistical calculations were performed to determine sample size.
Data exclusions	No data exclusion.
Replication	All the experiments mentioned in manuscript were performed at least three times independently. All attempts at replication were successful.
Randomization	For the animal study, mice were randomly allocated into different groups. Mice employed were the same sex, age-matched and subjected to the same procedures.
Blinding	For the animal study, we don't show the group information in the cage labels and two experts were conducted. One is in charge of the administration and the other one is response for the data collection. The data collection person don't know the groups information until the end of the experiment. Thus, the investigators were blinded to group allocation during data collection and were not blinded to data analysis.

Reporting for specific materials, systems and methods

We require information from authors about some types of materials, experimental systems and methods used in many studies. Here, indicate whether each material, system or method listed is relevant to your study. If you are not sure if a list item applies to your research, read the appropriate section before selecting a response.

Materials & experimental systems

n/a	Involved in the study	
[ ]	[x]	Antibodies
[ ]	[x]	Eukaryotic cell lines
[x]	[ ]	Palaeontology
[ ]	[x]	Animals and other organisms
[x]	[ ]	Human research participants
[x]	[ ]	Clinical data

Methods

n/a	Involved in the study	
[x]	[ ]	ChIP-seq
[ ]	[x]	Flow cytometry
[x]	[ ]	MRI-based neuroimaging

Antibodies

Antibodies used	The antibodies to DJ-1 (#5933), p-p38 (#9216), p38 (#8690), AHCYL1 (#94248), SLC7A11 (#12691), Acetylated-Lysine (#9441), Phospho-(Ser/Thr) Phe (#9631) were obtained from Cell Signaling Technology. The primary antibody against Nrf2 (sc13032), g-GCS (sc390811), GSS (sc166882), SAHH (sc271389), Ki67 (sc15402) were obtained from Santa Cruz Biotechnology. The primary antibody against GAPDH (db106), HA (db2603) and Flag(db7002) were obtained from Diagnostic Biosystems. (This information is also available in the Methods section.)
Validation	These antibodies were validated by the vendor and used according to the vendor's instructions. The bands observed in WB can be further confirmed by the calculated molecular weight. Moreover, the same batch of most antibodies have been used in published papers.

Eukaryotic cell lines

Policy information about cell lines

Cell line source(s)	The H1299, A549, PANC1, H292, H838, 786-O, KHOS, A2780 and HEK293T cell lines were purchased from the Shanghai Institute of Biochemistry and Cell Biology (Shanghai, China). The MEF DJ-1 KO cells were generated from day 13.5 embryos of DJ-1 KO mice (B6.Cg-Park7tm1Shn, #006577, The Jackson Labratory) according to standard procedures.

Authentication	All cancer cell lines used in our manuscript were authenticated by STR profiling.
Mycoplasma contamination	All the cell lines used in our manuscript were monitored Mycoplasma contamination every half a year. And all the Mycoplasma test were negative.
Commonly misidentified lines (See ICLAC register)	No commonly misidentified cell lines were used in the study

Animals and other organisms

Policy information about studies involving animals; ARRIVE guidelines recommended for reporting animal research

Laboratory animals	BALB/c female athymic nude mice (5 weeks, National Rodent Laboratory Animal Resource, Shanghai, China). The mice were housed in individually ventilated cages (IVC) and supplied with sterilized food, water, and bedding. The mice were maintained at environmental temperature and humidity ranges of 21 to 26 °C and 50% to 70%, respectively.
Wild animals	No wild animals were used in the study
Field-collected samples	No field collected samples were used in the study
Ethics oversight	Maintenance and experimental procedures for the mice studies were approved by Zhejiang University's IACUC (P-IACUC-004)

Note that full information on the approval of the study protocol must also be provided in the manuscript.

Flow Cytometry

Plots

Confirm that:

- [x] The axis labels state the marker and fluorochrome used (e.g. CD4-FITC).
- [x] The axis scales are clearly visible. Include numbers along axes only for bottom left plot of group (a 'group' is an analysis of identical markers).
- [x] All plots are contour plots with outliers or pseudocolor plots.
- [x] A numerical value for number of cells or percentage (with statistics) is provided.

Methodology

Sample preparation	The day before the experiment, 150,000 cells/well were seeded in 6-well dishes. The day of the experiment, cells were treated with test compounds for the indicated times, harvested by trypsinization, resuspended in 500 μL PBS containing 2 μM C11-BODIPY(581/591) (#D3861, Invitrogen), and incubated for 30 min at 37°C in a tissue culture incubator. Cells were then resuspended in 500 μL of fresh PBS, strained through a 40 μM cell strainer, and analyzed using a flow cytometer.
Instrument	All the samples were analyzed using BD FACSuite™ Software (BD Biosciences).
Software	Flow data was analyzed in FlowJo Version 7.6
Cell population abundance	A minimum of 10,000 post-staining cells were analyzed for each condition.
Gating strategy	Use FSC/SSC gate to exclude the cell debris. We also used cells without C11-BODIPY staining as the negative control for gating the lipid-ROS positive cells.

- [x] Tick this box to confirm that a figure exemplifying the gating strategy is provided in the Supplementary Information.
