# Supplementary material for: DJ-1 suppresses ferroptosis through preserving the activity of S-adenosyl homocysteine hydrolase
Source: Nat Commun. 2020 Mar 6;11:1251. doi: 10.1038/s41467-020-15109-y (PMC7060199; doi:10.1038/s41467-020-15109-y)
Supplement: Supplementary file 4 — Source Data [file 41467_2020_15109_MOESM4_ESM.pdf]

DJ-1 Suppresses Ferroptosis Through Preserving The Activity of S-adenosyl Homocysteine Hydrolase

Cao et al.

Raw data of western blot

Figure 1a

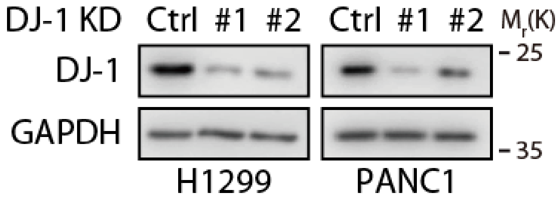

Raw data of western blot:

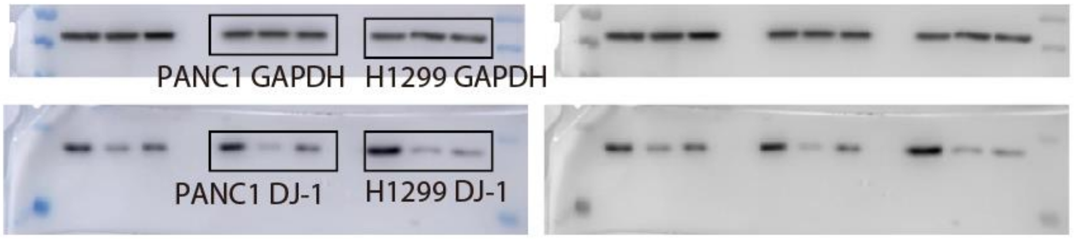

Figure 2a

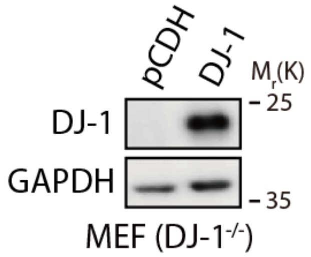

Raw data of western blot:

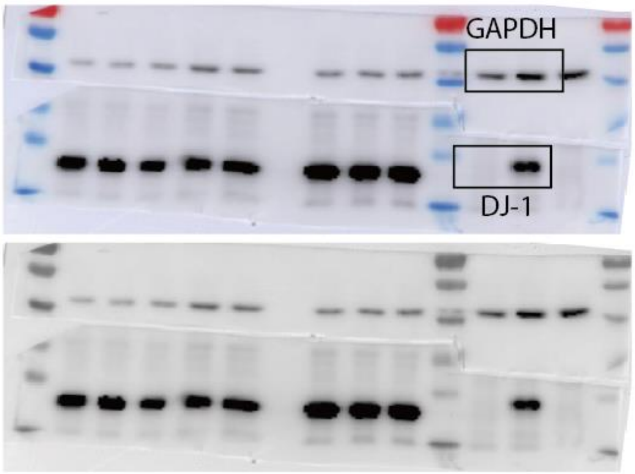

Figure 2e

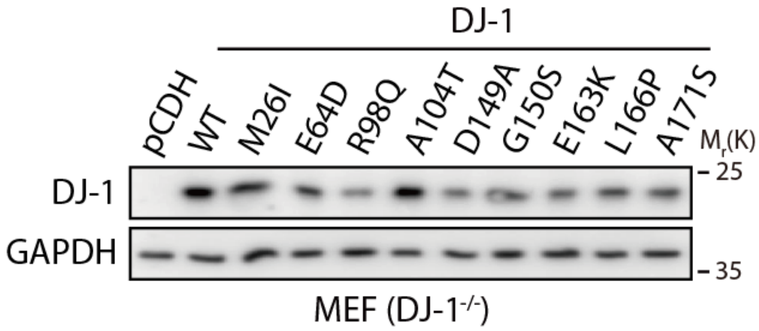

Raw data of western blot:

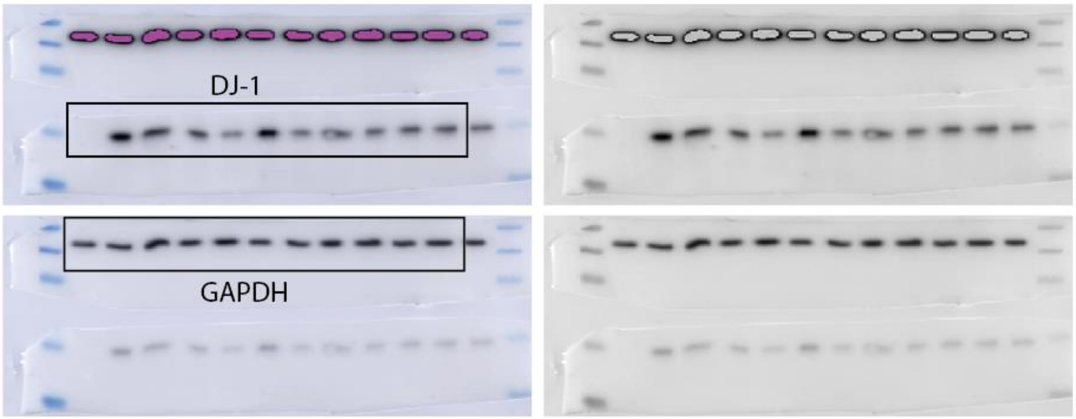

Figure 3a

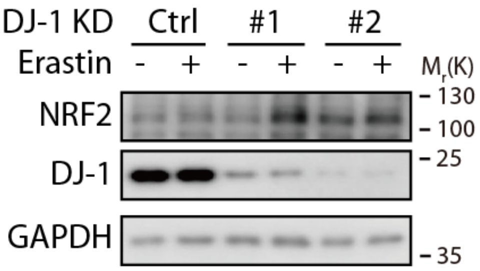

Raw data of western blot:

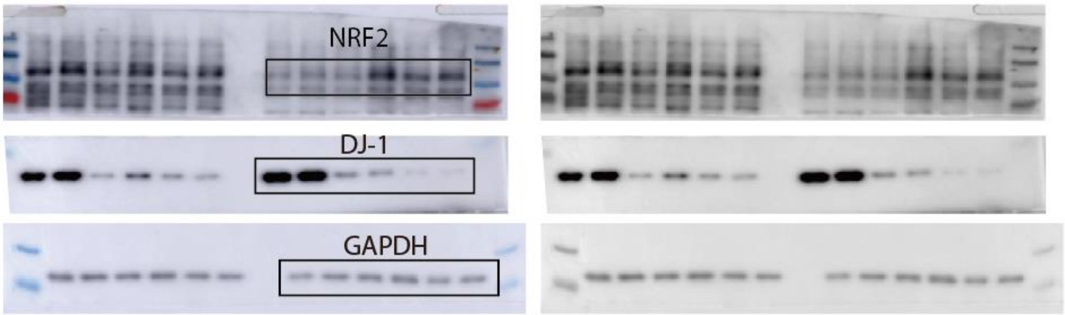

Figure 5g

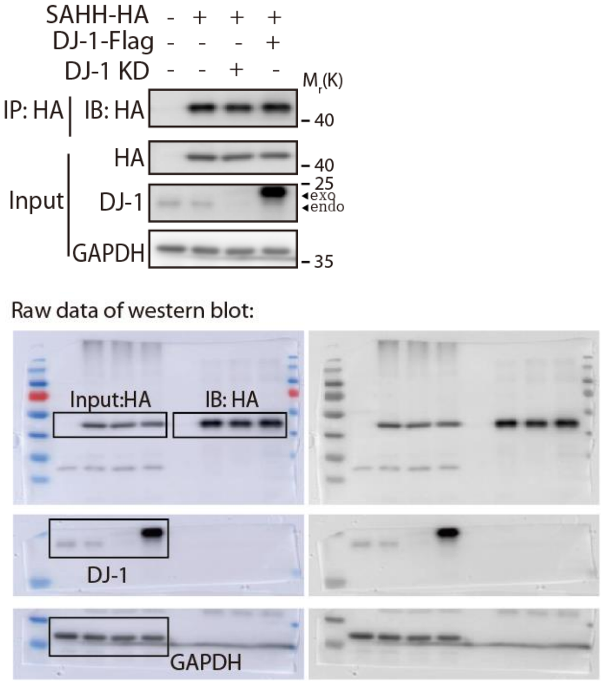

Figure 5i

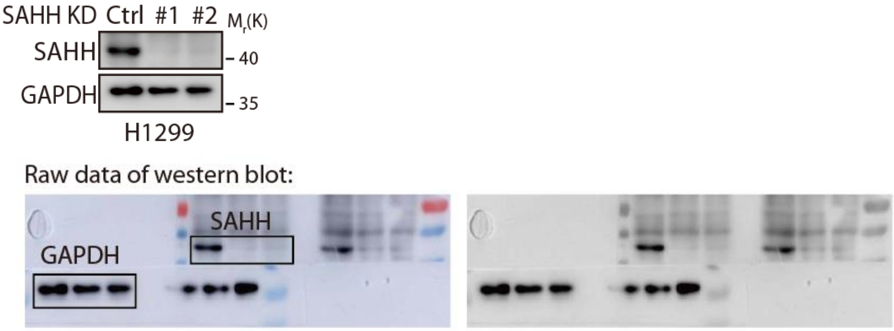

Figure 5k

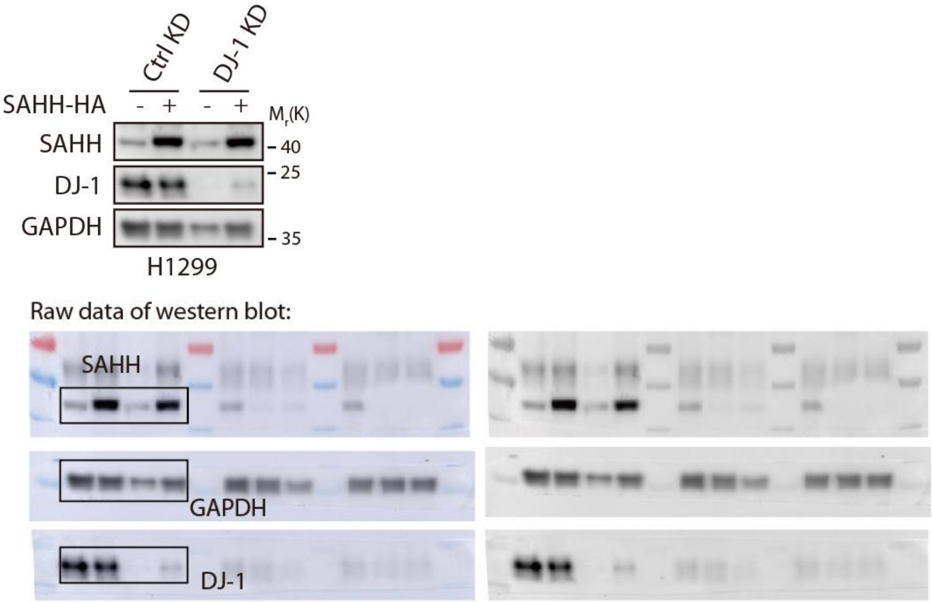

Figure 6c (left view)

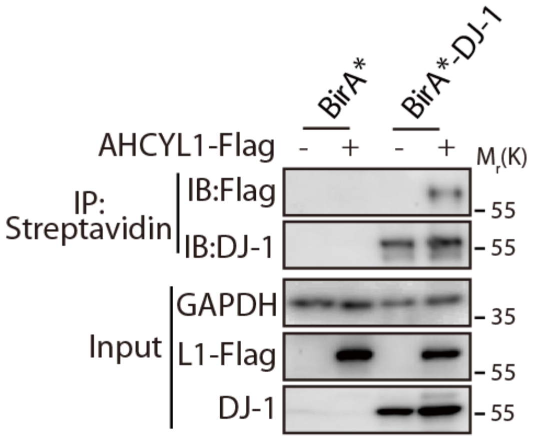

Raw data of western blot:

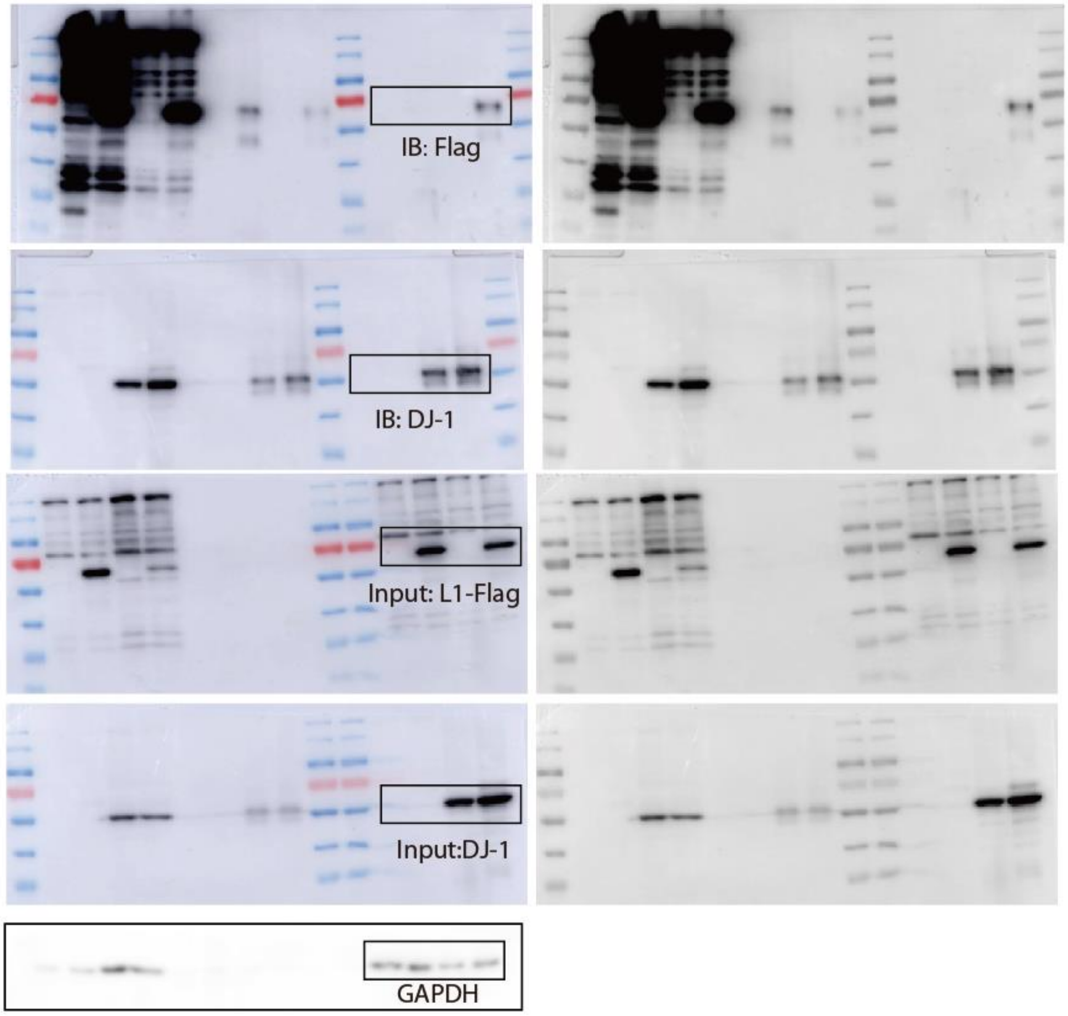

Figure 6c (right view)

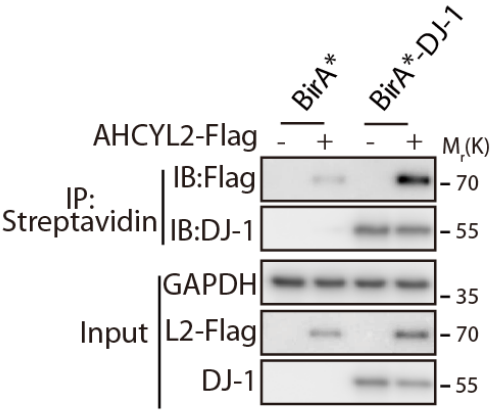

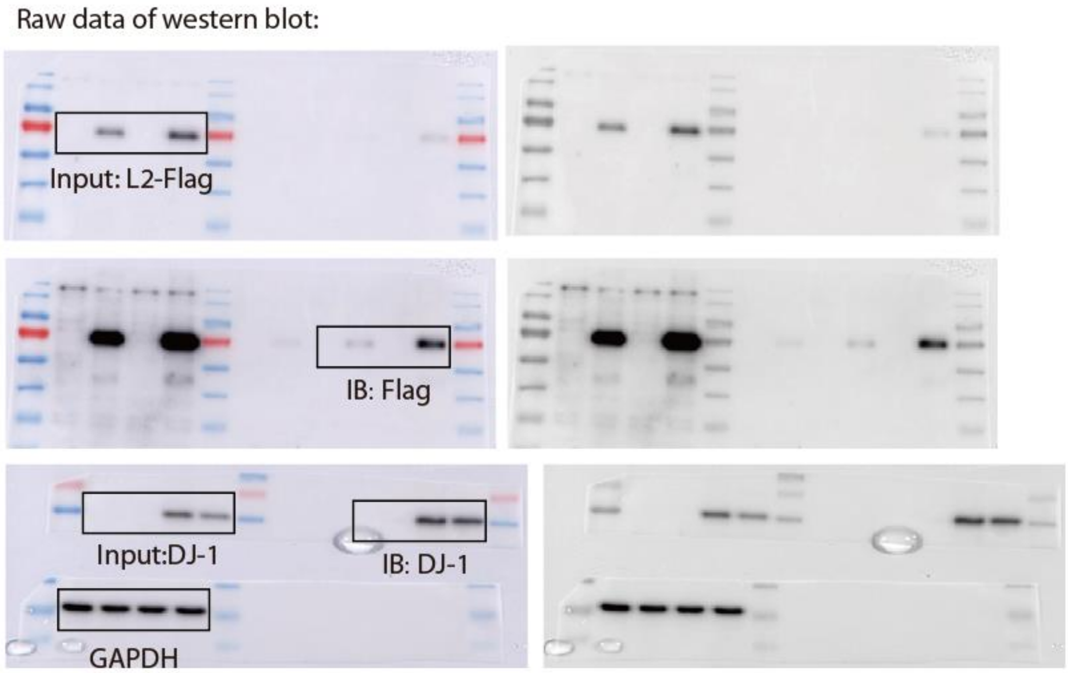

Figure 6d

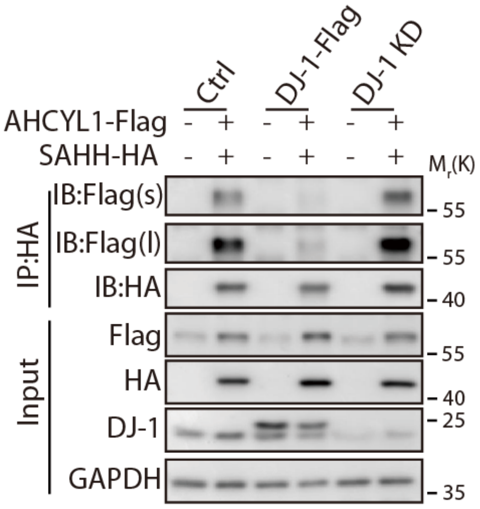

Raw data of western blot:

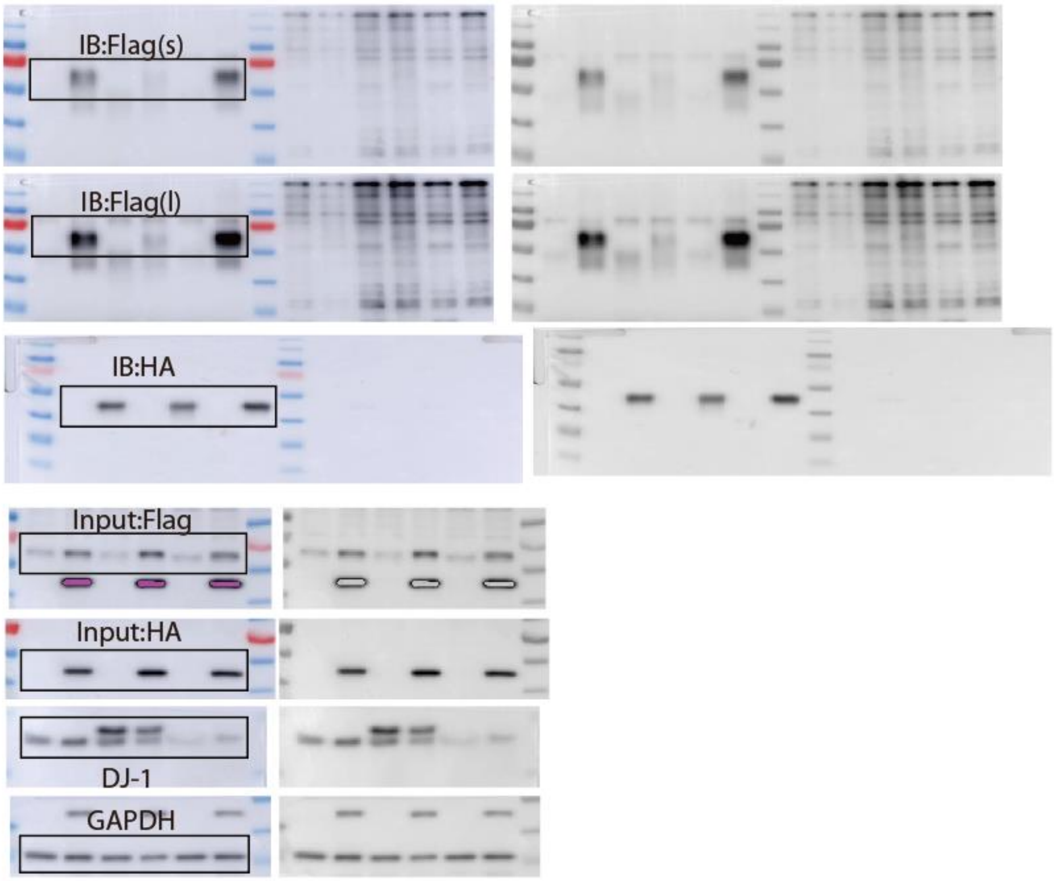

Figure 6e

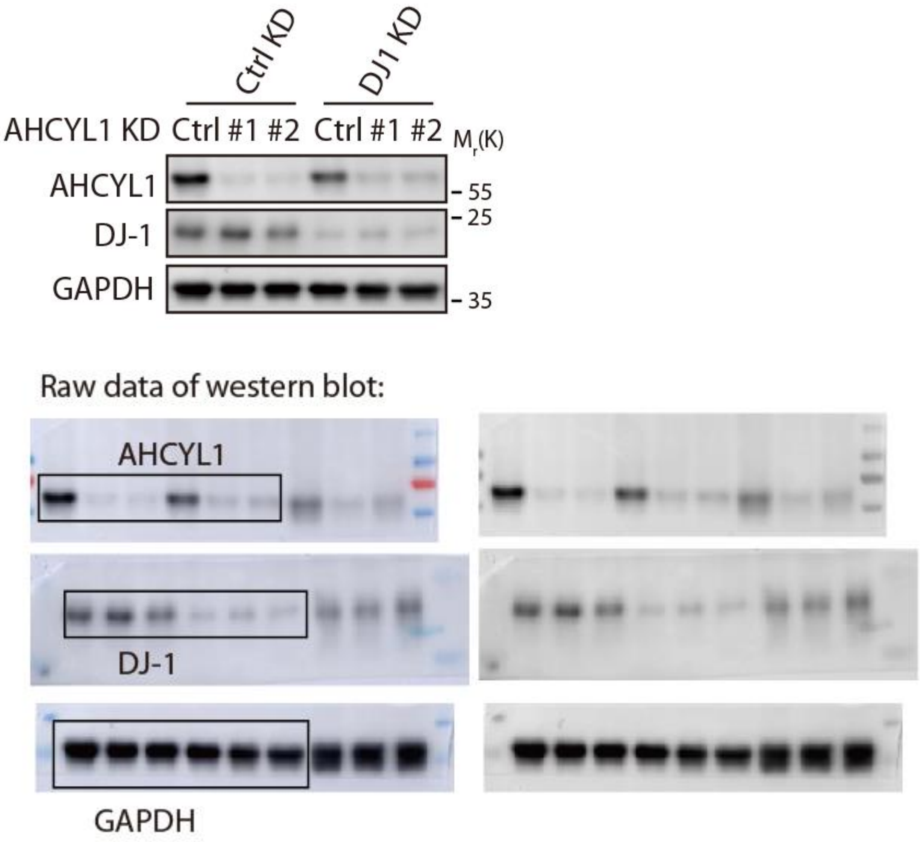

Figure 6g

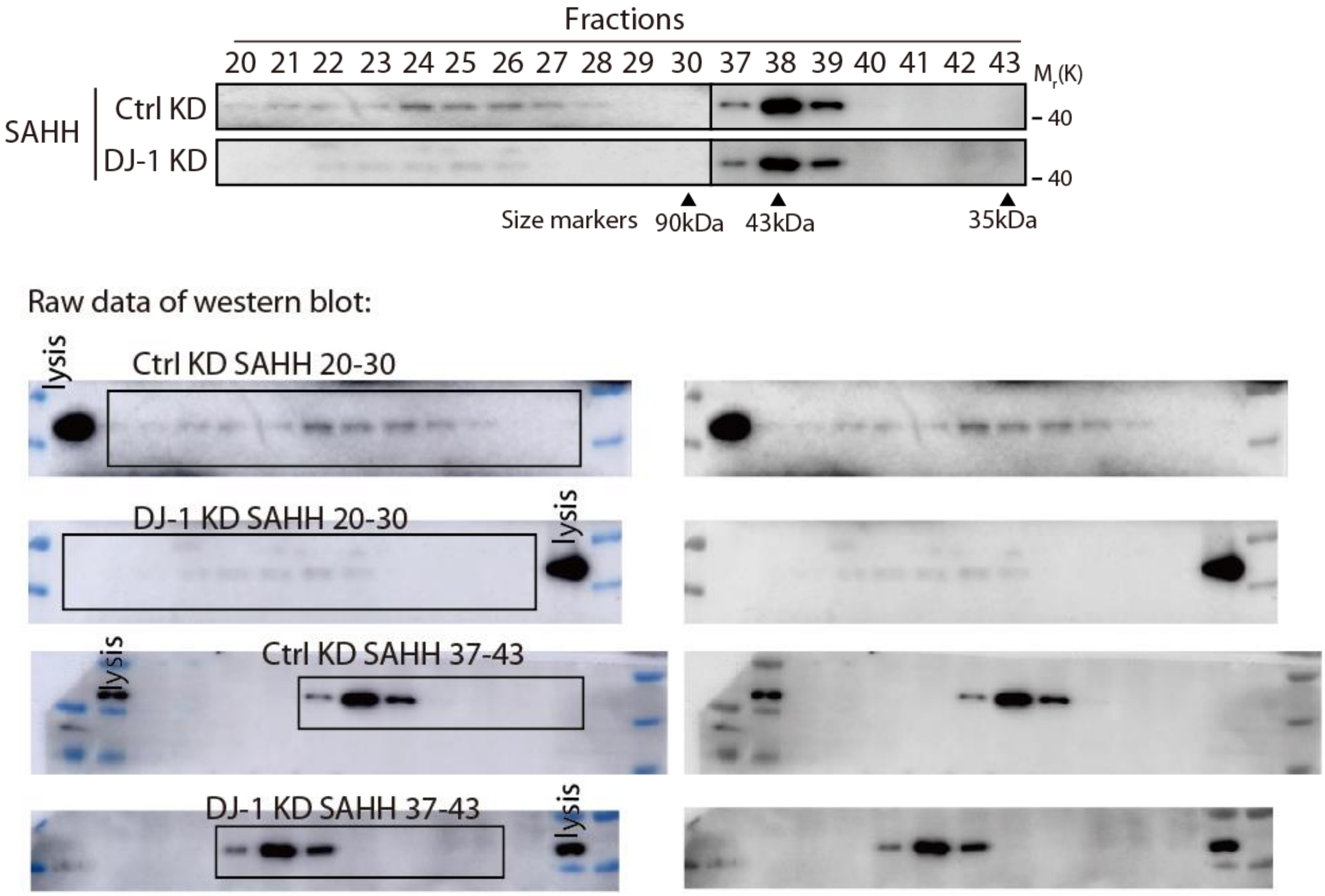

Supplementary Figure 1a

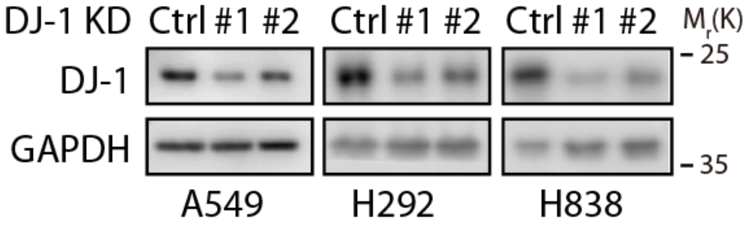

Raw data of western blot:

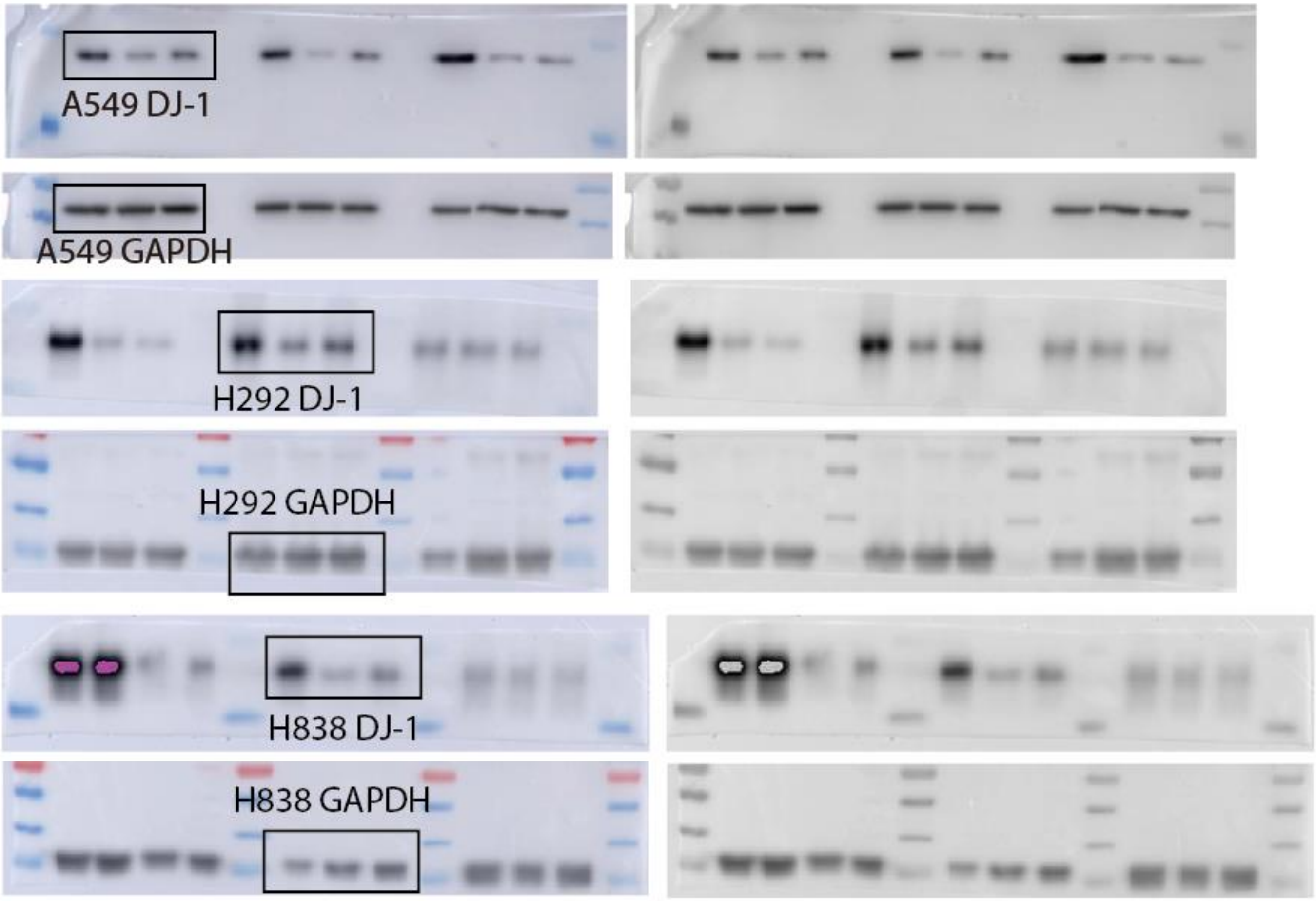

Supplementary Figure 1d

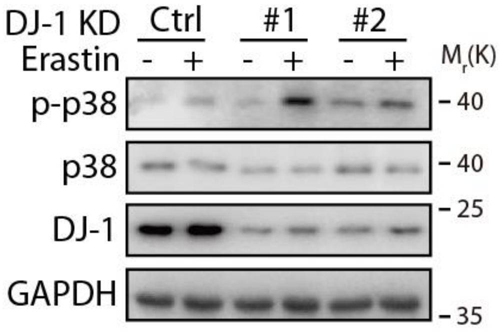

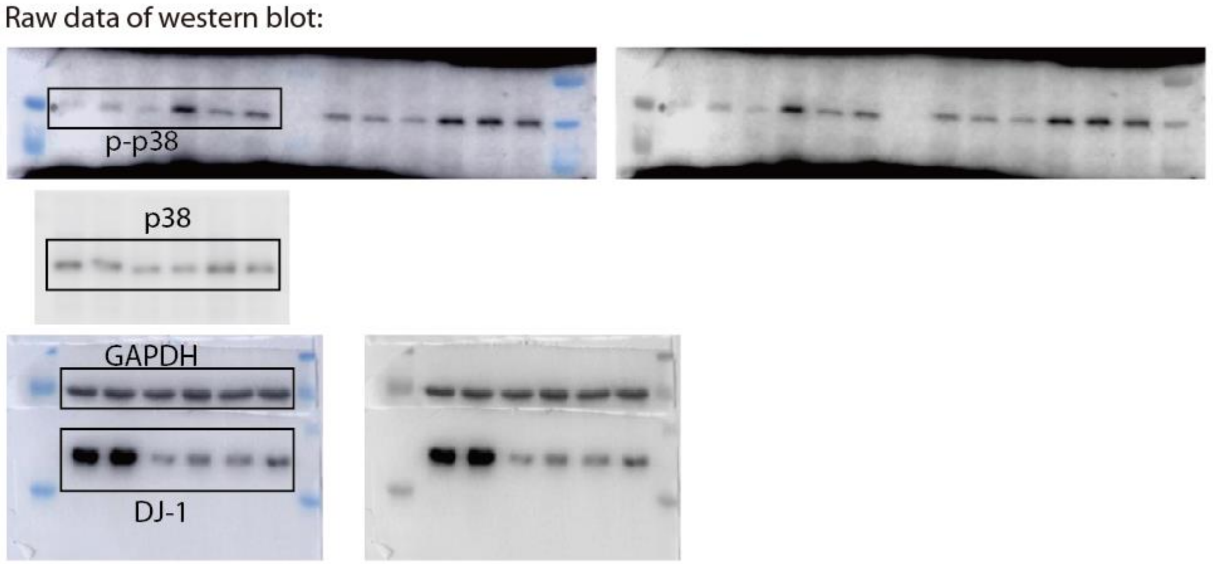

Supplementary Figure 1g

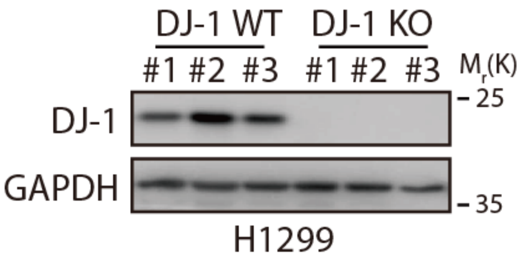

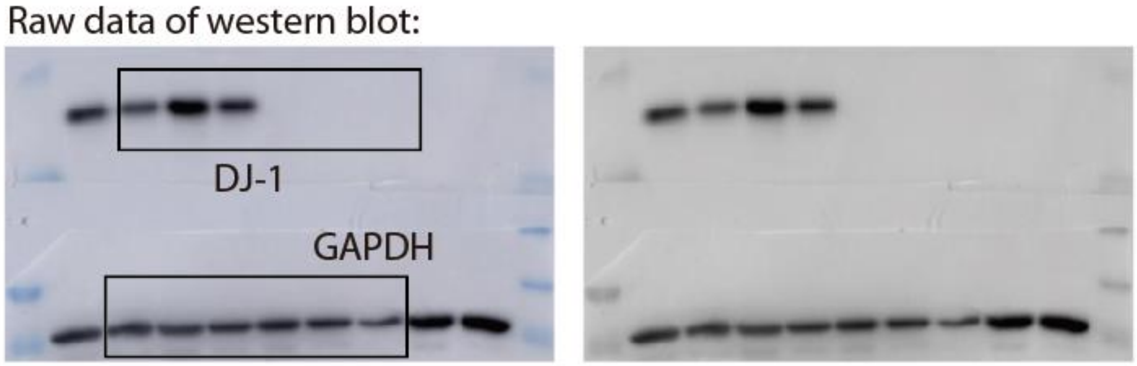

Supplementary Figure 2a

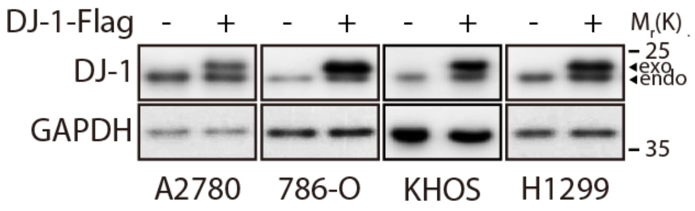

Raw data of western blot:

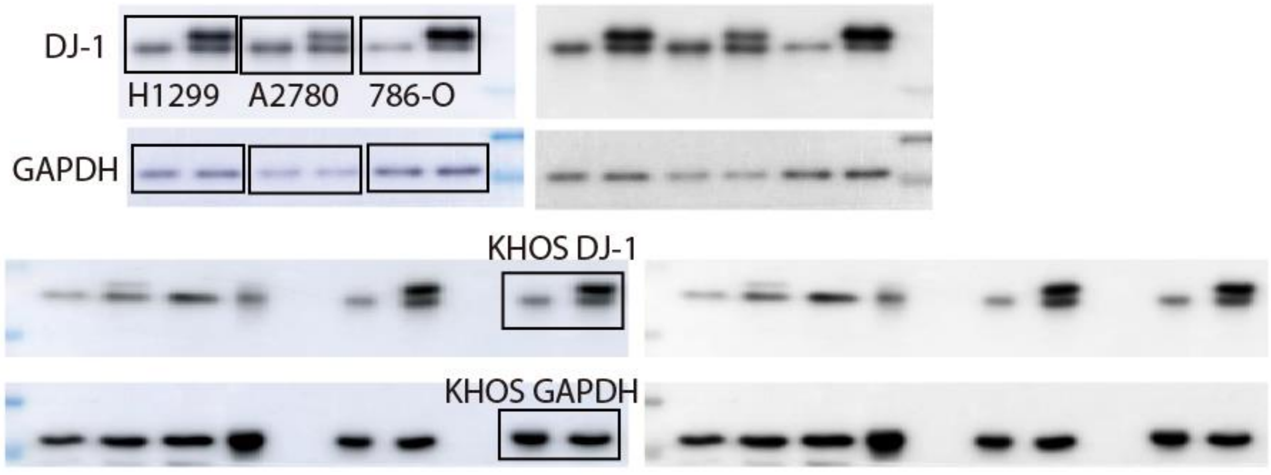

Supplementary Figure 3a

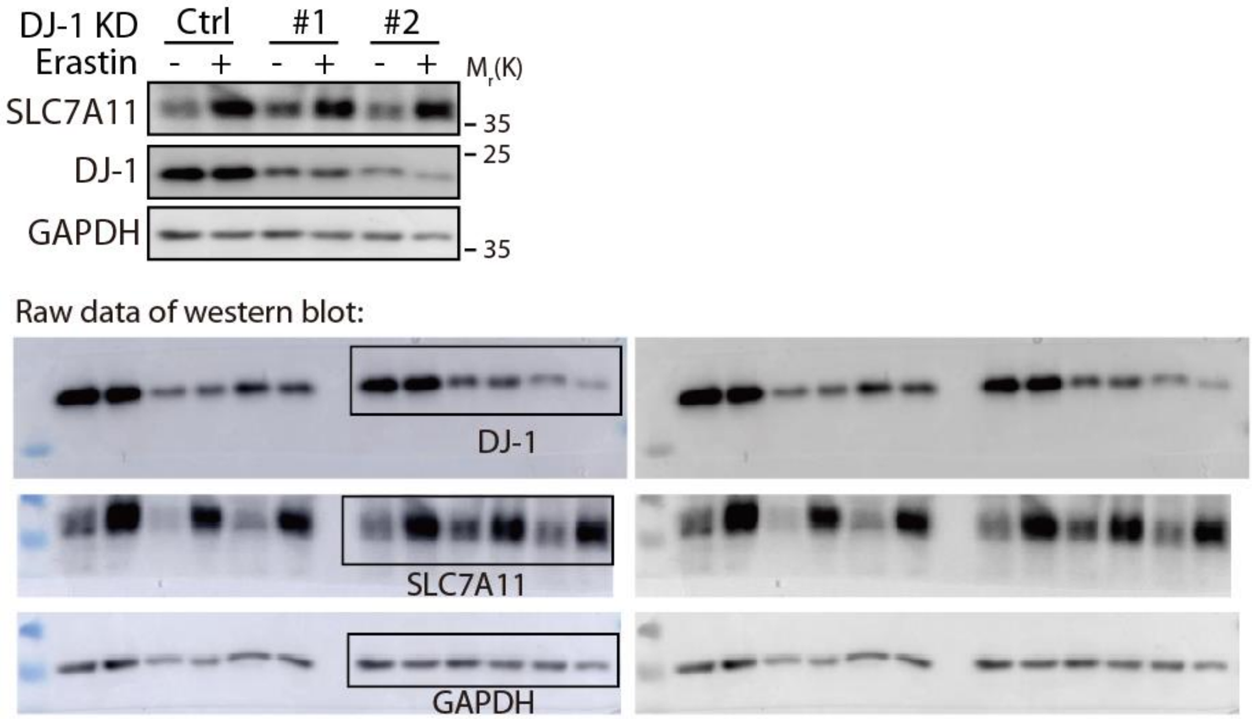

Supplementary Figure 3f

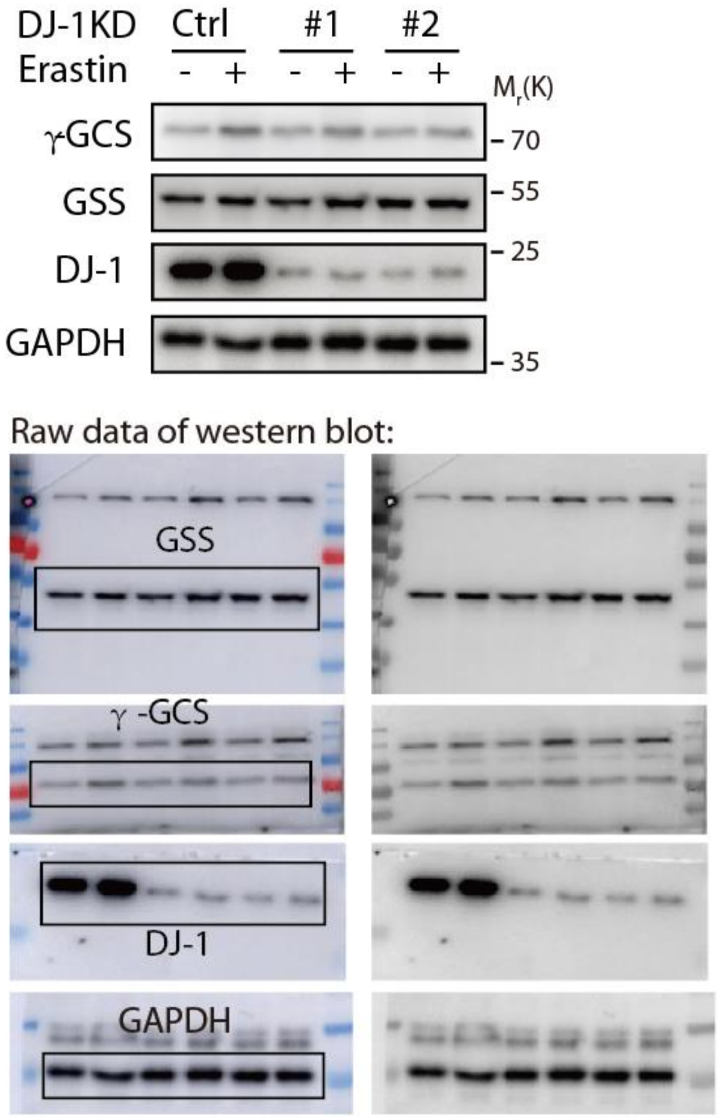

Supplementary Figure 4h

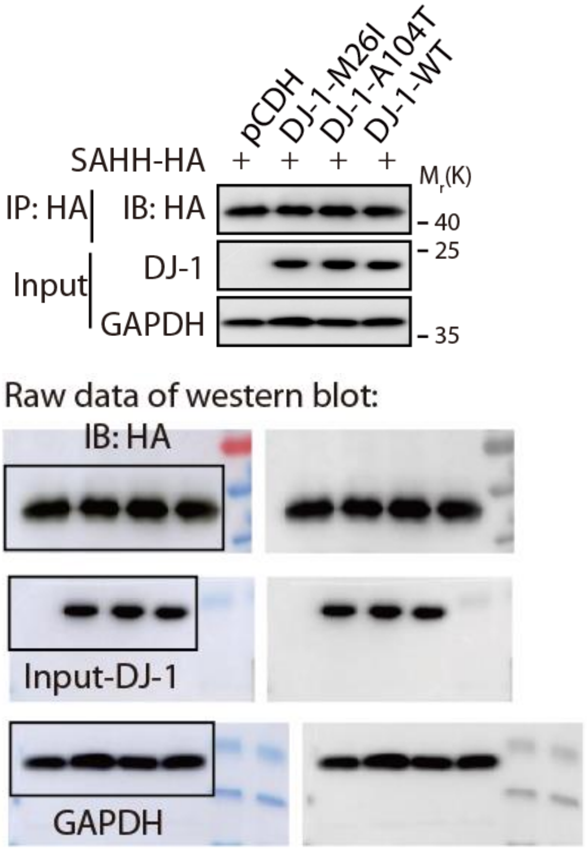

Supplementary Figure 5a

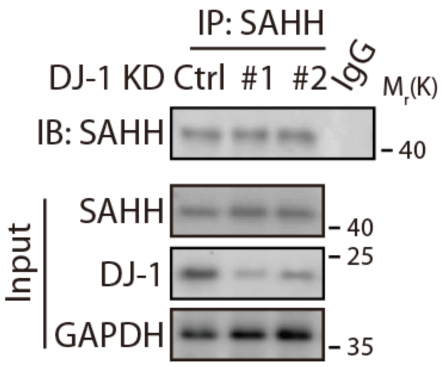

Raw data of western blot:

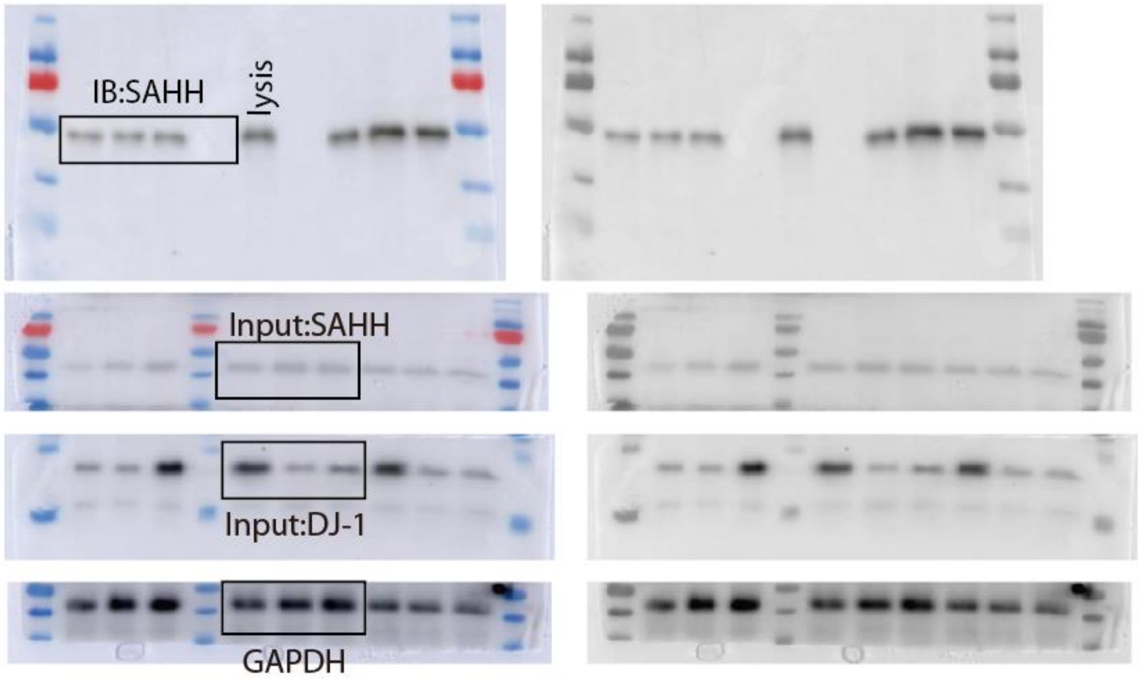

Supplementary Figure 5f

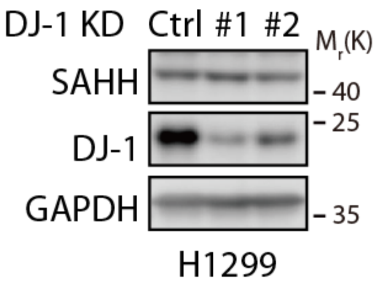

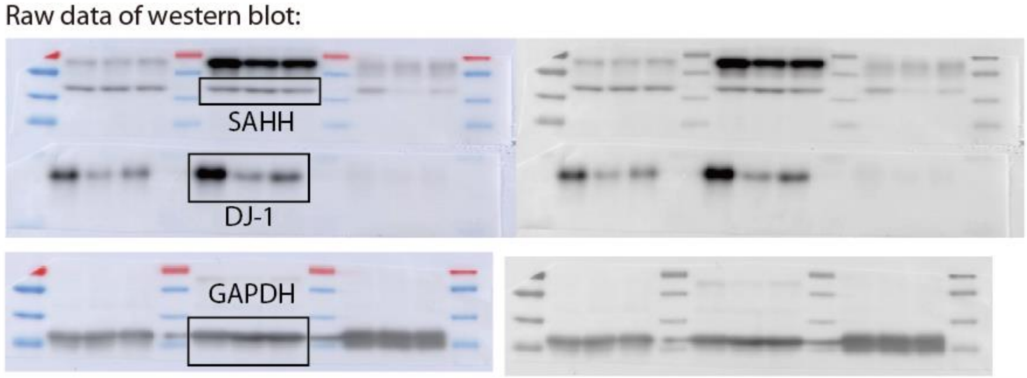

Supplementary Figure 5g

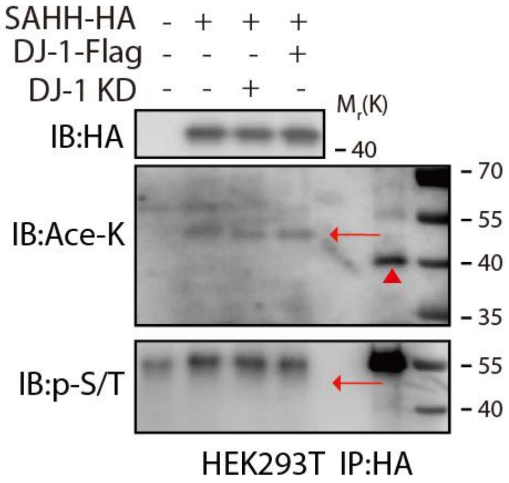

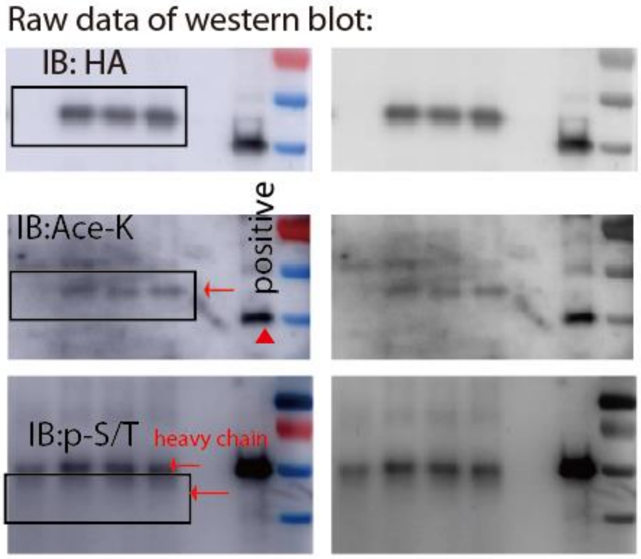

Supplementary Figure 5h

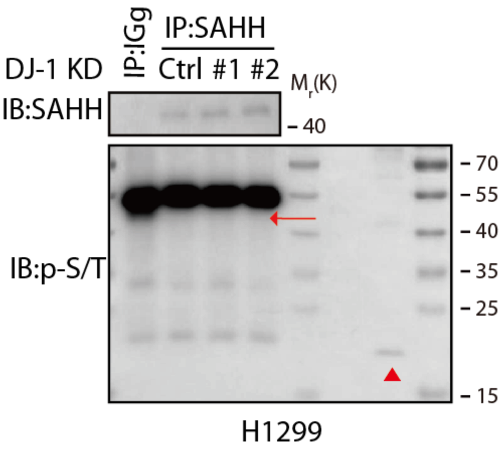

Raw data of western blot:

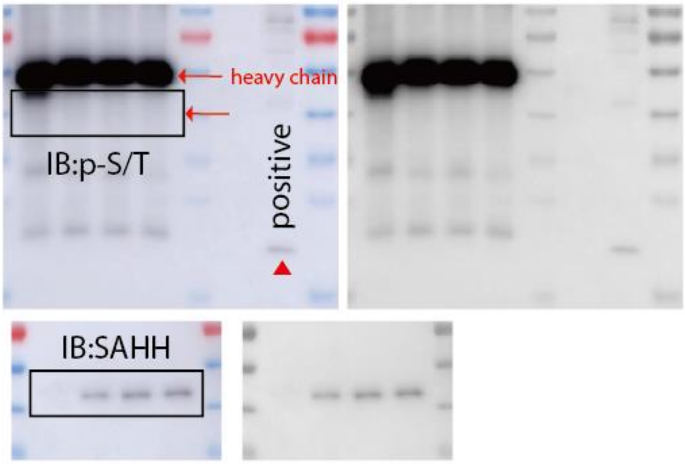

Supplementary Figure 5i

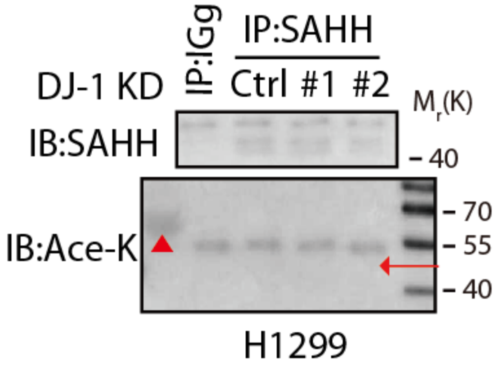

Raw data of western blot:

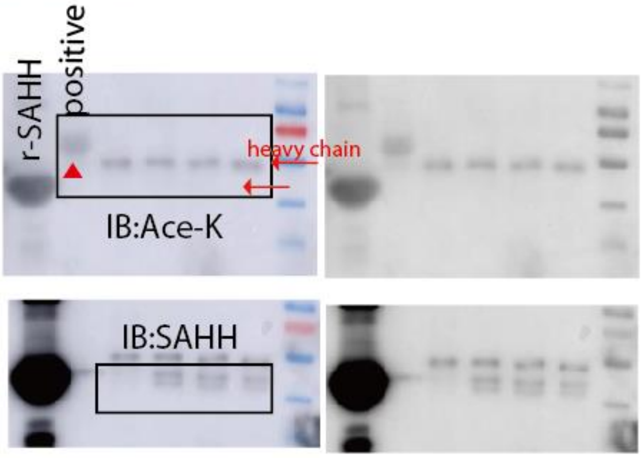

Supplementary Figure 6b

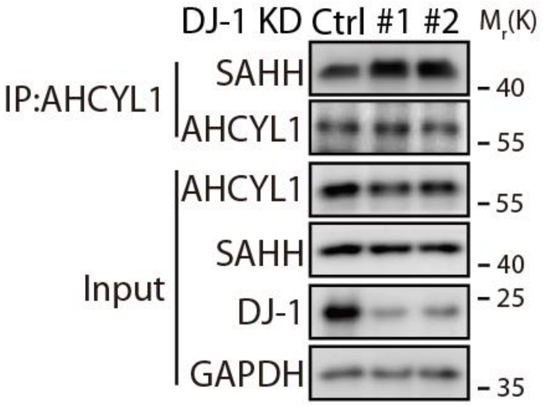

Raw data of western blot:

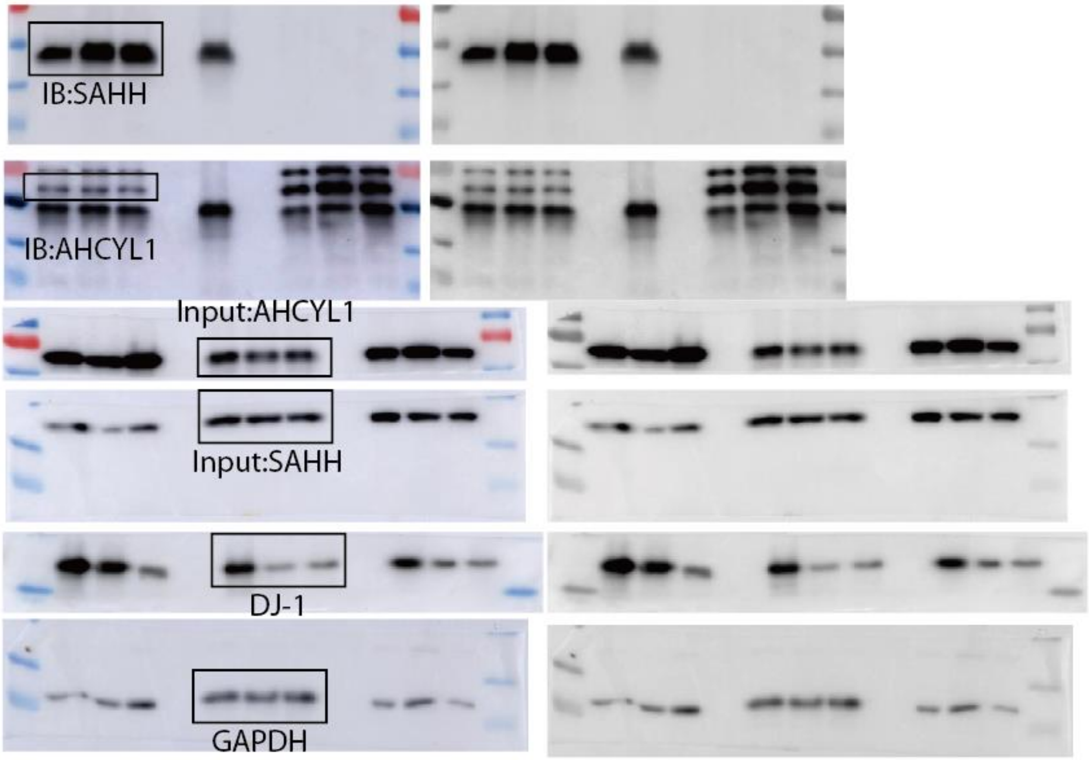

Supplementary Figure 6c

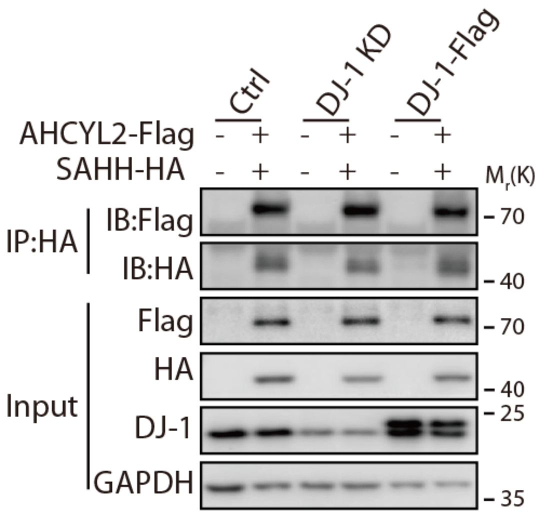

Raw data of western blot:

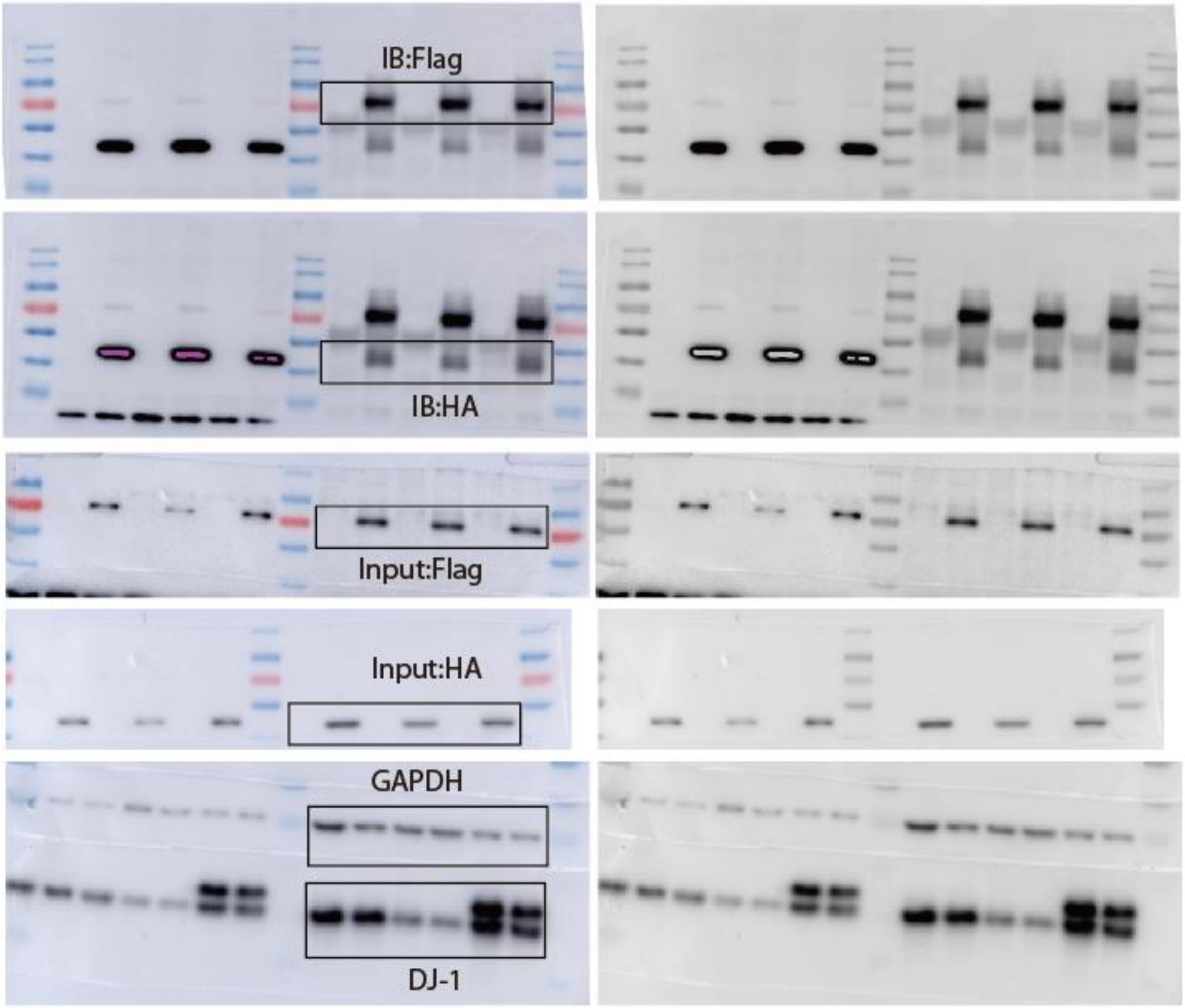

Supplementary Figure 6h

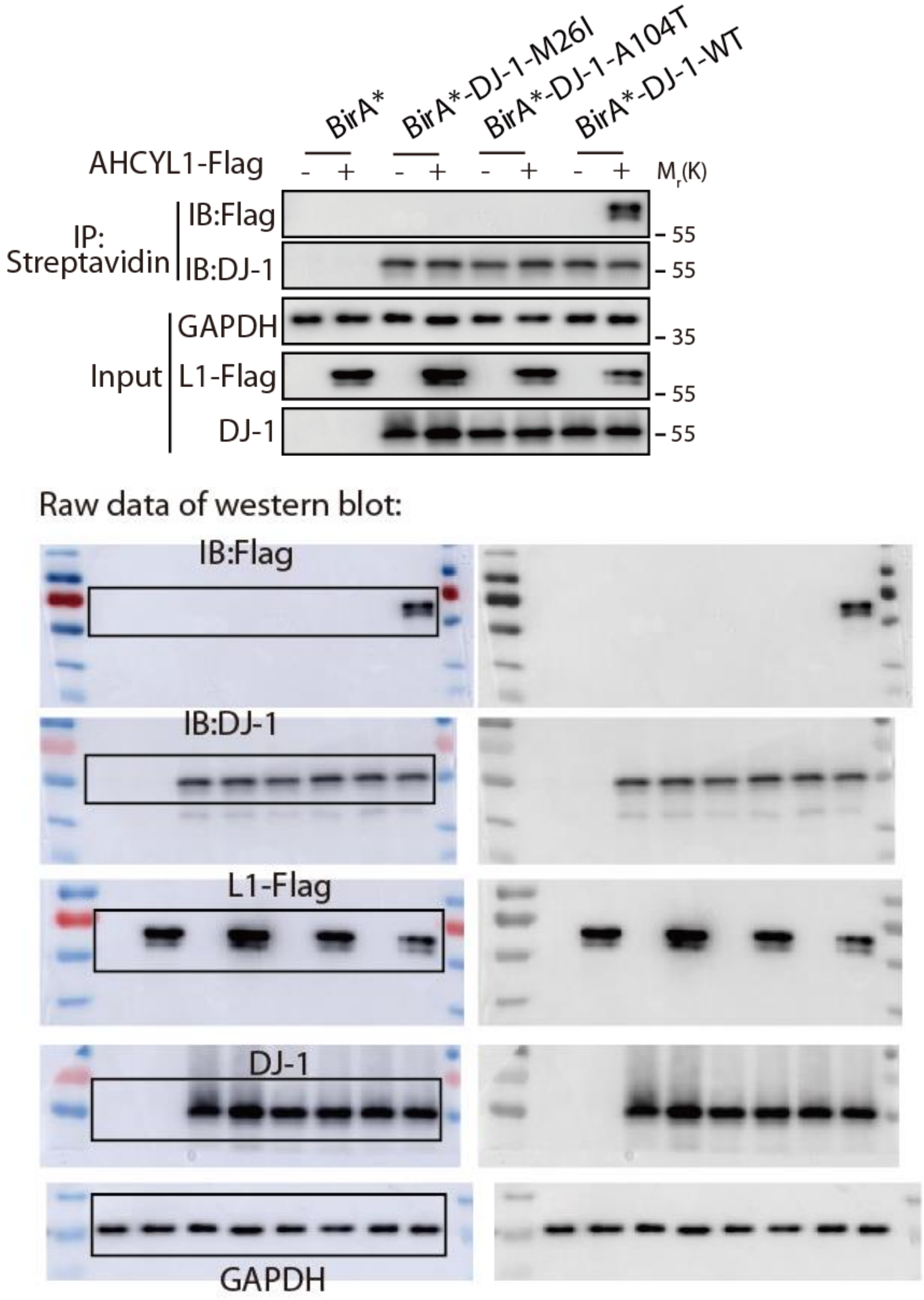

Supplementary Figure 6i

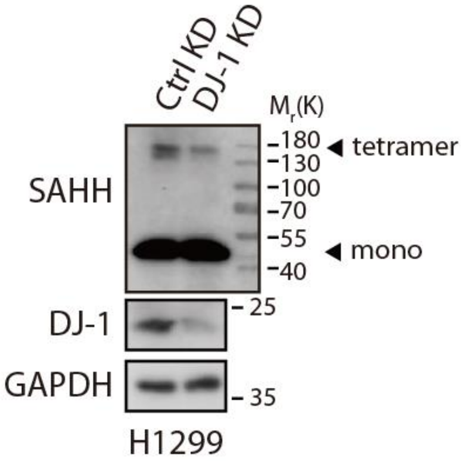

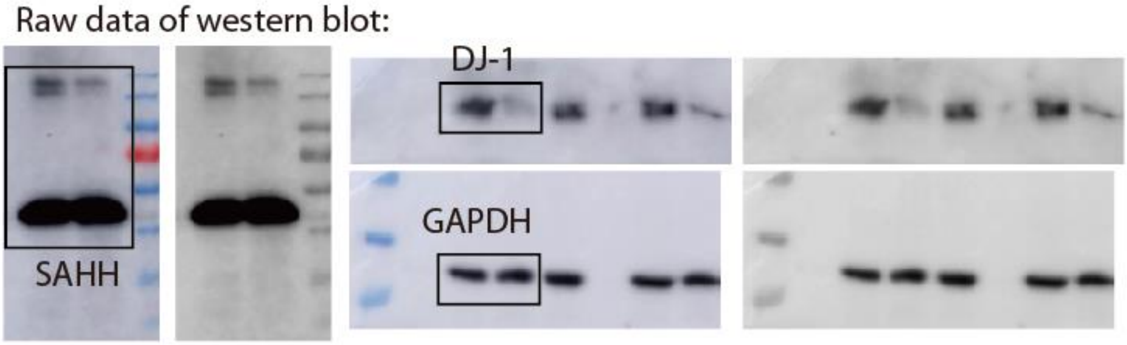

Supplementary Figure 6j

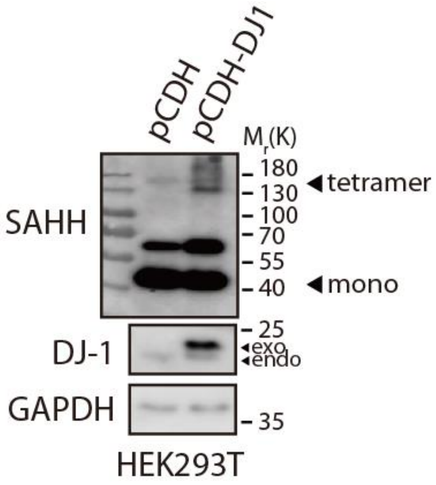

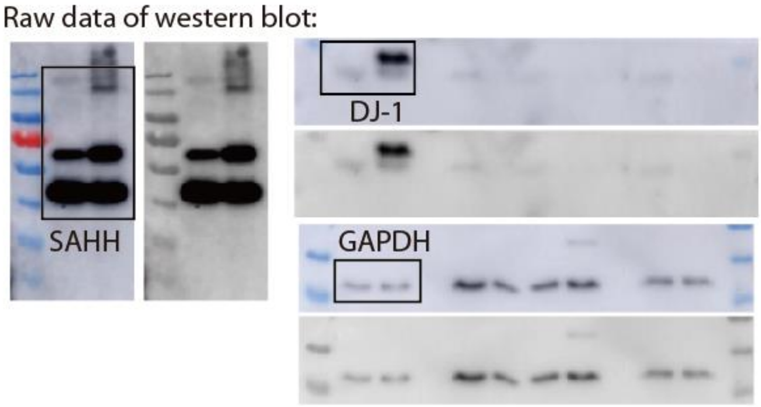

FACS Sequential Gating Strategies

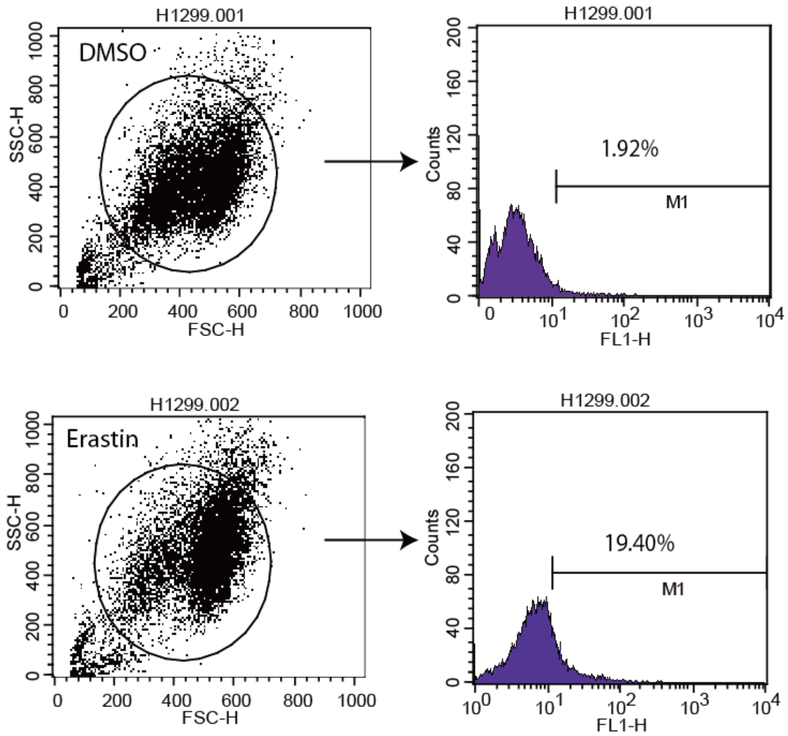

Gating strategy to determine the percentage of cells with Lipid ROS: 10,000 cells are analyzed per condition, the percentage in vehicle group is controlled within 5%, and then the percentage of the other groups are analyzed.

Raw data in graphs and charts

Figure 1b

H1299						
Lipid ROS	DMSO			Erastin		
	Ctrl KD	DJ-1 KD#1	DJ-1 KD#2	Ctrl KD	DJ-1 KD#1	DJ-1 KD#2
1st	1.92	3.07	2.39	19.4	59.06	34.62
2nd	1.41	2.89	1.45	27.29	63.28	35.06
3rd	1.16	3.36	5.93	28.3	61.28	43.46
average	1.50	3.11	3.257	25.00	61.21	37.71
SD	0.39	0.24	2.36	4.87	2.11	4.98

Figure 1d

Cell Viability%	Ctrl KD			DJ-1 KD#1			DJ-1 KD #2		
Erastin(μM)	1st	2nd	3rd	1st	2nd	3rd	1st	2nd	3rd
0	100	100	100	100	100	100	100	100	100
1	83.82	74.74	91.61	36.92	43.20	46.97	63.64	71.97	66.15
2	73.12	65.59	86.07	18.52	20.20	26.66	40.32	36.10	21.46
4	38.05	44.11	44.84	13.88	10.52	20.46	16.81	28.69	19.86

Figure 1e

Lipid ROS	DMSO			Erastin		
	Ctrl KD	DJ-1 KD#1	DJ-1 KD#2	Ctrl KD	DJ-1 KD#1	DJ-1 KD#2
1st	1.53	9.45	1.86	25.14	63.53	43.2
2nd	1.87	7.18	1.12	20.08	59.42	40.13
3rd	1.1	12.44	1.21	29.82	60.48	41.42
Lipid ROS	Fer-1			Erastin+Fer-1		
	Ctrl KD	DJ-1 KD#1	DJ-1 KD#2	Ctrl KD	DJ-1 KD#1	DJ-1 KD#2
1st	0.94	0.66	0.69	3.64	3.49	3.97
2nd	1.59	4.86	1.96	3.85	3.97	3.84
3rd	0.39	1.24	0.84	2.22	5.33	3.04

Figure 1f

Cell Viability%		DMSO			Fer-1		
		Ctrl KD	DJ-1 KD#1	DJ-1 KD#2	Ctrl KD	DJ-1 KD#1	DJ-1 KD#2
	1st	100	100	100	94.23	87.45	102.23
	2nd	100	100	100	98.51	105.24	109.34
	3rd	100	100	100	86.89	82.21	84.71
		Erastin			Erastin+Fer-1		
		Ctrl KD	DJ-1 KD#1	DJ-1 KD#2	Ctrl KD	DJ-1 KD#1	DJ-1 KD#2
	1st	63.15	19.09	20.68	95.25	93.02	96.80
	2nd	65.63	10.03	30.30	113.47	108.04	116.57
	3rd	76.06	20.60	31.44	89.86	77.98	92.78

Figure 1g

Lipid ROS	DMSO					
	DJ-1 WT#1	DJ-1 WT#2	DJ-1 WT#3	DJ-1 KO #1	DJ-1 KO #2	DJ-1 KO #3
1st	1.47	3.57	5.75	13.5	11.02	21.2
2nd	2.48	3.15	6.49	13.05	25.86	13.83
3rd	5.09	3.92	7.35	9.08	16.47	15.91
Lipid ROS	Erastin					
	DJ-1 WT#1	DJ-1 WT#2	DJ-1 WT#3	DJ-1 KO #1	DJ-1 KO #2	DJ-1 KO #3
1st	15.75	13.66	24.56	67.1	60.18	94.8
2nd	17.65	19.95	21.34	57.49	77.45	61.55
3rd	20.14	21.13	24.78	68.18	61.12	77.58

Figure 1h

Cell Viability%	DJ-1 WT#1			DJ-1 WT#2			DJ-1 WT#2		
Erastin(μM)	1st	2nd	3rd	1st	2nd	3rd	1st	2nd	3rd
0.0	100.0	100.0	100.0	100.0	100.0	100.0	100.0	100.0	100.0
1.0	96.7	93.9	91.4	87.4	93.4	102.0	92.0	90.4	92.1
2.0	73.2	88.6	86.6	65.7	71.4	82.2	84.8	75.1	76.6
4.0	41.9	51.9	66.1	47.1	47.6	55.9	59.7	55.2	58.9
Cell Viability%	DJ-1 KO#1			DJ-1 KO#2			DJ-1 KO#3		
Erastin(μM)	1st	2nd	3rd	1st	2nd	3rd	1st	2nd	3rd
0.0	100.0	100.0	100.0	100.0	100.0	100.0	100.0	100.0	100.0
1.0	66.4	69.0	43.3	59.1	60.4	60.6	54.9	67.0	53.2
2.0	36.1	37.6	24.3	35.8	37.2	37.7	12.8	16.0	20.1
4.0	14.6	16.2	10.8	15.6	17.8	19.1	2.3	2.1	3.2

Figure 2b

Lipid ROS	DMSO		Erastin	
	pCDH	DJ-1	pCDH	DJ-1
1st	3.56	3.4	39.12	19.98
2nd	2.6	3.93	39.24	12.87
3rd	3.1	3.52	32.98	19.17

Figure 2d

Viability%	DMSO		Erastin	
	pCDH	DJ-1	pCDH	DJ-1
1st	100	100	65.34	82.23
2nd	100	100	69.28	87.97
3rd	100	100	56.96	84.55

Figure 2f

	Viability%	pCDH	WT	M26I	E64D	R98Q	A104T	D149A	G150S	E163K	L166P	A171S
DMSO	1st	100	100	100.00	100.00	100	100	100.00	100.00	100	100	100.00
	2nd	100	100	100.00	100.00	100	100	100.00	100.00	100	100	100.00
	3rd	100	100	100.00	100.00	100	100	100.00	100.00	100	100	100.00
Erastin	Viability%	pCDH	WT	M26I	E64D	R98Q	A104T	D149A	G150S	E163K	L166P	A171S
	1st	64.25	87.98	58.75	66.32	68.48	71.88	65.34	65.93	67.56	63.84	67.79
	2nd	64.80	80.02	62.91	58.28	52.73	43.13	55.01	59.69	52.22	44.48	50.20
	3rd	66.01	85.55	72.84	70.69	69.24	67.16	69.96	70.35	75.61	71.01	72.11

Figure 3b

	RT-PCR	DMSO			Erastin		
PARK7		Ctrl KD	DJ-1 KD#1	DJ-1 KD#2	Ctrl KD	DJ-1 KD#1	DJ-1 KD#2
	1st	1	0.1431153	0.319995	1.236419	0.1676755	0.3012477
	2nd	1	0.0947591	0.3102289	0.8520126	0.1307277	0.2605516
	3rd	1	0.1432213	0.3445806	1.022459	0.1863051	0.3631594
	average	1.0	0.1	0.3	1.0	0.2	0.3

	RT-PCR	DMSO			Erastin		
HMOX1		Ctrl KD	DJ-1 KD#1	DJ-1 KD#2	Ctrl KD	DJ-1 KD#1	DJ-1 KD#2
	1st	1	0.8978873	0.9575469	9.103429	20.46168	14.46671
	2nd	1	0.7723718	0.9903331	8.120993	18.67486	12.40671
	3rd	1	0.9875257	0.807406	5.80926	14.49006	10.26945
	average	1.0	0.9	0.9	7.7	17.9	12.4

	RT-PCR	DMSO			Erastin		
NQO1		Ctrl KD	DJ-1 KD#1	DJ-1 KD#2	Ctrl KD	DJ-1 KD#1	DJ-1 KD#2
	1st	1	1.2028165	1.6329055	1.3859409	2.22152487	1.65260827
	2nd	1	1.2274655	1.5505746	1.3845477	2.32052128	1.736374
	3rd	1	0.9511956	1.052173	1.0412481	2.16219389	2.15845525
	average	1.0	1.1	1.4	1.3	2.2	1.8

	RT-PCR	DMSO			Erastin		
FTH1		Ctrl KD	DJ-1 KD#1	DJ-1 KD#2	Ctrl KD	DJ-1 KD#1	DJ-1 KD#2
	1st	1	1.683054	1.697155	1.779374	2.632023	2.005453
	2nd	1	1.237224	1.593639	1.355221	2.261182	1.72584
	3rd	1	0.9005179	1.174268	1.15626	2.198921	1.109866
	average	1.0	1.3	1.5	1.4	2.4	1.6

	RT-PCR	DMSO			Erastin		
FTL		Ctrl KD	DJ-1 KD#1	DJ-1 KD#2	Ctrl KD	DJ-1 KD#1	DJ-1 KD#2
	1st	1	1.457133	1.534522	1.452414	2.033495	1.660595
	2nd	1	1.152367	1.67094	1.373639	2.301974	1.908602
	3rd	1	1.054884	0.9399562	1.13939	1.641738	1.451437
	average	1.0	1.2	1.4	1.3	2.0	1.7

	RT-PCR	DMSO			Erastin		
GCLM		Ctrl KD	DJ-1 KD#1	DJ-1 KD#2	Ctrl KD	DJ-1 KD#1	DJ-1 KD#2
	1st	1	1.077741	1.280183	1.912928	2.398178	1.623199
	2nd	1	1.098732	1.361479	1.70558	2.60857	2.186055
	3rd	1	1.174285	1.093652	1.804077	3.698576	2.423768
	average	1.0	1.1	1.2	1.8	2.9	2.1

Figure 3c

	RT-PCR	DMSO		Erastin	
		Ctrl KD	DJ-1 KD#1	Ctrl KD	DJ-1 KD#1
PARK7	1st	1	0.142843	0.935299	0.132448
	2nd	1	0.051154	1.193749	0.146148
	3rd	1	0.209642	0.90532	0.194252
	average	1	0.1345463	1.011456	0.157616
	SD	0	0.0795691	0.1585804	0.032459

	RT-PCR	DMSO		Erastin	
		Ctrl KD	DJ-1 KD#1	Ctrl KD	DJ-1 KD#1
NRF2	1st	1	1.102287	0.309605	0.221212
	2nd	1	0.911617	0.373453	0.314471
	3rd	1	1.02172	0.230206	0.256939
	average	1	1.0118747	0.3044213	0.264207
	SD	0	0.0957155	0.071764	0.047052

Figure 3d

Cell Viability%	DMSO				Era 1			
	Ctrl KD	DJ-1 KD	NRF2 KD	Double KD	Ctrl KD	DJ-1 KD	NRF2 KD	Double KD
1st	100	100	100	100	84.62	52.41	54.99	50.07
2nd	100	100	100	100	91.53	53.68	56.92	49.71
3rd	100	100	100	100	85.55	47.13	55.42	44.09
Cell Viability%	Era 2				Era4			
	Ctrl KD	DJ-1 KD	NRF2 KD	Double KD	Ctrl KD	DJ-1 KD	NRF2 KD	Double KD
1st	76.39	35.76	41.01	25.82	66.69	27.92	29.55	18.31
2nd	78.67	45.63	41.39	25.96	74.72	29.16	27.86	19.79
3rd	75.67	32.36	35.23	18.78	65.94	21.34	31.47	9.10

Figure 4a

GSH(Fold Change)	DMSO			Erastin		
	Ctrl KD	DJ-1 KD#1	DJ-1 KD#2	Ctrl KD	DJ-1 KD#1	DJ-1 KD#2
1st	1	0.94	0.84	0.72	0.50	0.52
2nd	1	0.94	1.11	0.68	0.26	0.53
3rd	1	1.03	0.98	0.67	0.26	0.35

Figure 4b

Lipid ROS	DMSO			Erastin			Erastin+GSH			Erastin+NAC		
	Ctrl KD	DJ-1 KD#1	DJ-1 KD#2	Ctrl KD	DJ-1 KD#1	DJ-1 KD#2	Ctrl KD	DJ-1 KD#1	DJ-1 KD#2	Ctrl KD	DJ-1 KD#1	DJ-1 KD#2
1st	2.24	3.4	2.99	20.95	59.12	29.21	1.56	5.61	3.43	3.15	6.91	4.68
2nd	2.52	3.04	3.91	19.34	47.13	29.57	5.71	3.63	5.26	1.87	3.39	3.55
3rd	2.42	3.96	2.11	16.19	46.07	28.62	2.47	3.28	3.96	2.62	2.6	1.78
average	2	3	3	19	51	29	3	4	4	3	4	3

Figure 4c

Cell Viability%	DMSO			Erastin			Erastin+GSH			Erastin+NAC		
	Ctrl KD	DJ-1 KD#1	DJ-1 KD#2	Ctrl KD	DJ-1 KD#1	DJ-1 KD#2	Ctrl KD	DJ-1 KD#1	DJ-1 KD#2	Ctrl KD	DJ-1 KD#1	DJ-1 KD#2
1st	100	100	100	73.68	22.47	55.74	110.34	96.48	88.74	108.90	94.07	87.66
2nd	100	100	100	69.21	26.80	36.55	102.92	86.65	78.29	99.77	84.79	106.05
3rd	100	100	100	66.77	18.38	40.22	115.29	94.60	86.80	105.79	91.35	85.86

Figure 4d

Lipid ROS	DMSO			Erastin			Erastin+Met		
	Ctrl KD	DJ-1 KD#1	DJ-1 KD#2	Ctrl KD	DJ-1 KD#1	DJ-1 KD#2	Ctrl KD	DJ-1 KD#1	DJ-1 KD#2
1st	1.99	3.29	3.35	22.55	41.2	39	8.48	51.86	32.08
2nd	2.9	3.76	2.33	19.65	52.96	36.15	14.76	36.35	25.04
3rd	3.26	1.95	3.15	18.87	53.02	36.55	24.07	49.16	50.17
Lipid ROS	Erastin+SAM			Erastin+SAH			Erastin+Hcy		
	Ctrl KD	DJ-1	DJ-1	Ctrl KD	DJ-1	DJ-1	Ctrl KD	DJ-1	DJ-1
1st	20.3	48.6	41.56	19.22	54.79	43.63	1.07	4.68	3.37
2nd	18.79	52.12	41.22	18.54	53.33	38.05	2.47	4.31	2.63
3rd	19.91	38.08	31.45	20.01	36.93	27.43	2.46	3.73	5.5

Figure 4e

Cell Viability%	DMSO			Erastin			Erastin+Met		
	Ctrl KD	DJ-1 KD#1	DJ-1 KD#2	Ctrl KD	DJ-1 KD#1	DJ-1 KD#2	Ctrl KD	DJ-1 KD#1	DJ-1 KD#2
1st	100	100	100	66.477499	25.37068	40.906145	60.616212	21.979535	32.177828
2nd	100	100	100	63.50162	28.855344	34.382737	79.282996	19.375041	40.017734
3rd	100	100	100	75.55806	17.87007	25.55416	63.91020	25.33789	29.17899
Cell Viability%	Erastin+SAM			Erastin+SAH			Erastin+Hcy		
	Ctrl KD	DJ-1 KD#1	DJ-1 KD#2	Ctrl KD	DJ-1 KD#1	DJ-1 KD#2	Ctrl KD	DJ-1 KD#1	DJ-1 KD#2
1st	64.047152	32.480806	37.005704	74.146462	23.752017	37.912197	98.077851	95.920073	94.810229
2nd	56.343832	26.338738	37.73203	66.803153	45.345504	37.133466	96.441515	88.749447	87.367876
3rd	63.30474	40.01914	42.17754	61.54832	39.55842	31.20657	87.416205	93.722895	93.832111

Figure 4f

	RT-PCR	Ctrl KD		DJ-1 KD	
		DMSO	Erastin	DMSO	Erastin
PARK7	1st	1	1.0614232	0.0318845	0.0177997
	2nd	1	0.8990667	0.0606226	0.0020424
	3rd	1	0.9094083	0.1571267	0.117644
	average	1	0.96	0.08	0.05
CBS	RT-PCR	Ctrl KD		DJ-1 KD	
		DMSO	Erastin	DMSO	Erastin
	1st	1	1.53262	0.880259	2.2330259
	2nd	1	1.4010418	1.1785394	1.674813
	3rd	1	2.8108374	4.2515827	1.9345527
	average	1.00	1.91	2.10	1.95
MAT2A	RT-PCR	Ctrl KD		DJ-1 KD	
		DMSO	Erastin	DMSO	Erastin
	1st	1	0.688725	0.9317404	0.2411495
	2nd	1	0.9993071	0.8919285	0.3535534
	3rd	1	0.7583838	0.980779	0.5318163
	average	1.00	0.82	0.93	0.38
MS	RT-PCR	Ctrl KD		DJ-1 KD	
		DMSO	Erastin	DMSO	Erastin
	1st	1	0.9233823	1.9738331	1.7519966
	2nd	1	0.7528843	0.6070974	0.5650694
	3rd	1	1.0973319	0.738669	0.3885036
	average	1.00	0.92	1.11	0.90

	RT-PCR	Ctrl KD		DJ-1 KD	
		DMSO	Erastin	DMSO	Erastin
BHMT	1st	1	0.4052826	0.1753124	0.8111272
	2nd	1	1.7684679	0.3484443	1.3703078
	3rd	1	1.7100044	0.5555544	4.8736411
	average	1.00	1.29	0.36	2.35
MAT1A	RT-PCR	Ctrl KD		DJ-1 KD	
		DMSO	Erastin	DMSO	Erastin
	1st	1	2.4897485	1.0288267	3.2898003
	2nd	1	1.350038	1.1176744	1.8391001
	3rd	1	1.271913	0.6470728	2.5561987
	average	1.00	1.70	0.93	2.56
SAHH	RT-PCR	Ctrl KD		DJ-1 KD	
		DMSO	Erastin	DMSO	Erastin
	1st	1	0.9869165	0.6853914	1.0331144
	2nd	1	1.1586943	1.0234921	1.3750652
	3rd	1	1.5669944	1.1250585	1.5220334
	average	1.00	1.24	0.94	1.31
CTH	RT-PCR	Ctrl KD		DJ-1 KD	
		DMSO	Erastin	DMSO	Erastin
	1st	1	4.1828898	1.4519583	3.8437114
	2nd	1	1.6557674	1.8615444	1.2158793
	3rd	1	1.9493593	1.4544765	2.4233453
	4th	1	1.6318404	0.7432915	0.856485
	average	1.00	2.35	1.38	2.08

Figure 5a

H1299				
Hcy(nmol/mg)	DMSO		Erastin	
	Ctrl KD	DJ-1 KD	Ctrl KD	DJ-1 KD
1st	7.60	4.61	6.38	3.62
2nd	7.01	2.97	6.41	0.90
3rd	7.48	3.76	6.96	2.64

Figure 5b

H1299						
Hcy(nmol/mg)	DMSO					
	DJ-1 WT#1	DJ-1 WT#2	DJ-1 WT#3	DJ-1 KO#1	DJ-1 KO#2	DJ-1 KO#3
1st	8.97	7.06	9.41	3.85	2.16	3.82
2nd	6.65	7.53	5.50	1.85	1.12	2.18
3rd	6.70	5.09	7.00	1.64	2.04	3.70
Hcy(nmol/mg)	Erastin					
	DJ-1 WT#1	DJ-1 WT#2	DJ-1 WT#3	DJ-1 KO#1	DJ-1 KO#2	DJ-1 KO#3
1st	6.36	7.74	8.36	1.58	1.23	2.28
2nd	9.47	6.16	6.35	3.38	2.74	1.20
3rd	7.80	7.60	7.50	1.37	2.27	4.34

Figure 5c

MEF DJ-1 -/-				
Hcy(nmol/mg)	DMSO		Erastin	
	pCDH	DJ-1	pCDH	DJ-1
1st	3.35	4.74	3.92	4.85
2nd	3.01	5.09	2.20	4.28
3rd	3.61	6.88	3.20	6.49

Figure 5e

H1299				
Hcy(nmol/mg)	met(-) SAH(-)		met(-) SAH(+)	
	Ctrl KD	DJ-1 KD	Ctrl KD	DJ-1 KD
1st	1.00	0.65	1.42	0.72
2nd	1.00	0.67	1.17	0.66
3rd	1.00	0.68	1.24	0.65

Figure 5f

MEF DJ-1 -/-				
Hcy(nmol/mg)	met(-) SAH(-)		met(-) SAH(+)	
	pCDH	DJ-1	pCDH	DJ-1
1st	1.00	1.22	1.09	1.88
2nd	1.00	1.32	1.35	2.33
3rd	1.00	1.03	1.21	1.63

Figure 5h

time	Ctrl	SAHH	DJ-1 KD SAHH	DJ-1+SAHH
0:00:00	111.014	95.991	-15.617	-20.632
0:00:45	56.14	207.221	80.397	119.44
0:01:30	46.38	125.943	3.571	99.9
0:02:15	95.338	202.956	15.135	155.973
0:03:00	68.105	166.287	11.591	112.129
0:03:45	74.512	124.485	12.582	159.197
0:04:30	37.491	192.795	10.07	195.202
0:05:15	55.08	275.469	21.584	102.118
0:06:00	49.293	234.321	6.645	121.54
0:06:45	134.157	206.256	36.712	242.199
0:07:30	86.738	237.353	1.682	180.155
0:08:15	106.795	316.247	45.719	224.011
0:09:00	108.171	281.622	71.781	265.786
0:09:45	122.436	285.024	67.366	242.385
0:10:30	95.302	238.009	66.851	293.919
0:11:15	89.109	321.374	84.709	398.157
0:12:00	129.563	318.709	98.637	422.607
0:12:45	120.622	325.288	137.182	541.537
0:13:30	133.031	371.541	169.141	542.514
0:14:15	117.748	525.918	160.36	640.03
0:15:00	130.496	388.427	200.876	690.781
0:15:45	105.14	420.453	149.989	757.004
0:16:30	158.462	480.586	192.184	787.24
0:17:15	133.943	671.742	231.752	815.625
0:18:00	124.06	543.264	234.693	880.906
0:18:45	140.812	602.245	284.622	978.49
0:19:30	117.052	560.837	299.763	1004.884
0:20:15	120.923	601.788	332.557	968.403
0:21:00	115.704	655.813	312.656	1106.108
0:21:45	138.348	654.824	376.169	1049.707
0:22:30	106.339	827.635	350.1	1242.879
0:23:15	115.396	779.858	348.992	1281.168
0:24:00	124.689	724.89	397.47	1352.034
0:24:45	138.443	793.4	421.105	1382.796
0:25:30	111.798	744.772	407.062	1355.84
0:26:15	91.276	978.368	445.319	1480.771
0:27:00	133.225	884.073	461.213	1510.953
0:27:45	140.222	808.219	505.381	1521.502
0:28:30	107.838	835.027	507.902	1630.47
0:29:15	153.444	856.762	535.105	1664.541
0:30:00	112.698	906.05	516.79	1746.886
0:30:45	106.832	990.552	546.307	1731.646
0:31:30	151.232	930.824	571.949	1758.161
0:32:15	124.45	944.921	605.315	1840.583

0:33:00	128.574	983.724	614.502	1912.22
0:33:45	114.056	1013.866	590.902	1863.394
0:34:30	133.177	1061.618	600.133	1986.759
0:35:15	134.951	1072.478	626.074	1967.883
0:36:00	104.392	1144.685	616.578	2032.061
0:36:45	113.362	1317.646	610.894	2097.859
0:37:30	138.628	1171.222	667.397	2105.262
0:38:15	124.226	1178.144	673.186	2089.498
0:39:00	113.357	1201.716	660.656	2122.679
0:39:45	125.232	1280.54	730.615	2148.787
0:40:30	125.686	1411.016	683.081	2218.532
0:41:15	131.846	1257.45	744.848	2321.124
0:42:00	116.071	1246.176	720.945	2260.998
0:42:45	119.803	1274.509	787.926	2334.343
0:43:30	119.975	1469.697	739.855	2387.518
0:44:15	104.345	1503.497	715.356	2401.833
0:45:00	89.169	1484.531	797.016	2403.232

Figure 5j

Lipid ROS	DMSO			Erastin		
	Ctrl KD	SAHH KD#1	SAHH KD#2	Ctrl KD	SAHH KD#1	SAHH KD#2
1st	3.53	2.26	1.94	21.76	39.99	36.57
2nd	3.2	7.9	1.47	28.87	37.58	50.54
3rd	1.69	2.54	2.74	25.65	49.62	48.42

Figure 5l

Lipid ROS	DMSO				Erastin			
	Ctrl KD	Ctrl KD+SAHH	DJ-1 KD	DJ-1 KD+SAHH	Ctrl KD	Ctrl KD+SAHH	DJ-1 KD	DJ-1 KD+SAHH
1st	2.07	7.04	4.15	5.56	21.37	24.79	67.45	51.58
2nd	3.27	2.9	8.76	7.17	22.19	19.39	58.37	45.08
3rd	3.17	2.4	2.64	4.71	15.43	18.14	58.16	45.2

Figure 5m

Cell Viability%	DMSO				Erastin			
	Ctrl KD	Ctrl KD+SAHH	DJ-1 KD	DJ-1 KD+SAHH	Ctrl KD	Ctrl KD+SAHH	DJ-1 KD	DJ-1 KD+SAHH
1st	100	100	100	100	81.75	85.95	33.57	49.04
2nd	100	100	100	100	74.58	68.34	25.93	45.33
3rd	100	100	100	100	70.88	68.36	20.92	40.34

Figure 6f

Lipid ROS	DMSO					
	Ctrl KD	AHCYL1 KD#1	AHCYL1 KD#2	DJ-1 KD	DJ-1 KD+AHCYL1	DJ-1 KD+AHCYL1
1st	4.7	4.17	1.55	5.45	8.16	9.21
2nd	3.13	4.71	2.53	5.63	10.66	7.42
3rd	4.27	6.01	3.74	7.28	11.78	4.3
Lipid ROS	Erastin					
	Ctrl KD	AHCYL1 KD#1	AHCYL1 KD#2	DJ-1 KD	DJ-1 KD+AHCYL1	DJ-1 KD+AHCYL1
1st	19.78	24.93	18.85	62.91	31.6	36.14
2nd	26.65	28.25	24.43	57.14	40.19	28.7
3rd	21.75	29.59	13.95	50.57	45.12	12.12

Figure 7a

		Relative Tumor Volume							
Ctrl KD+vehicle	mice	2 day	4 day	6 day	8 day	10 day	12 day	14 day	16 day
	1	2.17	3.09	5.31	6.34	8.71	10.15	13.18	16.52
	2	1.77	2.89	4.75	7.24	10.46	11.33	16.51	19.19
	3	1.47	2.40	3.29	4.12	5.81	6.14	8.55	9.92
	4	2.76	4.16	5.46	8.12	10.31	12.56	15.09	18.67
	5	1.43	2.39	4.02	4.74	6.54	7.19	9.71	12.86
	6	1.78	3.00	3.60	4.73	7.59	8.67	12.31	15.11
	average	1.90	2.99	4.40	5.88	8.24	9.34	12.56	15.38
	SEM	0.20	0.26	0.37	0.65	0.79	1.00	1.25	1.45
Ctrl KD+PE	mice	2 day	4 day	6 day	8 day	10 day	12 day	14 day	16 day
	1	1.43	2.18	3.79	4.66	5.83	6.28	8.78	11.08
	2	1.44	1.81	2.67	3.68	4.38	4.83	6.14	7.10
	3	2.25	4.32	5.81	8.26	9.19	10.24	16.44	20.11
	4	1.20	1.98	3.19	4.18	5.01	5.74	7.98	9.88
	5	1.73	2.70	3.41	4.71	5.84	5.96	8.48	10.32
	6	2.13	2.92	4.18	5.84	8.99	11.27	15.53	18.89
	average	1.70	2.65	3.84	5.22	6.54	7.39	10.56	12.90
	SEM	0.17	0.38	0.45	0.67	0.84	1.09	1.76	2.16
DJ-1 KD+vehicle	mice	2 day	4 day	6 day	8 day	10 day	12 day	14 day	16 day
	1	2.12	3.21	5.98	7.59	10.75	11.31	15.59	18.98
	2	1.60	2.99	4.03	5.03	6.80	7.28	9.07	11.39
	3	2.64	3.15	4.27	6.65	11.52	12.40	20.20	25.52
	4	1.81	2.80	4.16	4.74	6.93	7.79	10.81	13.92
	5	0.96	1.54	2.39	7.26	12.65	14.39	20.07	27.54
	6	1.87	2.75	3.58	4.59	6.50	7.15	9.33	11.20
	average	1.83	2.74	4.07	5.98	9.20	10.05	14.18	18.09
	SEM	0.23	0.25	0.47	0.55	1.12	1.25	2.11	2.92
DJ-1 KD+PE	mice	2 day	4 day	6 day	8 day	10 day	12 day	14 day	16 day
	1	1.16	1.35	1.50	1.83	2.15	2.73	4.03	4.51
	2	1.11	1.25	1.93	2.16	3.08	3.40	4.11	5.10
	3	1.03	1.49	2.59	3.01	4.05	4.21	5.43	6.92
	4	1.14	1.78	3.15	3.34	4.09	4.33	5.12	6.14
	5	1.49	2.35	2.94	3.28	3.77	3.92	4.80	6.30
	6	1.23	1.33	1.43	1.74	2.71	2.82	3.19	3.84
	average	1.19	1.59	2.26	2.56	3.31	3.57	4.45	5.47
	SEM	0.07	0.17	0.30	0.30	0.32	0.28	0.34	0.48

Figure 7e

PARK7 mRNA level				
mice	Ctrl KD+vehicle	DJ-1 KD+vehicle	Ctrl KD+PE	DJ-1 KD+PE
1	1.207481	0.196827	1.539541	0.1774519
2	1.113422	0.1871154	1.314487	0.1632891
3	1.106497	0.1483421	1.124669	0.1243519
4	1.092021	0.1334157	0.9340036	0.1187912
5	0.7994058	0.1017429	0.6942369	0.08977764
6	0.7700372	0.05823378	0.6775973	0.08388171

PTGS2 mRNA level				
mice	Ctrl KD+vehicle	DJ-1 KD+vehicle	Ctrl KD+PE	DJ-1 KD+PE
1	1.832208	1.756962	3.217819	8.050528
2	0.8371646	1.398697	1.666803	3.342861
3	0.4770073	1.893537	0.5296398	4.044842
4	0.444447	0.8559411	0.7448817	3.226753
5	1.942727	3.237956	1.771637	8.236796
6	1.582914	1.054883	1.665648	4.62061

Figure 7f

4-HNE(nmol/mg)				
mice	Ctrl KD+vehicle	DJ-1 KD+vehicle	Ctrl KD+PE	DJ-1 KD+PE
1	1.139646045	2.165889809	0.669902618	4.284894045
2	2.181755545	1.45879763	2.782421569	2.754146129
3	2.334313446	1.658056506	1.295664879	4.254053616
4	1.273749286	1.71565645	1.299903816	2.201561762
5	1.343446638	1.494926061	2.229990034	2.903064023
6	1.969353081	2.556705383	2.467343977	3.22327044

Figure 7g

Hcy(nmol/mg)				
mice	Ctrl KD+vehicle	DJ-1 KD+vehicle	Ctrl KD+PE	DJ-1 KD+PE
1	4.183274	3.236154	4.090065	4.551804
2	4.899978	3.542062	3.463634	2.80992
3	4.913774	4.096108	4.197987	2.078216
4	5.921227	4.614164	5.916151	3.254079
5	6.144533	5.123966	7.247113	3.884564
6	6.336224	5.258078	7.97195	3.654856

Supplementary Figure 1b

A549						
Lipid ROS	DMSO			Erastin		
	Ctrl KD	DJ-1 KD#1	DJ-1 KD#2	Ctrl KD	DJ-1 KD#1	DJ-1 KD#2
1st	2.66	2.8	2.1	14.75	24.03	22.53
2nd	2.34	3.35	2.18	11.98	28.77	16.2
3rd	4.32	2.26	1.67	14.06	34.49	19.68

H292						
Lipid ROS	DMSO			Erastin		
	Ctrl KD	DJ-1 KD#1	DJ-1 KD#2	Ctrl KD	DJ-1 KD#1	DJ-1 KD#2
1st	1.9	11.78	9.44	13.04	60.97	30.58
2nd	5.33	11.55	3.12	17.33	37.49	39.4
3rd	5.21	10.23	4.11	11.39	36.75	26.25

H838						
Lipid ROS	DMSO			Erastin		
	Ctrl KD	DJ-1 KD#1	DJ-1 KD#2	Ctrl KD	DJ-1 KD#1	DJ-1 KD#2
1st	2.18	9.39	10.43	17.21	48.85	37.63
2nd	2.22	3.85	8.77	20.16	42.09	29.93
3rd	3.96	3.41	9.02	15.45	37.7	38.24

Supplementary Figure 1c

H1299				
Lipid ROS	DMSO		Sorafenib	
	Ctrl KD	DJ-1 KD#1	Ctrl KD	DJ-1 KD#1
1st	2.42	6.09	16.61	31.89
2nd	3.14	9.89	16.64	29.54
3rd	3.16	6.49	12.95	26.52

H1299						
Lipid ROS	DMSO			RSL-3		
	Ctrl KD	DJ-1 KD#1	DJ-1 KD#2	Ctrl KD	DJ-1 KD#1	DJ-1 KD#2
1st	1.86	2.28	1.88	24.64	42.66	34.23
2nd	1.9	8.21	3.53	27.06	44.98	39.19
3rd	1.25	2.48	1.37	27.16	44.8	38.18

H1299									
Lipid ROS	DMSO			ML210-25nM			ML210-50nM		
	Ctrl KD	DJ-1 KD#1	DJ-1 KD#2	Ctrl KD	DJ-1 KD#1	DJ-1 KD#2	Ctrl KD	DJ-1 KD#1	DJ-1 KD#2
1st	2.26	2.75	2.43	15.74	31.03	32.82	32.75	57.22	50.92
2nd	3.96	8.02	6.77	12.8	28.87	24.08	32.19	44.12	49.49
3rd	2.53	7.24	4.86	21.27	42.93	37.43	39	51.83	57.61

Supplementary Figure 1e

Cell Viability%	Ctrl KD			DJ-1 KD#1			DJ-1 KD#2		
Erastin(μM)	1st	2nd	3rd	1st	2nd	3rd	1st	2nd	3rd
0.0	100.0	100.0	100.0	100.0	100.0	100.0	100.0	100.0	100.0
1.0	83.8	80.4	91.7	43.7	52.9	47.4	63.5	71.8	66.3
2.0	63.2	65.6	76.1	19.1	10.0	20.6	20.7	30.3	31.4
4.0	33.7	34.2	44.2	9.1	10.2	11.6	9.7	23.8	24.6
Cell Viability%	Ctrl KD+Fer-1			DJ-1 KD#1+Fer-1			DJ-1 KD#2+Fer-1		
Erastin(μM)	1st	2nd	3rd	1st	2nd	3rd	1st	2nd	3rd
0.0	94.2	98.5	86.9	87.5	105.2	82.2	102.2	109.3	84.7
1.0	86.4	107.1	90.6	100.7	104.0	74.0	100.1	117.9	84.6
2.0	95.3	113.5	89.9	93.0	108.0	78.0	96.8	116.6	92.8
4.0	92.0	110.1	88.8	96.6	109.6	77.4	111.7	115.2	88.4

Supplementary Figure 2b

A2780						
Lipid ROS%	E-0		E-1.25		E-2.5	
	pCDH	DJ-1	pCDH	DJ-1	pCDH	DJ-1
1st	1.22	1.59	42.36	41.43	65.34	64.4
2nd	1.21	1.62	39.45	34.79	68.4	68.94
3rd	1.67	2.52	38.47	45.16	71.26	65.22

A2780						
cell viability%	E-0		E-1.25		E-2.5	
	pCDH	DJ-1	pCDH	DJ-1	pCDH	DJ-1
1st	100	100	52.67345	43.16028	11.45647	10.3921
2nd	100	100	51.7587	54.04276	9.236475	11.3859
3rd	100	100	61.10212	56.2444	10.49083	9.555048

Supplementary Figure2c

786-O						
Lipid ROS%	E-0		E-1.25		E-2.5	
	pCDH	DJ-1	pCDH	DJ-1	pCDH	DJ-1
1st	2.97	0.84	37.28	42.68	75.64	90.25
2nd	2.22	1.56	35.12	34.67	82.27	72.08
3rd	2.56	4.46	35.57	33.09	86.39	75.35

786-O						
cell viability%	E-0		E-1.25		E-2.5	
	pCDH	DJ-1	pCDH	DJ-1	pCDH	DJ-1
1st	100	100	63.83526	64.73385	8.703798	9.502277
2nd	100	100	69.25994	69.1485	9.881099	8.089379
3rd	100	100	66.6741	63.49688	11.02897	13.04159

Supplementary Figure 2d

KHOS								
Lipid ROS%	E-0		E-1.25		E-2.5		E5	
	pCDH	DJ-1	pCDH	DJ-1	pCDH	DJ-1	pCDH	DJ-1
1st	2.75	2.72	20.75	25.23	41.3	34.61	50.86	47.12
2nd	3.55	2.56	18.16	19.68	46.84	49.52	50.98	58.1
3rd	5.22	5.83	13.08	12.38	41.83	49.66	46.53	51.59

KHOS								
cell viability%	E-0		E-1.25		E-2.5		E5	
	pCDH	DJ-1	pCDH	DJ-1	pCDH	DJ-1	pCDH	DJ-1
1st	100	100	54.1580	53.99726	39.48816	42.41154	27.3749	25.86352
2nd	100	100	63.3250	58.1700	41.46363	42.84595	31.45372	28.66266
3rd	100	100	53.2122	54.86722	40.89482	41.10535	27.13766	28.84057

Supplementary Figure 2e

H1299								
Lipid ROS%	E-0		E-2		E-4		E8	
	pCDH	DJ-1	pCDH	DJ-1	pCDH	DJ-1	pCDH	DJ-1
1st	2.02	3.32	18.31	14.91	37.94	34.64	59.51	63.77
2nd	4.66	4.32	17.28	13.25	31.82	25.99	51.21	47.85
3rd	3.79	1.95	22.6	27.02	32.17	34.49	61.45	55.72

H1299								
cell viability%	E-0		E-2		E-4		E8	
	pCDH	DJ-1	pCDH	DJ-1	pCDH	DJ-1	pCDH	DJ-1
1st	100	100	53.48798	52.27309	33.7431	34.9595	23.39023	26.67361
2nd	100	100	54.37801	50.45258	36.08823	33.3927	21.51788	25.18962
3rd	100	100	58.71377	55.2554	31.32473	36.04474	17.69928	16.00602

Supplementary Figure 2f

MEF(DJ-1 -/-)						
Cell Viability%	DMSO		Sorafenib-20		Sorafenib-50	
	pCDH	DJ-1	pCDH	DJ-1	pCDH	DJ-1
1st	100	100	67.93388	73.84278	41.39893	63.62573
2nd	100	100	63.23328	88.19159	37.18866	57.35727
3rd	100	100	67.467	86.79115	43.3272	64.4353

MEF(DJ-1 -/-)										
Cell Viability%	DMSO		RSL3-50		RSL3-100		RSL3-200		RSL3-400	
	pCDH	DJ-1	pCDH	DJ-1	pCDH	DJ-1	pCDH	DJ-1	pCDH	DJ-1
1st	100	100	82.94	84.26	69.68	66.37	51.25	50.10	40.58	36.10
2nd	100	100	83.00	81.21	63.87	62.69	44.91	43.25	34.12	37.92
3rd	100	100	79.88	77.01	62.80	63.29	48.48	52.04	32.75	36.60

Supplementary Figure 2g

MEF(DJ-1 -/-)								
Lipid ROS%	DMSO		ML210-200		ML210-400		ML210-800	
	pCDH	DJ-1	pCDH	DJ-1	pCDH	DJ-1	pCDH	DJ-1
1st	2.92	2.52	12.37	9.57	18.44	15.34	26.74	28.71
2nd	3.65	6.91	16.05	15.69	17.44	17.57	27.92	29.4
3rd	2.79	4.8	15.29	11.64	19.86	16.5	28.15	31.6

MEF(DJ-1 -/-)										
Cell Viability%	DMSO		ML210-200		ML210-400		ML210-800		ML210-1600	
	pCDH	DJ-1	pCDH	DJ-1	pCDH	DJ-1	pCDH	DJ-1	pCDH	DJ-1
1st	100	100	90.88	89.93	72.39	63.20	24.70	20.04	5.21	5.72
2nd	100	100	74.00	70.92	56.88	55.09	18.53	24.05	1.41	6.47
3rd	100	100	92.43	88.15	75.55	64.51	23.43	16.19	1.98	1.18

Supplementary Figure 3b

Cystine uptake(% of control)			
	Ctrl KD	DJ-1 KD#1	DJ-1 KD#2
1st	100	94.854	95.2943
2nd	100	102.1363	107.3985
3rd	100	107.3046	108.6444

Supplementary Figure 3c

Cysteine(nmol/mg)			
	Ctrl KD	DJ-1 KD#1	DJ-1 KD#2
1st	38.23212	38.83099	44.56377
2nd	35.71423	36.07329	37.84653
3rd	42.27617	51.71946	41.79197

Supplementary Figure 3d

GSH(Fold Change)	DMSO					
	DJ-1 WT#1	DJ-1 WT#2	DJ-1 WT#3	DJ-1 KO#1	DJ-1 KO#2	DJ-1 KO#3
1st	1	1.00	1.00	0.94	0.95	0.93
2nd	1	1.00	1.00	0.90	1.14	1.10
3rd	1	1.00	1.00	1.15	0.97	1.09
GSH(Fold Change)	Erastin					
	DJ-1 WT#1	DJ-1 WT#2	DJ-1 WT#3	DJ-1 KO#1	DJ-1 KO#2	DJ-1 KO#3
1st	0.73	0.65	0.80	0.16	0.16	0.17
2nd	0.70	0.70	0.64	0.14	0.12	0.14
3rd	0.61	0.67	0.42	0.17	0.17	0.21

Supplementary Figure 3g

Lipid ROS%	DMSO		Erastin		Erastin+Hcy		Erastin+GSH	
	Ctrl	BSO	Ctrl	BSO	Ctrl	BSO	Ctrl	BSO
1st	1.96	2.1	16.67	33.53	3.4	29.21	3.07	5.62
2nd	2.22	3.29	24.34	43.62	5.37	43.31	5.52	7.55
3rd	2.31	2.84	24.62	42.85	7.14	33.55	5.13	7.61

Supplementary Figure 4b

Relative level	SAM		SAH		Hcy	
	Ctrl KD	DJ-1 KD	Ctrl KD	DJ-1 KD	Ctrl KD	DJ-1 KD
1st	1	1.08	1.00	0.95	1.00	0.57
2nd	1	1.02	1.00	1.16	1.00	0.73
3rd	1	1.09	1.00	1.21	1.00	0.61

Supplementary Figure 4c

SAM				
Percentage of total	M+0		M+1	
	Ctrl KD	DJ-1 KD	Ctrl KD	DJ-1 KD
1st	1.96	1.78	98.04	98.22
2nd	4.46	3.04	95.54	96.96
3rd	2.13	2.36	97.87	97.64
SAH				
Percentage of total	M+0		M+1	
	Ctrl KD	DJ-1 KD	Ctrl KD	DJ-1 KD
1st	15.95	13.46	84.05	86.54
2nd	26.29	29.30	73.71	70.70
3rd	17.34	14.18	82.66	85.82
Hcy				
Percentage of total	M+0		M+1	
	Ctrl KD	DJ-1 KD	Ctrl KD	DJ-1 KD
1st	97.71	99.11	2.29	0.89
2nd	97.94	99.22	2.06	0.78
3rd	98.14	99.30	1.86	0.70

Hcy(M+1)		
Percentage of total	M+1	
	Ctrl KD	DJ-1 KD
1st	2.29	0.89
2nd	2.06	0.78
3rd	1.86	0.70

Supplementary Figure 4d

PANC1				
Hcy(nmol/mg)	DMSO		Erastin	
	Ctrl KD	DJ-1 KD	Ctrl KD	DJ-1 KD
1st	6.000191	3.501541	4.507901	3.07959
2nd	6.009448	4.302024	5.025589	3.803704
3rd	4.789776	2.542913	4.200386	2.10897

Supplementary Figure 4e

H1299				
SAH(nmol/mg)	DMSO		Erastin	
	Ctrl KD	DJ-1 KD	Ctrl KD	DJ-1 KD
1st	5.52114	4.838656	4.270894	4.915921
2nd	4.284214	4.261959	4.273416	4.930195
3rd	4.022115	4.12224	4.044125	4.122955

PANC1				
SAH(nmol/mg)	DMSO		Erastin	
	Ctrl KD	DJ-1 KD	Ctrl KD	DJ-1 KD
1st	3.235745	3.207916	3.336828	2.970742
2nd	3.869756	4.102205	4.270276	3.040274
3rd	3.806759	3.774019	3.92568	3.49499

MEF(DJ-1 -/-)				
SAH(nmol/mg)	DMSO		Erastin	
	pCDH	DJ-1	pCDH	DJ-1
1st	1.895573	1.877357	1.824958	0.938977
2nd	1.748835	1.800224	1.456403	1.53506
3rd	1.974413	2.295149	2.184557	1.880867

Supplementary Figure 4f

MEF(DJ-1 -/-)								
HCY(nmol/mg)	DMSO				Erastin			
	pCDH	M26I	A104T	WT	pCDH	M26I	A104T	WT
1st	3.55	3.92	3.30	4.36	3.60	3.49	3.76	4.59
2nd	3.17	3.31	3.37	4.73	3.38	3.46	3.52	4.63
3rd	3.67	3.44	3.08	5.60	3.33	3.35	3.47	5.99

MEF(DJ-1 -/-)								
SAH(nmol/mg)	DMSO				Erastin			
	pCDH	M26I	A104T	WT	pCDH	M26I	A104T	WT
1st	1.58	1.59	1.61	1.63	1.85	1.87	1.80	1.79
2nd	1.62	1.48	1.46	1.83	1.47	1.48	1.88	1.60
3rd	1.43	1.60	1.69	1.44	1.86	1.24	1.65	1.56

Supplementary Figure 4g

MEF DJ-1 -/-								
Hcy(nmol/mg)	met(-) SAH(-)				met(-) SAH(+)			
	pCDH	M26I	A104T	DJ-1	pCDH	M26I	A104T	DJ-1
1st	1	1.11	0.96	1.44	1.19	1.25	1.30	1.70
2nd	1	0.96	0.91	1.14	1.27	1.31	1.10	2.22
3rd	1	1.06	1.06	1.34	1.18	1.22	1.24	2.06

Supplementary Figure 4i

time	pCDH	M26I	A104T	WT
0:00:00	-180.9	-68.4	171.18	50.3
0:00:45	-163.2	11.8	463.82	46
0:01:30	-174.6	87.3	504.01	81.8
0:02:15	-150.6	65.6	239.81	124
0:03:00	-100.2	44.7	487.79	220
0:03:45	-38.13	49	305.23	243
0:04:30	38.302	101	193.77	250
0:05:15	12.13	253	611.22	333
0:06:00	56.971	280	493.95	465
0:06:45	98.411	91.9	262.09	585
0:07:30	138.49	274	694.06	659
0:08:15	14.047	307	554.29	658
0:09:00	133.93	197	207.59	736
0:09:45	14.036	291	729.96	742
0:10:30	204.23	481	466.84	955
0:11:15	196.68	456	654.44	955
0:12:00	188.08	445	761.81	977
0:12:45	83.549	457	274.54	995
0:13:30	196.94	480	651.66	998
0:14:15	278.21	574	551.75	1124
0:15:00	340.16	615	604.73	1259
0:15:45	139.3	679	731.23	1331
0:16:30	225.59	711	566.45	1479
0:17:15	315.82	739	584.4	1401
0:18:00	304.49	737	747.94	1419
0:18:45	357.8	737	658.47	1474
0:19:30	344.72	808	591.82	1508
0:20:15	323.03	796	840.22	1617
0:21:00	304.65	541	813.45	1602
0:21:45	374.13	553	702.1	1647
0:22:30	437.87	483	717.89	1672
0:23:15	402.99	610	789.88	1611
0:24:00	477.57	638	839.49	1730
0:24:45	446.52	750	884.23	1827
0:25:30	451.86	876	1031.6	1726
0:26:15	456.55	965	974.74	1734
0:27:00	572.7	1008	915.99	1856

0:27:45	523.41	1089	968.44	1810
0:28:30	499.08	1140	884.03	1833
0:29:15	577.73	1161	906.64	1829
0:30:00	561.92	1205	1090.4	1765
0:30:45	546.15	1198	1054.2	1920
0:31:30	572.73	1132	1056.3	2127
0:32:15	604.61	1065	977.3	1897
0:33:00	586.11	1096	992.73	2081
0:33:45	734.85	1089	924.55	2221
0:34:30	766.99	1141	1093.4	2159
0:35:15	721.11	1176	832.21	2137
0:36:00	904.4	1280	1116.4	2181
0:36:45	815.6	1113	981.66	2167
0:37:30	745.78	1270	1159	2163
0:38:15	866.42	1216	1049.3	2277
0:39:00	1014.7	1394	1106.1	2385
0:39:45	549.55	1311	1076.6	2433
0:40:30	828.13	1360	1080.5	2635
0:41:15	623.69	1455	1026.7	2660
0:42:00	864.76	1473	1078.2	2717
0:42:45	714.98	1470	1217.4	2656
0:43:30	826.41	1417	1222.2	2782
0:44:15	638.37	1444	1226.5	2915
0:45:00	946.24	1401	1261	3041

Supplementary Figure 5b

time	IgG	Ctrl KD	DJ-1 KD#1	DJ-1 KD#2
0:00:00	46.865	401.412	250.203	457.556
0:00:45	30.992	385.915	310.791	242.738
0:01:30	84.172	458.471	307.051	260.922
0:02:15	83.363	458.962	320.105	315.118
0:03:00	107.259	381.023	284.141	302.986
0:03:45	99.771	369.06	293.548	268.584
0:04:30	72.4	364.86	318.903	342.246
0:05:15	80.385	398.254	335.634	326.251
0:06:00	21.257	437.472	307.183	352.304
0:06:45	84.719	450.231	268.831	360.061
0:07:30	77.882	448.086	290.063	317.012
0:08:15	77.141	466.635	242.002	278.417
0:09:00	65.411	426.478	231.772	373.74
0:09:45	88.194	472.179	206.194	347.082
0:10:30	56.322	429.192	253.747	300.585
0:11:15	81.509	563.701	283.843	316.233
0:12:00	51.653	525.254	314.531	405.731
0:12:45	92.71	494.955	353.353	363.925
0:13:30	81.634	552.058	379.972	353.303
0:14:15	63.959	536.916	323.328	366.33
0:15:00	55.585	539.207	342.883	371.056
0:15:45	36.545	626.769	313.669	415.173
0:16:30	99.01	592.831	396.571	483.036
0:17:15	52.551	601.86	309.401	461.123
0:18:00	77.149	664.965	393.804	387.832
0:18:45	59.653	640.541	372.903	469.877
0:19:30	52.356	617.002	418.294	445.895
0:20:15	57.884	605.161	393.029	357.169
0:21:00	15.286	706.385	437.529	428.276
0:21:45	30.467	710.452	396.112	390.702
0:22:30	26.335	736.194	474.042	416.598
0:23:15	20.292	719.556	386.254	421.796
0:24:00	111.675	816.473	419.669	430.656
0:24:45	101.429	849.861	421.934	378.664
0:25:30	90.19	750.623	442.409	388.484
0:26:15	131.386	829.026	496.435	377.918
0:27:00	87.399	826.242	410.672	408.435
0:27:45	130.553	870.613	382.604	454.364
0:28:30	115.448	819.056	425.508	443.936
0:29:15	118.048	905.787	417.292	496.782
0:30:00	110.213	952.07	406.58	366.839
0:30:45	95.655	979.467	494.625	460.466
0:31:30	233.774	870.915	443.324	475.682
0:32:15	95.855	913.334	438.42	451.338
0:33:00	96.629	973.191	432.536	473.153

0:33:45	209.695	945.776	558.951	433.483
0:34:30	85.28	1051.613	457.979	421.624
0:35:15	171.206	1106.193	474.322	374.717
0:36:00	172.359	1083.728	531.928	341.703
0:36:45	192.545	1035.101	529.01	437.601
0:37:30	157.9	1167.415	464.081	420.461
0:38:15	115.316	1052.083	517.249	398.992
0:39:00	95.31	978.978	482.99	451.663
0:39:45	70.426	1056.732	502.562	407.61
0:40:30	130.304	1078.536	521.575	521.35
0:41:15	191.877	1056.639	487	516.224
0:42:00	193.696	1020.901	528.819	498.482
0:42:45	202.128	969.655	519.107	465.362
0:43:30	188.159	1168.702	546.242	476.856
0:44:15	130.312	1096.857	525.63	404.166
0:45:00	139.316	1209.678	535.388	520.061

Supplementary Figure 5c

SAH administration								
Lipid ROS%	DMSO				Erastin			
	Ctrl KD	Ctrl KD+SAHH	DJ-1 KD	DJ-1 KD+SAHH	Ctrl KD	Ctrl KD+SAHH	DJ-1 KD	DJ-1 KD+SAHH
1st	2.06	5.97	4.68	5.25	22.81	23.17	56.78	37.07
2nd	1.97	2.81	6.95	8.14	20.58	23.86	53.26	31.38
3rd	4.75	6.45	9.11	11.37	12.24	21.47	64.01	34.25

Supplementary Figure 5d

SAH administration								
Cell Viability%	DMSO				Erastin			
	Ctrl KD	Ctrl KD+SAHH	DJ-1 KD	DJ-1 KD+SAHH	Ctrl KD	Ctrl KD+SAHH	DJ-1 KD	DJ-1 KD+SAHH
1st	100	100	100	100	69.03	64.37	31.96	64.10
2nd	100	100	100	100	68.80	74.19	25.66	55.33
3rd	100	100	100	100	85.50	85.83	21.87	63.42

Supplementary Figure 5e

time	r-SAHH	r-SAHH+r-DJ-1	r-SAHH+BSA
0:00:00	24462.91	16671.52	16650.73
0:00:45	28418.75	20454.03	20220.86
0:01:30	36007.32	27103.25	27049.37
0:02:15	43100.18	33300.17	32963.07
0:03:00	49521.42	39148.89	39143.71
0:03:45	55645.18	45351.59	45521.72
0:04:30	59749.86	49593.37	50555.88
0:05:15	64618.05	54716.26	56035.1
0:06:00	68500.29	59847.77	60709.53
0:06:45	73888.52	65161.47	65976.34
0:07:30	75571.51	68633.89	69058.56
0:08:15	76415.5	69408.88	69745.27
0:09:00	78943.32	73474.35	71763.75
0:09:45	80804.11	75644.29	74853.5
0:10:30	81440.24	77433.67	78739.12
0:11:15	81328.3	79285.94	77699.76
0:12:00	82287.23	78277.48	79109.38
0:12:45	82786.91	82395.82	81286.81
0:13:30	81995.68	81760.99	80732.26
0:14:15	83690.06	84553.51	83108.03
0:15:00	82127.54	83183.07	83654.58
0:15:45	82547.12	83650.72	83616.93
0:16:30	83278.46	83818.1	84408.84
0:17:15	82854.75	84502.48	84987.27
0:18:00	82221.49	82502.08	83914.13
0:18:45	82418.25	82890.89	83868.63
0:19:30	81117.44	84079.54	85182.44
0:20:15	82175.69	85766.52	84790.68
0:21:00	78389.25	82022.21	83563.6
0:21:45	79470.62	81097.93	84492.15
0:22:30	78819.19	80619.58	82320.79
0:23:15	78991.26	81179.83	83023.43
0:24:00	78680.92	83229.82	81746.34
0:24:45	77937.02	84347.4	82580.92
0:25:30	78072.44	83664.8	81823.29
0:26:15	77548.02	83829.35	82540.59
0:27:00	77859.01	81322.87	83666.17
0:27:45	77226.37	80786.06	81917.73
0:28:30	75892.12	82562.83	80987.07
0:29:15	76828.89	82207.93	82985.16
0:30:00	75672.18	81997.66	82578.99
0:30:45	75656.31	82091.66	80419.59
0:31:30	75082.36	82079.86	82138.1
0:32:15	73816.29	78052.22	80745.94

0:33:00	73698.93	81312.09	81284.94
0:33:45	74554.67	81446.67	83863.68
0:34:30	72902.24	81948.56	81170.09
0:35:15	72310.41	77995.37	79774.69
0:36:00	71178.99	78913.85	77957.13
0:36:45	72270.05	79544.81	82387.21
0:37:30	70889.2	79736.68	78228.65
0:38:15	70692.62	76536.1	77904.96
0:39:00	70788.3	79465.62	78328.88
0:39:45	70146.43	79538.44	78677.01
0:40:30	69411.09	77933.24	77535.89
0:41:15	69952.02	77141.35	75555.85
0:42:00	68226	77986.35	76288
0:42:45	68790.82	76839.94	77379.47
0:43:30	69031.51	78059.89	77400.33
0:44:15	66982.36	75705.05	74300.12
0:45:00	67145.83	75867.09	75093.71

Supplementary Figure 6a

Flag/HA			
Relative levels	Ctrl	DJ-1 OE	DJ-1 KD
1st	1	0.32	1.18
2nd	1	0.45	1.25
3rd	1	0.77	1.23

Supplementary Figure 6b

SAHH/AHCYL1			
Relative levels	Ctrl KD	DJ-1 KD#1	DJ-1 KD#2
1st	1	1.39	1.20
2nd	1	1.09	1.12
3rd	1	1.28	1.07

Supplementary Figure 6d

Cell Viability%	DMSO					
	Ctrl KD	AHCYL1 KD#1	AHCYL1 KD#2	DJ-1 KD	DJ-1 KD+AHCYL1	DJ-1 KD+AHCYL1
1st	100	100	100	100	100	100
2nd	100	100	100	100	100	100
3rd	100	100	100	100	100	100
Cell Viability%	Erastin					
	Ctrl KD	AHCYL1 KD#1	AHCYL1 KD#2	DJ-1 KD	DJ-1 KD+AHCYL1	DJ-1 KD+AHCYL1
1st	59.64	60.93	61.01	21.14	38.62	42.50
2nd	45.56	47.33	46.42	17.31	39.52	37.50
3rd	58.20	59.83	63.92	23.56	47.03	40.22

Supplementary Figure 6e

PARK7		Ctrl KD	AHCYL2 KD#1	AHCYL2 KD#2	DJ-1 KD	DJ-1 KD+AHCYL2	DJ-1 KD+AHCYL2
	1st	1	1.02	0.96	0.11	0.18	0.16
	2nd	1	1.17	0.94	0.14	0.19	0.17
	3rd	1	1.10	1.09	0.20	0.15	0.20
	average	1	1.09	1.00	0.15	0.17	0.17
	SD	0	0.07	0.08	0.04	0.02	0.02

AHCYL2		Ctrl KD	AHCYL2 KD#1	AHCYL2 KD#2	DJ-1 KD	DJ-1 KD+AHCYL2	DJ-1 KD+AHCYL2
	1st	1	0.33	0.22	0.89	0.35	0.23
	2nd	1	0.22	0.28	1.03	0.33	0.33
	3rd	1	0.27	0.21	0.96	0.33	0.34
	average	1	0.27	0.24	0.96	0.34	0.30
	SD	0	0.06	0.04	0.07	0.01	0.06

Supplementary Figure 6f

Lipid ROS	DMSO					
	Ctrl KD	AHCYL2 KD#1	AHCYL2 KD#2	DJ-1 KD	DJ-1 KD+AHCYL2 KD#1	DJ-1 KD+AHCYL2 KD#2
1st	4.49	4	2.26	7.74	9.25	11.51
2nd	1.93	5.43	3.43	3.99	7.29	8.23
3rd	3.55	1.42	2.66	4.25	12.43	10.36
Lipid ROS	Erastin					
	Ctrl KD	AHCYL2 KD#1	AHCYL2 KD#2	DJ-1 KD	DJ-1 KD+AHCYL2 KD#1	DJ-1 KD+AHCYL2 KD#2
1st	21.85	22.09	16.13	49.82	48.82	45.31
2nd	18.19	25.09	17.1	43.41	54.38	45.17
3rd	18.16	23.75	20.37	42.65	52.99	45.51

Supplementary Figure 6g

Cell Viability%	Ctrl KD			AHCYL2 KD#1			AHCYL2 KD#2		
Erastin(μM)	1st	2nd	3rd	1st	2nd	3rd	1st	2nd	3rd
0.0	100.0	100.0	100.0	100.0	100.0	100.0	100.0	100.0	100.0
1.0	97.1	88.7	88.2	99.8	87.3	87.5	75.7	82.9	88.7
2.0	73.4	66.7	59.5	76.4	78.0	63.0	63.0	75.4	62.0
4.0	49.7	50.2	48.4	50.8	49.6	55.1	43.4	47.3	49.8
Cell Viability%	DJ-1 KD			DJ-1 KD+AHCYL2 KD#1			DJ-1 KD+AHCYL2 KD#2		
Erastin(μM)	1st	2nd	3rd	1st	2nd	3rd	1st	2nd	3rd
0.0	100.0	100.0	100.0	100.0	100.0	100.0	100.0	100.0	100.0
1.0	63.2	66.3	72.5	67.7	73.3	73.5	59.0	67.4	74.0
2.0	46.4	42.0	43.8	33.6	40.3	41.4	47.6	36.6	52.5
4.0	24.0	26.6	23.8	21.0	14.6	29.9	13.1	24.5	30.1

Supplementary Figure 7a

weight				
	Vehicle		PE	
	Ctrl KD	DJ-1 KD	Ctrl KD	DJ-1 KD
1	1.42	1.17	0.95	0.43
2	0.94	1.71	1.19	0.58
3	1.75	0.55	0.68	0.47
4	0.50	0.70	0.30	0.74
5	1.42	0.70	2.39	1.44
6	1.09	2.47	0.91	0.22
average	1.19	1.22	1.07	0.65
SEM	0.18	0.30	0.29	0.17
inhibition%		-2.7111692	9.6211387	45.485833

day 16 RTV				
	Vehicle		PE	
	Ctrl KD	DJ-1 KD	Ctrl KD	DJ-1 KD
1	16.52	11.08	18.98	4.51
2	19.19	7.10	11.39	5.10
3	9.92	20.11	25.52	6.92
4	18.67	9.88	13.92	6.14
5	12.86	10.32	27.54	6.30
6	15.11	18.89	11.20	3.84
average	15.38	12.90	18.09	5.47
T/C	-	0.84	1.18	0.36

Supplementary Figure 7b

Ki67 %				
	Vehicle		PE	
	Ctrl KD	DJ-1 KD	Ctrl KD	DJ-1 KD
1	60.91	55.48	64.03	59.71
2	52.36	58.84	51.17	58.19
3	58.10	51.87	55.16	62.71
4	70.16	48.74	57.14	60.91
5	56.06	66.00	53.82	52.07
6	55.00	55.93	50.25	53.43

Supplementary Figure7c

tunel %					
	Vehicle		PE		positive ctrl
	Ctrl KD	DJ-1 KD	Ctrl KD	DJ-1 KD	
1	0.91	0.78	2.41	3.33	31.90
2	1.24	5.20	5.08	0.86	38.10
3	4.34	4.47	2.29	2.62	22.14
4	2.71	3.34	2.94	2.65	
5	1.09	1.42	4.62	5.07	
6	3.40	1.45	2.37	1.79	
